# Altered sncRNA Signatures in Semen Extracellular Vesicles Between Patients with Benign and Malignant Prostate Disease as Potential Non-Invasive Biomarkers in the PSA Grey Zone

**DOI:** 10.3390/ijms27146205

**Published:** 2026-07-11

**Authors:** Adriana Ferre-Giraldo, Dave Rojas-Calderón, Manel Castells, Helena Raurell, Clara Mayayo-Vallverdú, Esther Prat, Olga López-Rodrigo, Maurizio de Rocco-Ponce, Lluís Bassas, Francesc Vigués, Lauro Sumoy, Sara Larriba

**Affiliations:** 1 Human Molecular Genetics Group, Genes, Disease and Therapy Program-Bellvitge Biomedical Research Institute (IDIBELL), L’Hospitalet de Llobregat, 08908 Barcelona, Spain; aferre@idibell.cat (A.F.-G.); cmayayo@idibell.cat (C.M.-V.); eprat@idibell.cat (E.P.); 2 Immune-Inflammatory Processes Group, Genes, Disease and Therapy Program-Bellvitge Biomedical Research Institute (IDIBELL), L’Hospitalet de Llobregat, 08908 Barcelona, Spain; 3 High Content Genomics and Bioinformatics (HCGB)-Germans Trias I Pujol Research Institute (IGTP), 08916 Badalona, Spain; dacal7486@nmbu.no (D.R.-C.); hraurell@igtp.cat (H.R.); lsumoy@igtp.cat (L.S.); 4 Faculty of Chemistry, Biotechnology and Food Science, Norwegian University of Life Sciences, 1433 Ås, Norway; 5 Urology Service, Bellvitge University Hospital-ICS (Institut Català de la Salut), L’Hospitalet de Llobregat, 08908 Barcelona, Spain; mcastells@bellvitgehospital.cat (M.C.); fvigues@bellvitgehospital.cat (F.V.); 6 Neuroscience Program, Physiology Unit, IDIBELL, Department of Physiological Sciences, School of Medicine and Health Sciences, Institute of Neurosciences, University of Barcelona-IDIBELL, L’Hospitalet de Llobregat, 08907 Barcelona, Spain; 7 Laboratory of Seminology, Andrology Service-Fundació Puigvert, 08025 Barcelona, Spain; olopez@fundacio-puigvert.es (O.L.-R.); mderocco@fundacio-puigvert.es (M.d.R.-P.); lbassas@fundacio-puigvert.es (L.B.)

**Keywords:** prostate cancer, PSA, sncRNAs, isomiRs, tsRNAs, biomarker, semen extracellular vesicles, non-invasive prognosis/diagnosis

## Abstract

PSA testing plays an important role in the diagnostic workup of prostate cancer; despite this, its cancer specificity is a well-recognised limitation. Consequently, more reliable, non-invasive diagnostic tools that are capable of improving specificity while maintaining sensitivity are needed, thereby enabling better risk stratification, personalised patient management, and reduction in unnecessary invasive procedures. In this study, we characterised the small RNA profile—including miRNAs and tsRNAs—in seminal small extracellular vesicles (sEVs) using high-throughput sequencing to expand biomarker discovery for distinguishing benign from malignant prostate conditions in patients with moderately elevated PSA levels, a setting where non-invasive biomarkers are critically needed. Our analysis confirms a small number of differentially represented sncRNA transcripts contained in seminal sEVs between benign and malignant prostate disease, most of which are of low abundance. This result suggests that shared underlying molecular mechanisms are likely to occur between both prostate disease conditions, especially in patients with moderate PSA levels. Subsequent RT-qPCR analysis revealed differences in expression in PCa compared with healthy controls but not when compared with benign prostatic disease. Given the complexity of the underlying pathophysiological process and the heterogeneity in clinical phenotypes, this limitation was addressed through the application of multivariate approaches (including isomiRs or tRFs), which are proposed as fluid-based biomarkers for prostate cancer, collectively offering improved accuracy and enhancing the negative predictive value of PSA used in clinical settings.

## 1. Introduction

Prostate cancer (PCa) is one of the most prevalent malignancies in men, affecting one in six individuals. Detection typically relies on serum prostate-specific antigen (PSA) testing combined with physical examination of the prostate gland (digital rectal examination -DRE-) and/or a multiparametric magnetic resonance imaging (mpMRI) assessment. However, the limitations of PSA as a biomarker are well documented [1,2]. Although prostate-specific, PSA has low specificity for cancer, as benign conditions such as benign prostatic hyperplasia (BPH) and prostatitis can also increase PSA levels. Therefore, suspicious findings require confirmation through the invasive histological evaluation of prostate biopsies, and the severity or extent of involvement is determined using the modified Gleason score (GS) [3].

In this context, although PSA screening has improved PCa detection and reduced mortality, it has also contributed to overdiagnosis and unnecessary biopsies of benign conditions affecting the prostate. This fact is particularly relevant among patients with PSA levels in the 4–10 ng/mL range (also known as “grey zone”), where cancer detection rates are below 20% compared with >50% when PSA exceeds 10 ng/mL. Furthermore, PSA levels do not correlate with tumour aggressiveness or treatment response, highlighting the need for additional fluid biomarkers to eventually complement PSA and increase specificity.

Seminal plasma contains a unique population of small extracellular vesicles (sEVs) [4] secreted by the organs of the male reproductive system, including the prostate. The RNA cargo of seminal sEVs is enriched in small non-coding RNAs (sncRNAs), including microRNAs (miRNAs) and tRNA-derived small RNAs (tsRNAs) [4,5], which regulate gene expression and participate in a wide range of physiological and pathological processes (including cancer initiation and progression). Having in mind that the prostate is a major contributor of sEVs in semen, and therefore likely to harbour prostate disease-specific molecular content, we previously developed miRNA-based assays using sEVs from semen, which, when combined with PSA concentration in blood, are capable of predicting PCa and tumour severity in men with moderately elevated PSA levels [6]. This study predominantly identified miRNA expression differences between healthy individuals and PCa patients, while only a few miRNAs were differentially expressed between PCa and BPH individuals, by using RT-qPCR arrays (profiling 634 mature miRNAs).

The study of miRNAs can be complex and technically challenging. It is now well established that a single miRNA locus can give rise to multiple distinct naturally occurring variants (termed isomiRs); thus, individual miRNAs can be heterogeneous in length and/or sequence, influencing the specificity and efficiency of miRNA-mediated regulation. Most commercially available miRNA PCR-based assays are designed to target the canonical miRNA sequences (reference sequences annotated in databases), which in some cases may actually correspond to a less abundant variant within a miRgroup (clustering all isomiR variants that originate from a miRNA locus), rather than the most highly expressed or functionally dominant form in a given biological context. In addition to design biases, RT-qPCR strategies may exhibit unwanted cross-reactivity between closely related sequences [7], unlike small RNA sequencing (sRNAseq), which, by definition, discriminates between miRNA isoforms (isomiRs) and other small RNA species, such as piRNAs and tsRNAs, and enables detection of novel miRNAs. Consequently, sRNAseq profiling offers a more accurate and informative strategy with the potential to uncover new markers that would remain undetected by standard RT-qPCR arrays.

To improve biomarker discovery for distinguishing benign from malignant prostatic conditions in patients with moderately elevated levels of PSA—a clinical scenario in which the need to identify truly non-invasive biomarkers is particularly relevant—we sought to characterise the small RNA expression profile, including miRNA and tsRNA isoforms, in seminal sEVs by high-throughput sequencing. Specifically, we focused on identifying altered sncRNA signatures between BPH and PCa patients within the grey zone.

## 2. Results

### 2.1. Clinical Assessment of Individuals Included in the Study

The clinicopathological characteristics of all individuals included in the study are summarised in Table 1 and Supplementary Table S1. 

Both BPH and PCa groups presented similar age values (mean: 58.6 and 58.8 years, respectively), supporting their suitability as comparable groups for the study. Regarding pre-biopsy PSA levels, the majority of PCa cases and BPH controls (32 out of 37 individuals, 86.5%) fell within the diagnostic “grey zone” (4–10 ng/mL), and only five PCa individuals presented PSA values above this threshold (10.5–17.7 ng/mL), while all BPH controls remained within or below this range.

The histopathological evaluation of prostate biopsies showed that most PCa patients presented low- or intermediate-grade disease, with 13 out of 29 individuals (44.8%) classified as GS6 and 11 individuals (37.9%) as GS7 (3 + 4); only five patients (17.2%) showed higher-grade disease, GS7 (4 + 3) or GS8 (4 + 4). Samples were further stratified into prognostic groups according to the AJCC (American Joint Committee on Cancer) staging system. Under this classification, the majority of PCa samples (22 out of 29; 75.9%) were categorised as low/intermediate-risk tumours (stages I, IIA, IIB), whereas approximately a quarter of the samples (seven out of 29; 24.1%) were classified as high-risk tumours (stages IIC and IIIB), reflecting a more advanced or unfavourable prognosis. This staging system also captures the differential prognostic weight of the two Gleason 7 sub-patterns: organ-confined GS7 (3 + 4) cancers are included in stage IIB, while GS7 (4 + 3) cancers are included in stage IIC, consistent with the more aggressive behaviour associated with pattern 4 predominance.

Additionally, healthy individuals (HCt) were included as negative controls in RT-qPCR analysis: 11 vasectomised men (HCt_V; mean age: 40) and five normozoospermic non-vasectomised (HCt_noV; mean age: 39.8).

### 2.2. sRNAseq Reveals sncRNA Variants Associated with Prostate Malignancy in Semen sEVs

Semen sEVs were isolated by a microfiltration–ultracentrifugation procedure and were subsequently measured by nanoparticle tracking analysis. Total RNA obtained from 16 semen sEV samples belonging to individuals with benign disease (BPH, *n* = 4; mean age: 58.2 ± 5.32 years) and PCa patients (*n* = 12, mean age: 59.1 ± 5.55 years) with moderately altered PSA levels (4–18 ng/mL; BPH mean value: 6.02; PCa mean value: 8.21) was sequenced in depth to profile the expression of small RNAs (Supplementary Table S1). Clinical histological assessment of prostatic biopsies allowed us to define PCa subgroups based on GS grading into: -PA- (PCa of low risk; GS6 (3 + 3); *n* = 4), -PB- (intermediate risk PCa; GS7 (3 + 4); *n* = 4) and -PC- (high risk PCa; GS7 (4 + 3) or GS8 (4 + 4); *n* = 4).

After biotype assignment and considering the number of counts, tsRNAs and miRNAs were abundantly detected in semen sEV samples, as previously described [5]. After filtering out low-count features, differential expression analysis was conducted using DESeq, and *p*-values were adjusted for multiple comparisons using the false discovery rate (FDR) method. Initially, a filtering threshold of adjusted *p*-value < 0.05 and an absolute fold change |FC| > 1.2 was applied, which resulted in no differentially expressed (DE) sncRNAs. To reduce stringency and increase sensitivity, we subsequently modified the criteria by adopting a less conservative statistical threshold (*p*-value < 0.005) while simultaneously increasing the absolute fold change cutoff to |FC| > 1.5. This approach allowed us to identify a set of DE sncRNAs while maintaining a balance between statistical significance and biological relevance.

**Figure 1:**
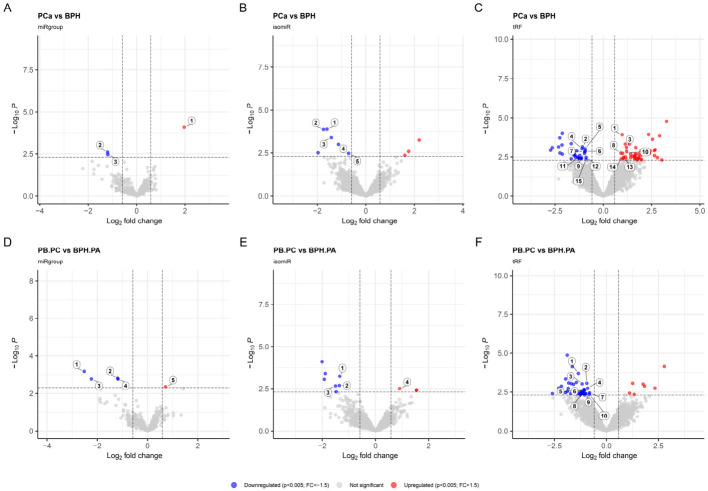
Volcano plots showing differentially expressed (DE) miRgroups, isomiRs and tRF isoforms in PCa vs. BPH (**A**–**C**) and PB.PC vs. BPH.PA (**D**–**F**) comparisons. Nominal *p*-value cutoff: 0.005. Enriched numbers: consecutive rank by *p*-value among preselected targets (1 = most significant). (**A**) PCa vs. BPH DE miRgroups: 1: hsa-miR-582-3p; 2: hsa-miR-187-3p; 3: hsa-miR-181a-2-3p. (**D**) PB.PC vs. PA.BPH DE miRgroups: 1: hsa-miR-122-5p; 2: hsa-miR-940; 3: hsa-miR-520a-5p; 4: hsa-miR-187-3p; 5: hsa-miR-504-5p. (**B**,**C**,**E**,**F**) See Table 2 and Table 3 for full isomiR/tsRNA feature names corresponding to each number in the volcano plot.

The altered expression patterns of sncRNAs are summarised in Figure 1.

Specifically, miRNA analysis identified 491 miRgroups (i.e., groups of miRNA sequence variants, including the canonical miRNA reference sequence annotated in databases as well as trimmed and extended variants, originating from a single miRNA gene), most of them (*n* = 480) showing >10 counts in all samples from at least one experimental group. Total expression of three miRgroups statistically differed (|FC| > 1.5; *p*-value < 0.005) between BPH and PCa samples (hsa-miR-181a-2-3p, hsa-miR-187-3p, hsa-miR-582-3p), with the first two being the most abundant (>100 counts), whereas five miRgroups differed between BPH.PA and clinically significant PB.PC individuals (hsa-miR-122-5p, hsa-miR-187-3p, hsa-miR-504-5p, hsa-miR-520a-5p, hsa-miR-940), with the first two being the most abundant (>100 counts). Some of the PCa patients (*n* = 3) included in the sRNAseq study presented chemical recurrence (increasing PSA levels) after surgery. When PCa samples were regrouped into recurrent (R) and non-recurrent (nonR) PCa, one DE miRgroup was identified (hsa-miR-494-3p).

A more detailed analysis of specific isomiR sequences showed 2062 isomiRs exhibiting >10 counts in all samples from at least one sample group, with the 3’isomiRs being the most prevalent isomiR category (74%) compared to the canonical (‘NA’ variant) (14.5%), the 5’isomiRs (7%) and the polymorphic isomiRs (4.5%), similar to what we found in infertile patients [5]. A closer look at the data showed that, first, regarding the BPH/PCa comparison, the levels of 12 isomiRs (belonging to 11 independent miRgroups) were statistically different (|FC| > 1.5; *p*-value < 0.005) between groups (Table 2A); 11 of them were able to discriminate BPH from PCa samples (AUC > 0.85; *p*-value < 0.05), and five of these isomiRs were more abundant, presenting >50 counts in both groups of the study, and were thus good candidates for further validation. The expression of these five DE isomiRs was diminished in PCa compared with BPH samples and, interestingly, two of these isomiR sequences corresponded to canonical miRNAs (hsa-miR-181a-2-3p, hsa-miR-193a-5p). Secondly, the expression of sixteen isomiRs was significantly different between BPH.PA and clinically significant PB.PC individuals (Table 2B); among those, four isomiR sequences exhibiting >50 counts in any experimental group were selected as candidates for validation in a larger cohort of samples. Three of the variants belonged to the hsa-miR-187-3p miRgroup (one of them corresponded to canonical miRNA), and one isomiR variant of hsa-miR-107 could discriminate between both prognosis groups (AUC: 0.92; *p*-value: 0.005). Finally, when R and nonR PCa were compared, we obtained seven DE isomiRs (Table 2C), three of them showing >50 counts in at least one of the groups of the study and one of them showing discriminatory potential (AUC:1; *p*-value: 0.013).

Interestingly, DE isomiR sequences were predominantly 3’isomiRs; some of them were expressed at higher levels than their respective canonical counterparts, whereas others represented a minor variant among those from the same miRgroup (Supplementary Table S2).

Regarding tsRNA evaluation, in total, 8403 tsRNAs were identified; 3962 of them presented >10 counts in all samples from at least one group. Among them, 84 tsRNAs were differentially expressed (|FC| > 1.5; *p*-value < 0.005) between semen sEV samples from malignant and benign prostate disease (Table 3A). Forty DE tsRNAs (15 overexpressed and 25 underexpressed in PCa) showed >50 counts in any of the groups of the study. These DE tsRNAs could be classified into: 3’ or 5’-tRNA halves (*n* = 6), 3′-tRNA-derived RNA fragments (3′tRFs) (*n* = 17), 5′tRFs (*n* = 4) and i-tRFs (*n* = 13). Twenty-one of them presented >50 counts in both groups of the study and at least 150 in one of the groups, with the most represented being DE tsRNAs. Among these 21, 15 tsRNAs were able to discriminate (AUC > 0.850) PCa from BPH samples (candidates for RT-qPCR validation).

When clinically significant PCa (PB.PC) was compared with low-risk prostate disease (BPH.PA), 66 DE tsRNAs were found. Only 26 of them (24 underexpressed and two overexpressed in PB.PC) showed >50 counts in any of the groups of the study, with half of them being classified as 3′tRFs (Table 3B). Among these 26, 10 tsRNAs presented at least 150 counts in one of the groups and were able to discriminate (AUC > 0.800) between groups (candidates for further validation).

From the 44 DE tsRNAs that resulted in the R-PCa/nonR-PCa comparison, seventeen of them, mostly 3′tRFs, showed >50 counts in one of the groups. Ten of them presented at least 150 counts in one of the groups, although they did not have discriminatory potential (Table 3C).

Overall, most of the identified differences were attributable to sncRNAs with low expression levels. Notably, clustering analysis indicated that preselected DE semen EV isomiRs/tsRNAs can potentially discriminate among groups (Supplementary Figure S1).

### 2.3. sncRNA Validation as Candidate Biomarkers

As we previously demonstrated [7], isomiR/tsRNA variability must be considered when designing RT-qPCR assays. DE sncRNAs from sRNAseq were filtered by several criteria. Concerning miRNA/isomiRs, the expression of each preselected candidate isomiR was compared with other variants from the same miRgroup (Supplementary Table S2). We retained only those isomiRs that were either most abundantly represented or that showed expression trends similar to the most abundant DE variants within the same miRgroup, paying special attention to 3′-end isoforms. Thus, from the five preselected isomiRs for the BPH/PCa comparison, two canonical miRNAs (miR-181a-2-3p&NA&iso-22-05X17UNLJ, miR-193a-5p&NA&iso-22-93NYMJ5EL) and one isomiR variant (miR-187-3p&iso_add3p:1&iso-23-W4WY9YJ1DZ) were finally selected. From the four preselected isomiRs for the BPH.PA/PB.PC comparison, two isomiRs (miR-187-3p&NA&iso-22-W4WY9YJ14 canonical sequence and miR-187-3p&iso_add3p:1&iso-23-W4WY9YJ1DZ) were selected for primer design.

Regarding tsRNAs, expression of variants sharing the candidate tsRNA sequence was considered (including 3′-end isoforms differing by 1–2 nucleotides in length) (Supplementary Table S3); those tsRNAs that were not the predominant form (defined as having isoforms with at least a 10-fold higher expression than the tsRNA sequence index and/or with a high number of isoforms) were discarded. Thus, one tsRNA for the BPH/PCa comparison (Val-CAC&3′-tRF&tRF-34-D3KS7SB1RHODE2) and six tsRNAs for the BPH.PA/PB.PC comparison (Asn-GTT&3′-half&tRF-41-YDLBRY73WEK5KKOVD, Asp-GTC&3′-half&tRF-41-8HM2OSRN2NKSEK51B, Asp-GTC&3′-half&tRF-41-8HM2OSRNLNKSEK51B, Asp-GTC&3′-tRF&tRF-45-3W2VR008R959KUMKF6, Asp-GTC&3′-tRF&tRF-45-3W2VR008R9D9KUMKF6, Glu-TTC&3′-tRF&tRF-45-7Z2R1HPSR9O9337KB6) were chosen to be validated by RT-qPCR assays.

First, melting curve assessment of miRPrimer2 RT-qPCR experiments showed a single peak for every isomiR tested; however, a high Cq value (35–36) was obtained for miR-181a-2-3p (Table 4), suggesting low expression; thus, this variant was discarded for further analysis. With regards to tsRNA sequences, several melting curves were observed when Asn-GTT&3′half& tRF-41-YDLBRY73WEK5KKOVD was amplified (Table 4); thus, it was not considered further.

**Figure 2:**
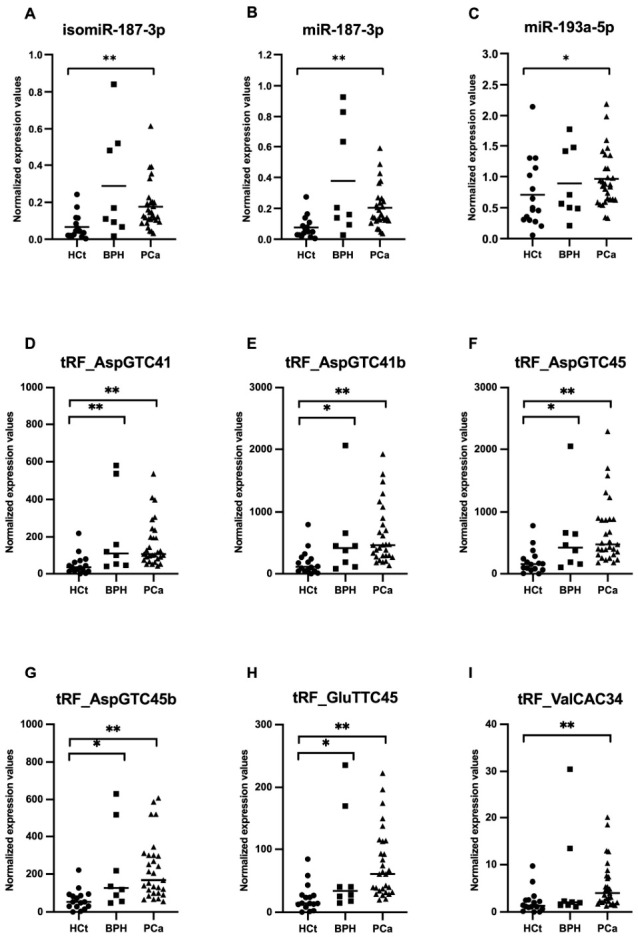
Semen sEV isomiR/tRF isoform levels in benign prostate hyperplasia (BPH) and malignant prostate tumour (PCa) compared with healthy controls (HCts). Expression profiling of isomiR-187-3p (hsa-miR-187-3p&iso_add3p:1&iso-23-W4WY9YJ1DZ) (**A**), miR-187-3p (hsa-miR-187-3p&NA&iso-22-W4WY9YJ14) (**B**) and miR-193a-5p (hsa-miR-193a-5p&NA&iso-22-93NYMJ5EL) (**C**) isomiR variants and tRF_AspGTC41 (tRF-41-8HM2OSRN2NKSEK51B) (**D**), tRF_AspGTC41b (tRF-41-8HM2OSRNLNKSEK51B) (**E**), tRF_AspGTC45 (tRF-45-3W2VR008R959KUMKF6) (**F**), tRF_AspGTC45b (tRF-45-3W2VR008R9D9KUMKF6) (**G**), tRF_GluTTC45 (tRF-45-7Z2R1HPSR9O9337KB6) (**H**) and tRF_ValCAC34 (tRF-34-D3KS7SB1RHODE2) (**I**) tRF isoforms in seminal small extracellular vesicles (sEVs) obtained by miRPrimer2 reverse transcriptase quantitative real-time polymerase chain reaction (RT-qPCR) quantification. Data are shown as relative quantification (RQ) values, which were calculated using the 2dCq strategy and relative to the expression values of miR-30e-3p. HCt: healthy control group (depicted as circles); BPH: benign prostatic hyperplasia (depicted as squares); PCa: prostate cancer (depicted as triangles). The horizontal bar displays the median expression value. Significant differences between groups are indicated: * *p*-value < 0.05; ** *p*-value < 0.01 (Mann–Whitney U-test). IsomiR variants are designated using the format miRNA&variant&UID, whereas tRFs are named according to the UID.

Finally, the results of RT-qPCR experiments on an expanded cohort of samples [including HCt individuals (*n* = 16), BPH (*n* = 8) and PCa (*n* = 29)] indicated that the expression patterns of the three selected isomiRs and the six tsRNAs (Figure 2) were differentially expressed between HCt and PCa patients but, surprisingly, not between BPH and PCa individuals. In line with that, classifying samples by biopsy GS severity revealed no differences in expression of individual isomiRs/tsRNAs between (BPH+PCa GS6) and (PCa GS7+GS8), although differences were found when HCts were included in the non-malignant group (HCt+BPH+PCa GS6) (Supplementary Figure S2).

**Figure 3:**
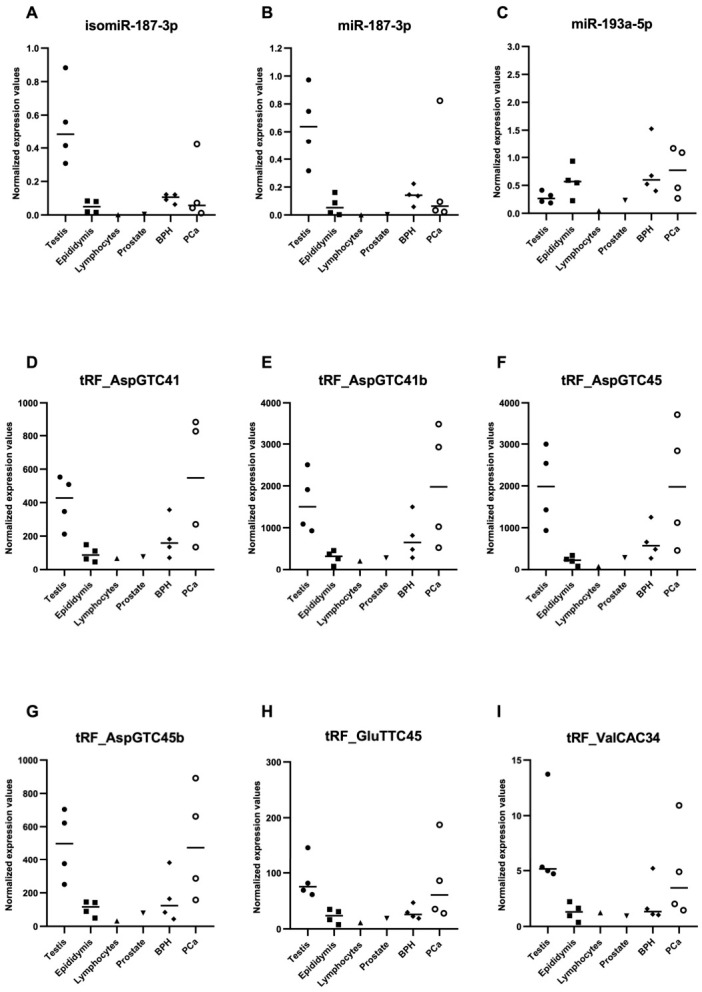
Tissue expression behaviour of 3 selected isomiRs and 6 selected tsRNAs. Expression profiling of isomiR-187-3p (hsa-miR-187-3p&iso_add3p:1&iso-23-W4WY9YJ1DZ) (**A**), miR-187-3p (hsa-miR-187-3p&NA&iso-22-W4WY9YJ14) (**B**) and miR-193a-5p (hsa-miR-193a-5p&NA&iso-22-93NYMJ5EL) (**C**) isomiR variants and tRF_AspGTC41 (tRF-41-8HM2OSRN2NKSEK51B) (**D**), tRF_AspGTC41b (tRF-41-8HM2OSRNLNKSEK51B) (**E**), tRF_AspGTC45 (tRF-45-3W2VR008R959KUMKF6) (**F**), tRF_AspGTC45b (tRF-45-3W2VR008R9D9KUMKF6) (**G**), tRF_GluTTC45 (tRF-45-7Z2R1HPSR9O9337KB6) (**H**) and tRF_ValCAC34 (tRF-34-D3KS7SB1RHODE2) (**I**) tRF isoforms, as determined by RT-qPCR in several reproductive organs such as the testis, epididymis and prostate. Controls of pathological prostate (benign prostate hyperplasia -BPH- and prostate cancer -PCa- prostate) are also included. Expression levels relative to miR-30e-3p are shown. Testis (depicted as circles); epididymis (depicted as squares); lymphocytes (depicted as inverted triangles); BPH: benign prostatic hyperplasia (depicted as diamonds); PCa: prostate cancer (depicted as open circles). The horizontal bar displays the median expression value. IsomiR variants are designated using the format miRNA&variant&UID, whereas tRFs are named according to the UID.

The assessment of the expression behaviour of these miRNA/tsRNA isoforms across the tissues of the reproductive tract showed that these sncRNA variants are not exclusively expressed in prostatic tissue and confirmed that these sncRNAs exhibit altered expression patterns in the BPH and PCa samples compared with the healthy prostate (Figure 3). 

To assess whether a multi-biomarker model improves performance over single biomarkers, a multivariate logistic regression analysis was applied. Two models, one including two isomiRs (miR-187-3p, miR-193a-5p) (AUC: 0.884; *p*-value: 0.001; Sn: 96.6%; Sp: 25%) [Table 5(1.B)] and another including four tRFs (AspGTC41, AspGTC45, GluTTC45, ValCAC34) (AUC: 0.884; *p*-value: 0.001; Sn: 100%; Sp: 62.5%) [Table 5(2.B)], were obtained, showing improved accuracy for discriminating PCa from BPH compared to PSA testing. Interestingly, these models not only exceed the discriminatory capacity of PSA but also the NPV: from 0% (when using PSA) to 66% (miR-based model) and 100% (tsRNA-based model).

When the PCa samples were classified by their severity or degree of PCa affectation according to the biopsy Gleason score (GS) or AJCC prognostic risk groups, the combined tsRNA model was able to discriminate (BPH+PCa_GS6) from (PCa_GS7+GS8) patients (AUC: 0.729; *p*-value: 0.018) [Table 5(2.D)]. Additionally, the combination of PSA values with tsRNA expression levels was able to improve classification of samples with intermediate PSA levels into prostate disease (BPH+GS6) or (PCa GS7+GS8) (AUC: 0.774; *p*-value: 0.005) [Table 5(2.D)] as well as into prostate disease with better (BPH+PCa_I) and worse prognosis (PCa IIA+IIB+IIC+IIIB) (AUC: 0.729; *p*-value: 0.017) [Table 5(2.E)].

### 2.4. Prediction of sncRNA Target Genes

**Figure 4:**
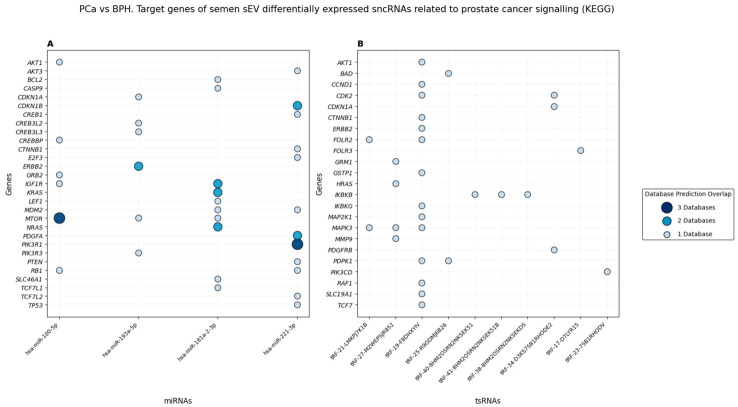
Bubble plots of predicted sncRNA–gene interactions within the KEGG prostate cancer pathway. Predicted targets of differentially expressed (**A**) miRNAs and (**B**) tsRNAs identified in semen sEVs. The *x*-axis shows the differentially expressed sncRNAs, while predicted target genes are displayed on the *y*-axis. Bubble size and colour intensity reflect the number of target prediction databases supporting each interaction (1, 2 or 3 databases). Interactions were derived from the target prediction results reported in Supplementary Tables S4 and S5. Graphical visualisation was prepared with the assistance of the Gemini AI tool.

Characterising the target genes of putative sncRNA biomarkers is important for understanding their contribution to disease aetiology. First, the application of the five preselected isomiRs from the BPH/PCa comparison on the miRNet web-based platform in silico analysis resulted in a list of 786 genes involved in 161 KEGG pathways; 42 of them resulted in an adj *p*-value < 0.05. Interestingly, “prostate cancer signalling” ranked among the first three positions of canonical pathways, after “pathways in cancer” and “cell cycle”, consisting of 19 deregulated target genes. For the application of the TargetScan and miRDB platforms, cutoff values of “TargetScan cumulative context++ score” <−0.15 and “miRdb score” >70, respectively, were applied. The total number of target genes obtained from the miRDB platform was 508 and 1927 in the TargetScan platform. In this regard, some concordant results were obtained among the different platforms (miRNet, TargetScan and miRdb) used, representing 5-18% of the predicted target genes. This was also observed in the genes involved in the “PCa signalling pathway” (Figure 4A and Supplementary Table S4A).

When the DE miRNAs from the BPH.PA/PB.PC comparison were included in the miRNet analysis, and genes involved in 180 KEGG pathways were obtained, with “pathways in cancer” being among the first three positions. In addition, the total number of target genes obtained from the miRDB platform was 458, and 149 were obtained from the TargetScan platform.

Additionally, fifteen DE tsRNAs from the BPH/PCa comparison were applied to tRFtar and sRNAtools [which use RNAhybrid (cutoff values: ΔG ≤ −22) and miRanda (cutoff values: score > 160; ΔG ≤ −20) platforms], resulting in a list of 528, 2887 and 2016 genes, respectively, some of them described in the “PCa signalling pathway” (KEGG) (Supplementary Table S5). The overlap in predicted target genes among tRF prediction platforms was limited, representing only 0.5–3.5% of the total predictions, and notably, no concordance was observed for genes within the “PCa signalling pathway” (Figure 4B).

When the ten DE tsRNAs from the BPH.PA/PB.PC comparison were analysed, 29 target genes were obtained in the tRFar platform, 0 target genes were obtained in the RNAhybrid platform, and 5972 target genes were obtained in the miRanda platform.

## 3. Discussion

Despite significant advances in PCa clinical diagnosis, accurately distinguishing malignant from benign prostate conditions using non-invasive methods remains a major clinical challenge, particularly for patients with PSA levels in the 4–10 ng/mL “grey zone”.

Over the last decade, minimally invasive approaches based on liquid biopsies have gained attention for PCa diagnosis and prognosis. Circulating RNAs within sEVs have emerged as promising biomarkers accessible through liquid biopsies (blood, urine and semen). Although current commercially available non-invasive tests use urine, semen is a biologically relevant alternative, enriched in prostate-derived sEVs due to its high prostate secretion content (~70%); therefore, prostate-derived sEV biomarkers are expected to be more abundant in semen than in serum or urine, with the potential of reflecting underlying tumour biology even at early stages of the disease. Accurate detection of EV-contained sncRNA variants requires high-resolution protocols such as high-throughput sequencing, which enable detailed sncRNA profiling and improved biomarker selection for downstream clinical assays such as RT-qPCR. Here, after sRNAseq characterisation, we present potential non-invasive, sensitive and specific biomarker panels based on semen sEV sncRNAs, which provide accurate information on prostate disease malignancy beyond routine PSA testing in patients within the PSA grey zone.

The robust sRNAseq assessment of sncRNA abundance in semen sEVs from benign and malignant prostate disease revealed limited differences between groups, in line with our previous RT-qPCR array study [6]. Notably, many of the DE sncRNAs exhibited low expression levels, which may limit their robustness as biomarkers and complicate validation efforts. The current sRNAseq analysis revealed a moderate number of DE sncRNAs including 12 and 17 isomiRs in the BPH/PCa and BPH.PA/PB.PC comparisons respectively. A substantially higher number of tsRNAs were detected with 84 (BPH/PCa comparison) and 66 (BPH.PA/PB.PC comparison) tsRNAs identified. Most expression differences involved tsRNAs, which is not an unexpected finding since the epididymis and prostate are the main contributors of sEV tsRNAs/tRFs in the male reproductive tract [8].

The modest number of discriminatory semen EV sncRNAs likely aligns with the intra-tumoral heterogeneity, observed by ISH in PCa tissue [9], which could also explain why, although differences in sncRNA expression between BPH and PCa tissue are observed, they could not be consistently established. Previous publications on PCa tissue showed profiles of isomiRs and tRFs in malignant tissue compared with prostatic tissue from healthy controls [10], as well as dysregulated 5′tRFs and 3′tRFs in PCa tissue when compared to adjacent normal tissue [11,12]. Biological evidence can further provide an additional explanation. BPH and PCa share androgen-dependent growth [13], chronic inflammation [14] and specific microenvironmental changes; thus, shared underlying molecular mechanisms are likely to occur. Additionally, as miRNA/tRF expression is associated with GS, the predominance of GS 6/7 (3 + 4), mostly low-grade tumours, in our cohort may further explain the modest differences observed relative to BPH.

Given the complex pathophysiological process and heterogeneous clinical phenotypes, multivariate models are more adequate to accurately detect and monitor patient health outcomes. Although no single marker was informative enough in this scenario, as no isomiRs or tsRNAs were DE between BPH and PCa individuals in the RT-qPCR validation step, multivariate models are still useful as PCa biomarkers, providing higher accuracy and increasing the NPV of the PSA variable used in the clinical screening process. Two miRNA/tsRNA classifiers in seminal sEVs were developed, one model including two isomiRs (miR-187-3p+miR-193a-5p) and the other including four tRFs (AspGTC41+AspGTC45+GluTTC45+ValCAC34), useful as non-invasive biomarkers. Combined isomiR or tRF models contribute to improving PCa diagnosis in men within the PSA grey zone, while tRF-based models, when combined with PSA levels, also appear to be effective for the prognosis of PCa while reducing unnecessary biopsies.

These results corroborate our previous RT-qPCR study assessing four 5′tRFs in semen sEVs, selected from the 5′tRF profile in PCa tissue compared with the adjacent non-tumoral tissue. While 5′tRF dysregulation was observed in semen sEV PCa relative to HCts, no differences were detected when compared with BPH. Multivariate models helped overcome this limitation [15].

The biologically relevant miRNAs identified in this study have been previously implicated in PCa pathogenesis. miR-187-3p distinguishes non-malignant from malignant prostate biopsies [16], showing a similar expression pattern to our semen EV findings. miR-187-3p is significantly downregulated in PCa compared with BPH tissues, acting as a tumour suppressor. Additionally, hsa-miR-187-3p expression is inversely correlated with GS [17], is poorly expressed in metastatic PCa tissue and can target androgen-regulated oncogene ALDH1A3 in PCa [18]. EV-associated miR-187 contributes to repressing cell malignant features and tumour metastasis [19].

miR-193a-5p is upregulated in human PCa tissues [20] as well as in PCa (LNCaP, PC3 and DU145) compared with normal prostate epithelial (RWPE-1) cell lines. miR-193a-5p inhibits oxidative stress-induced apoptosis in PC3 cells [21].

Unexpectedly, none of the DE canonical miRNAs previously identified between PCa and BPH by RT-qPCR arrays [6] were identified as DE in the current sRNAseq analysis. Similarly, none of the 5′tRFs previously evaluated in semen sEVs by RT-qPCR analysis were found to be differentially expressed in the current sRNA-seq dataset [15]. This discrepancy likely reflects methodological and statistical differences, including the application of stricter significance thresholds in RNA seq (*p*-value < 0.005 vs. *p*-value < 0.05 in the RT-qPCR array) and the poor representation of some (14/27) RT qPCR-identified DE miRNAs that were not detected by sequencing. This mismatch may partially arise from cross-reactivity among closely related sequences in RT-qPCR assays; consequently, what is often reported as the expression level of a single miRNA/tsRNA may actually reflect the combined abundance of multiple closely related variants, underscoring the relevance of considering isomiR/tsRNA variability when designing and interpreting RT-qPCR analysis. Conversely, RNA seq resolved distinct isomiR variants that RT qPCR assays cannot reliably discriminate, potentially explaining part of the observed differences.

Prostate cancer is often multifocal, and even extended mpMRI targeted biopsy protocols can miss a proportion of tumours [22]. Therefore, detecting cancer through fluid-based biomarkers is highly desirable, offering advantages over tissue biopsy. Such biomarkers can support clinical decision-making, helping to determine whether to continue conservative follow-up using non-invasive approaches, such as serial PSA measurements and multiparametric MRI examinations, or to perform a new biopsy is more appropriate, particularly when PSA levels are moderately elevated, allowing for non-invasive monitoring of potentially low-grade disease.

In conclusion, several sncRNA profiles have been proposed as promising diagnostic/prognostic biomarkers for PCa in semen sEVs of patients with moderately altered PSA levels. These findings underscore the potential of semen sEV-associated sncRNAs as minimally invasive tools for PCa screening, warranting further investigation to establish their clinical applicability and specificity. This study is limited by its relatively small sample size, which may undermine the reliability of our results. Consequently, validation in larger cohorts is warranted to confirm the observed associations and assess the clinical utility of the identified sncRNA signatures.

## 4. Materials and Methods

### 4.1. Subjects of Study

Semen samples were obtained from 16 healthy individuals consulting for vasectomy at Fundació Puigvert (control group 1: HCt), including pre-vasectomy (HCt-noV) and post-vasectomy (HCt-V) donors. In addition, 37 individuals who consulted for PCa diagnosis at Bellvitge Hospital were included, all with moderate PSA levels (4–18 ng/mL), who consented to undergo prostate biopsy. This cohort consisted of 29 men with biopsy-confirmed PCa, including vasectomised [PCa-V] and non-vasectomised individuals [PCa-noV], as well as 8 non-vasectomised individuals diagnosed with BPH [control group 2] who exhibited elevated PSA levels (>4 ng/mL) but no detectable cancer on biopsy (Table 1; Supplementary Table S1). We also stratified samples according to AJCC 8th edition prognostic risk groups [23] (which consider TNM data, pre-treatment PSA levels, and tumour Gleason grade) into I (low risk, including organ confined GS6 samples and PSA < 10ng/mL), IIA (low–intermediate risk, which includes organ confined GS6 samples and PSA > 10ng/mL), IIB (intermediate risk, including organ confined GS7 (3 + 4) samples), IIC (intermediate–high risk, which includes organ confined GS7 (4 + 3) samples), and IIIB (high risk advanced due to extra-prostatic extension of the tumour, which includes cT3 samples belonging to any GS grade group and PSA levels).

The study was approved by the Ethical Review Board of both institutions. The participants signed an informed consent form. All methods were performed in accordance with the relevant guidelines and regulations.

### 4.2. Semen Samples and sEV Isolation

Semen samples were obtained by masturbation after 3–5 days of sexual abstinence and incubated at 37 °C for 30 min to allow liquefaction. Seminal sEVs were isolated by differential centrifugation steps, including a microfiltration–ultracentrifugation procedure, and were characterised by nanoparticle tracking analysis, as described elsewhere [6,24].

### 4.3. Small RNA-Containing Total RNA Isolation

#### 4.3.1. From Semen sEVs

Total RNA was isolated from sEVs using an miRNeasy Micro Kit (Qiagen, Venlo, Limburg, The Netherlands) as previously described [15,25]. RNA yield was quantified using a QUBIT fluorometer and a Quant-iT RNA Assay kit (Invitrogen; Thermo Fisher Scientific; Waltham, MA, USA). All RNA samples exhibited OD 260/280 nm ratios of 1.7 or higher when using a Nanodrop UV-Vis spectrophotometer (Thermo Fisher Scientific).

#### 4.3.2. From Tissue

Small RNA-containing total RNA was obtained with a mirVana miRNA Isolation Kit (Ambion; Thermo Fisher Scientific) from frozen biopsies (−80 °C) from different organs of the reproductive tract. Specifically, testicular biopsies (*n* = 4) were obtained from infertile men with obstructive azoospermia (with a conserved spermatogenic pattern). Post-mortem epididymis (*n* = 4) and prostate (*n* = 1) samples from healthy donors, as well as prostate tissue from PCa (*n* = 4) and BPH (*n* = 4) patients, were kindly provided by the Pathological Anatomy Service of the Bellvitge Hospital.

RNA quality was assessed with an Agilent 2100 Bioanalyzer (Agilent Technologies, Waldbronn, Germany). Tissular RNA samples included in the study had a 28S/18S ratio > 1.4 and a RIN value > 7.5.

### 4.4. Small RNA Sequencing

Small RNA libraries were generated with the NextFlex Small RNA-Seq Kit v4 with Unique Dual Indices—UDIs—(Revvity, Waltham, MA, USA) adapted to lower input (10 ng total RNA from sEVs, with 18 PCR amplification cycles, according to specifications), excluding the final bead clean-up to allow for an extended range of gel-based automated size selection. An equimolar pool of all samples determined by Bioanalyzer was used as input. QIAquick (Qiagen) column purification was performed before and after separation with the Pippin Prep system (SAGE Science, Beverly, MA USA) on 3% dye-free cassettes with DF marker F, using two different settings in range mode [start 135 bp, end 185 bp, and target 160 bp for the first fraction (automated elution), and then 8 more fractions were collected every 4 min (manually timed elution)]. The first three fractions recovered, which contained most of the small RNA, were pooled by equal mass, estimated from Bioanalyzer and run on a NovaSeq 6000 system (Illumina, San Diego, CA, USA) SP 200 cycle flow cell to generate paired-end reads (2 × 100 nt). Data is available in the GEO database (accession number: GSE330457).

### 4.5. sRNAseq Data Analysis

sRNAseq data was analysed with the XICRA (version 1.2.4, https://github.com/HCGB-IGTP/XICRA) pipeline and XICRA.stats (version 0.2-1, https://github.com/HCGB-IGTP/XICRA.stats), the R companion package (accessed on 10 February 2025) [26]. See additional details at https://xicra.readthedocs.io/en/latest/ (accessed on 10 February 2025).

Briefly, we checked raw data quality using FASTQC (version 0.11.4 http://www.bioinformatics.babraham.ac.uk/projects/fastqc/) (accessed on 10 February 2025) before and after trimming the reads to discard sequencing adapters with cutadapt (version 2.10) [27]. For the mapping and the biotype feature assignment of all reads, we used Bowtie (version 1.3.0) mapping [28] software with parameters specifically intended for small RNAs (-v 1–best–strata). Reads were mapped to the human genome (GRCh38.p13 2022), and featureCounts (version 1.5.1) [29] was used to assign counts based on the human genome (hg38) and RNA central annotations [30].

For miRNA identification, we used miraligner (version 3.5, within SeqBuster software, which resolves canonical and other isomiR variants [31], and miRBase [32] (v22) as the reference database. Results were standardised using miRTop (version 0.4.23) [33], which also generated expression count matrices. MiRNA expression at the gene level was obtained by aggregating isomiR counts for each miRNA. For tRNA and piRNA biotypes, we used two different approaches: (i) analysis using all reads and (ii) a sequential approach in which only reads unaligned in previous steps were processed. In the sequential strategy, reads were mapped with the following scheme: miRNA > tsRNAs > piRNA > other biotypes (human). For the tRNA/tsRNA analysis, we used both MINTmap (version 1.0) [34] (querying the MINTbase MINTmap_v1 pre-compiled look up table set implemented in XICRA) and miRge [35] (version 3.0, with bowtie -v 0 -k 1 --best --strataLogic:-v 0 for Tier 1 (mature miRNA), bowtie -v 1 -m 100 --best --strataLogic:-v 1 for Tier 2 (Hairpin, tRFs, snoRNA, and rRNA), and bowtie -v 1 -m 50 --best –strata for Tier 3 (human genome, mRNA). 

Finally, for the analysis of DE small RNAs, we employed XICRA.stats, running DESeq2 (version 1.46.0) [36,37] and related packages in the R environment (R version 4.4.0). For each case, the optimal dispersion fit (local or parametric) was selected. Due to the number of expression counts in miRNA and tsRNA analysis, differential expression between groups was estimated using apeglm (Approximate Posterior Estimation for the Generalised Linear Model) [37], which reduces the number of false positive DE genes by filtering by log2 fold change (LFC) while preserving true positive DE genes. piRNA analysis was not further performed because of the presence of many clusters with low counts and/or the total presence/absence of counts.

Qualitative analysis was done based on raw counts. A given small RNA was accepted as present when the raw count was at least 10 copies in all samples of at least one group per comparison.

We compared results obtained with two linear models in DESeq2: a simple model and a complex model including age as a covariate.

### 4.6. Validation of sncRNA Candidates by RT-qPCR Analysis

We used the miRPrimer2 strategy, similar to that previously stated [24].

Reverse transcription of 40 ng of semen sEV total RNA in a final volume of 10 μL in the presence of ATP, RT primer (50-CAGGTCCAGTTTTTTTTTTTTTTTVN, where V is A, C and G and N is A, C, G and T), poly(A) polymerase, dNTPs, and Superscript IV enzyme (200 U/μL) (Invitrogen, ThermoFisher Scientific) at 42 ◦C for 1 h. We found that tsRNA sequences are better amplified than miRNA sequences; thus, in the current study, cDNA samples were diluted (8×) for miRNA quantification and (400×) for tsRNA quantification, and 10 μL PCR reactions were performed (in duplicates) with 1 μL of diluted cDNA, 5 μL of 2× SYBR Green mix (Roche; Basilea, Switzerland), and 250 nM of forward and reverse primers designed by miRprimer2 software version 2.0 (https://zenodo.org/record/1339289#.Ymj5i_exVGE (accessed on 4 October 2025)) [38].

Cycling conditions were set up as follows: 5 min at 95 ◦C; 40 cycles of 10 s at 95, followed by 30 s at 60 ◦C; and finally, melting curve analysis from 60 ◦C to 99 ◦C. qPCR was also performed on a LightCycler^®^ 96 Instrument (Roche, Basilea, Switzerland). The expression value of hsa-miR-30e-3p was used for data normalisation, previously described to be among the most stable assays in PCa sEV semen samples [6]. Relative quantification (RQ) values were calculated using the 2dCp strategy. The sequences of primers are detailed in Table 2, including previously described primers for hsa-miR-30e-3p [7].

The same procedure was applied to determine cell expression profiling of tsRNA candidates with some modifications: 40 ng of tissular RNA was reverse-transcribed, and cDNA was diluted (60×) for miRNA quantification and (480×) for tRF quantification.

### 4.7. Statistical Analysis

Statistical analyses were carried out by using SPSS statistical software version 20 (SPSS, Chicago, IL, USA), and a *p*-value < 0.05 was considered statistically significant. Differences in sncRNA relative expression were assessed using the Mann–Whitney U-test, and correlations with clinical variables were evaluated by the Pearson method. Receiver operating characteristic (ROC) curve analysis [39] of the RQ values was used to assess the ability of each sncRNA tested to distinguish the samples showing prostate malignancy. Accuracy was measured as the area under the ROC curve (AUC) [39]. The threshold value was determined by Youden’s index, calculated as sensitivity plus specificity-1 [40]. A multivariate binary logistic regression analysis (backwards stepwise, conditional method) identified the optimal combination of variables associated with the presence of PCa or with the aggressiveness of the disease.

### 4.8. Determining in Silico Target Genes of sncRNAs

The miRNet web-based platform (https://www.mirnet.ca), applying default parameters [41] (accessed on 17 February 2026), was used to identify target genes and pathways potentially altered by the canonical miRNA signature. TargetScan (https://www.targetscan.org/vert_80/) (accessed on 17 February 2026) and miRDB (https://mirdb.org/) (accessed on 17 February 2026) were additionally used for comparison.

Target genes and pathways potentially affected by the tsRNA signature were initially identified using the tRFTar platform (http://www.rnanut.net/tRFTar/) (accessed on 20 March 2026) [42]. Since several tRFs function through miRNA-like mechanisms, additional target analyses were conducted using the miRNA target-prediction platform sRNAtools (https://rnainformatics.org.cn/sRNAtools/) (accessed on 20 March 2026) [43], which integrates sncRNA target detection algorithms, such as miRanda and RNAhybrid.

## Figures and Tables

**Table 1:** Clinical features of individuals included in the study.

Variable	HCt_noV	HCt_V	BPH	PCa_noV	PCa_V
Total (*n*)	5	11	8	22	7
Age, range (years)	37–44	32–65	52–64	50–72	43–68
Age, mean ± SD (years)	40 ± 3.08	39.82 ± 9.46	58.62 ± 4.50	59.04 ± 6.31	58 ± 9.69
**Pre-biopsy PSA**					
≤10 ng/mL (*n*)	nd	nd	8	20	4
>10 ng/mL (*n*)	nd	nd	0	2	3
Pre-biopsy PSA, mean ± SD (ng/mL)	nd	nd	6.18 ± 1.70	6.93 ± 3.00	8.96 ± 4.81
**Gleason score**					
Gleason score biopsy 6 (3 + 3) (*n*)	nd	nd	nd	10	3
Gleason score biopsy 7 (3 + 4) (*n*)	nd	nd	nd	7	4
Gleason score biopsy 7 (4 + 3) (*n*)	nd	nd	nd	4	0
Gleason score biopsy 8 (4 + 4) (*n*)	nd	nd	nd	1	0
**Clinical stage**					
T1c (*n*)	nd	nd	nd	15	2
T2a (*n*)	nd	nd	nd	0	1
T2c (*n*)	nd	nd	nd	5	3
T3a (*n*)	nd	nd	nd	2	1
Prognostic group *					
I (*n*)	nd	nd	nd	7	2
IIA (*n*)	nd	nd	nd	3	1
IIB (*n*)	nd	nd	nd	6	3
IIC (*n*)	nd	nd	nd	4	0
IIIB (*n*)	nd	nd	nd	2	1

HCt_noV: healthy control non-vasectomised individuals; HCt_V: healthy control vasectomised individuals; BPH: benign prostate hyperplasia group; PCa_noV: prostate cancer from non-vasectomised individuals; PCa_V: prostate cancer from vasectomised individuals; nd: not determined. * American Joint Committee on Cancer Prognostic Stage grouping (8th edition). PCa samples were further stratified according to AJCC 8th edition prognostic risk groups, which integrate TNM classification, pre-treatment serum PSA levels, and tumour Gleason score. Risk groups were defined as follows: stage I (low risk: organ-confined GS6 and PSA < 10 ng/mL), stage IIA (low–intermediate risk: organ-confined GS6 and PSA ≥ 10 ng/mL), stage IIB (intermediate risk: organ-confined GS7 (3 + 4)), stage IIC (intermediate–high risk: organ-confined GS7 (4 + 3)), and stage IIIB (high-risk advanced disease due to extra-prostatic extension, cT3 tumours of any Gleason grade group and PSA.

**Table 2:** List of differentially expressed (DE) isomiRs in small RNAseq.

**A. PCa vs. BPH**						
miRNA (*)	**Variant**	**UID**	**Count Mean BPH**	**Count Mean PCa**	***p*-Value**	**AUC (*p*-Value)**
**hsa-miR-100-5p (4)**	**iso-add3p:1**	**iso-23-BRS28UEY0N**	685.621	323.696	0.001	0.958 (0.008)
**hsa-miR-181a-2-3p (1)**	**NA**	**iso-22-05X17UNLJ**	145.437	57.259	0.000	0.896 (0.021)
hsa-miR-187-3p	iso-add3p:1	iso-22-W4WY9YJ1L	88.287	19.978	0.003	0.792 (0.09)
**hsa-miR-187-3p (3)**	**iso-add3p:1**	**iso-23-W4WY9YJ1DZ**	364.586	124.921	0.000	0.917 (0.015)
**hsa-miR-193a-5p (5)**	**NA**	**iso-22-93NYMJ5EL**	321.182	195.952	0.003	1 (0.004)
hsa-miR-20a-3p	NA	iso-22-EVIQ5FNOH	13.589	40.061	0.003	0.875 (0.029)
**hsa-miR-221-3p (2)**	**iso-add3p:1**	**iso-24-FXJYWV93K3**	180.426	55.455	0.003	0.938 (0.011)
hsa-miR-320a-3p	iso-5p:−1	iso-23-OBQ87U5KDL	22.997	8.785	0.005	0.875 (0.029)
hsa-miR-33a-3p	iso-3p:−1	iso-21-24ZR03PMB	0.516	9.699	0.000	0.958 (0.008)
hsa-miR-34a-5p	iso-3p:−1	iso-21-9F7MYJ9N0	8.447	24.132	0.004	0.875 (0.029)
hsa-miR-501-3p	iso-5p:+1	iso-21-IFK8P059E	28.054	7.558	0.004	0.854 (0.039)
hsa-miR-582-3p	NA	iso-22-UIRYBONBJ	4.042	18.021	0.001	0.958 (0.008)
**B. PB.PC vs. BPH.PA**						
miRNA (*)	**Variant**	**UID**	**Count Mean BPH.PA**	**Count Mean PB.PC**	***p*-Value**	**AUC (*p*-Value)**
hsa-let-7d-5p	iso-3p:−3	iso-19-FKVLRY1B	10.547	21.453	0.004	0.875 (0.012)
**hsa-miR-107 (4)**	**iso-3p:−3**	**iso-20-FPJYUP6X**	49.913	82.150	0.003	0.922 (0.005)
hsa-miR-10b-3p	iso-3p:−1	iso-21-02YUYXR2B	39.067	20.543	0.005	0.734 (0.115)
hsa-miR-130b-3p	iso-3p:−2	iso-20-J424IB1J	103.234	16.866	0.001	0.703 (0.172)
**hsa-miR-187-3p (2)**	**NA**	**iso-22-W4WY9YJ14**	16.947	7.926	0.004	0.766 (0.074)
**hsa-miR-187-3p (3)**	**iso-3p:+1**	**iso-23-W4WY9YJ1DX**	193.656	86.306	0.002	0.75 (0.093)
hsa-miR-187-3p	iso-add3p:1	iso-22-W4WY9YJ1N	231.350	98.276	0.002	0.719 (0.141)
**hsa-miR-187-3p (1)**	**iso-add3p:1**	**iso-23-W4WY9YJ1DZ**	41.335	18.990	0.000	0.75 (0.093)
hsa-miR-187-3p	iso-add3p:2	iso-24-W4WY9YJ12B	255.082	114.594	0.001	0.813 (0.36)
hsa-miR-34b-3p	iso-5p:+1	iso-21-D64D85X6E	27.687	10.571	0.000	0.766 (0.074)
hsa-miR-4492	iso-5p:+1	iso-16-RIRHLVD	17.876	4.664	0.000	0.773 (0.066)
hsa-miR-449a	iso-3p:−2	iso-20-9F7LZNFY	44.832	13.299	0.001	0.859 (0.016)
hsa-miR-505-3p	iso-3p:+2	iso-24-M60IXY79H3	30.849	9.490	0.003	0.758 (0.083)
hsa-miR-516a-5p	iso-3p:−2	iso-21-Y952DBPIE	9.448	24.575	0.004	0.859 (0.016)
hsa-miR-520a-5p	NA	iso-21-465KDLN9E	15.282	5.691	0.002	0.703 (0.172)
hsa-miR-873-5p	iso-3p:−3	iso-18-PKBZ7003	26.575	12.040	0.002	0.578 (0.6)
**C. R-PCa vs. nonR-PCa**						
**miRNA**	**Variant**	**UID**	**Count Mean nonR-PCa**	**Count Mean R-PCa**	***p*-Value**	**AUC (*p*-Value)**
hsa-let-7d-5p	iso-add3p:1	iso-22-FKVLRYJPO	43.141	20.933	0.002	0.852 (0.079)
hsa-miR-17-3p	iso-3p:−3	iso-19-EV1U1FHP	21.306	13.266	0.000	0.704 (0.309)
hsa-miR-18a-5p	iso-3p:−1	iso-22-UKXQ43P2O	15.880	2.194	0.003	1 (0.013)
hsa-miR-193b-3p	iso-add3p:3	iso-25-BYP7208S4N	7.684	23.890	0.003	1 (0.013)
hsa-miR-339-5p	iso-3p:−1	iso-22-87S7KKF91	19.458	53.113	0.001	1 (0.013)
hsa-miR-339-5p	iso-3p:−2	iso-21-87S7KKF9B	78.182	111.038	0.004	0.778 (0.166)
hsa-miR-487b-3p	iso-3p:−1	iso-21-DSUPR685E	94.171	137.352	0.004	0.63 (0.518)

DE isomiRs (|FC| > 1.5; *p*-value < 0.005 by DESeq). IsomiRs with more than 50 counts in any of the groups are depicted in blue. Among those, isomiRs selected for further validation are depicted in bold. Orange-shaded cells denote isomiRs with statistically significant differences between groups (*p*-value < 0.05 by AUC) and discriminative performance. * Numbers in parentheses correspond to the labelled points in Figure 1 (volcano plot). “NA” variant refers to the canonical miRNA sequence.

**Table 3:** List of differentially expressed (DE) tsRNAs in small RNAseq.

**A. PCa vs. BPH**						
tRNA (*)	**Variant**	**tsRNA ID (MINTbase)**	**BPH Mean Value**	**PCa Mean Value**	***p*-Value**	**AUC (*p*-Value)**
Ala-AGC	3′-tRF	tRF-19-5RKJH9FV	71.471	124.192	0.004	0.813 (0.069)
Ala-AGC	3′-tRF	tRF-20-LMKPJ7K1	44.724	101.294	0.000	0.896 (0.021)
**Ala-AGC (10)**	**3′-tRF**	**tRF-21-LMKPJ7K1B**	98.691	187.351	0.002	0.875 (0.029)
Ala-AGC	3′-tRF	tRF-21-LMKPU7K1B	3.246	16.460	0.001	
**Ala-AGC (13)**	**3′-tRF**	**tRF-27-M2WEPSJR852**	96.535	210.214	0.003	0.938 (0.011)
Ala-CGC	3′-tRF	tRF-21-L1K847K1B	8.229	26.184	0.004	
Ala-CGC	3′-tRF	tRF-25-R9OR3J8926	8.453	22.402	0.005	
Ala-TGC	3’-tRF	tRF-27-M3WEKSP1852	6.350	21.980	0.004	
Ala-TGC	i-tRF	tRF-17-DR3JH92	6.754	24.089	0.002	
Ala-TGC	i-tRF	tRF-22-R9DR3JH92	3.034	17.084	0.000	
Ala-TGC	i-tRF	tRF-34-VU6RM3WEKSPMJ2	4.770	33.124	0.001	
Arg-ACG	3’-tRF	tRF-30-59J3WD8YQ84V	14.569	39.269	0.002	
Arg-ACG	i-tRF	tRF-32-O2Y6R9O9NJ9IK	19.553	43.642	0.005	
Arg-CCG	3’-tRF	tRF-21-L3KQNKW1B	3.718	19.587	0.002	
Arg-CCT	3’-tRF	tRF-20-5MK19KU6	6.621	25.373	0.001	
Arg-CCT	3’-tRF	tRF-24-7S1R27R3I2	15.091	48.604	0.003	
Arg-CCT	3’-tRF	tRF-24-R95MK19K2V	4.841	22.508	0.000	
Arg-CCT	3’-tRF	tRF-26-RNL3KQNK71B	17.170	50.185	0.004	0.896 (0.021)
Arg-CCT	3’-tRF	tRF-29-987S1R2RR3E2	261.481	122.608	0.002	0.729 (0.182)
Arg-TCG	3′-tRF	tRF-20-LEKZM8Z1	5.319	20.774	0.003	
Arg-TCG	3′-tRF	tRF-23-YUHRYWRYD2	15.693	37.837	0.005	
Arg-TCG	3′-tRF	tRF-25-13W087W4SV	24.616	70.304	0.002	0.896 (0.021)
Asn-GTT	i-tRF	tRF-37-U1O0789NLJK1JK1	11.340	76.212	0.000	0.854 (0.039)
**Asp-GTC (9)**	**3′-half**	**tRF-39-8HM2OSRNLNKSEKH9**	9992.165	4829.004	0.002	0.875 (0.029)
**Asp-GTC (12)**	**3′-half**	**tRF-40-8HM2OSRN2NKSEK51**	2319.012	1187.143	0.003	0.854 (0.039)
**Asp-GTC (7)**	**3′-half**	**tRF-40-8HM2OSRNLNKSEK51**	16,385.958	8039.687	0.002	0.875 (0.029)
**Asp-GTC (5)**	**3′-half**	**tRF-41-8HM2OSRN2NKSEK51B**	12,374.005	6256.890	0.001	0.896 (0.021)
**Asp-GTC (6)**	**3′-half**	**tRF-41-8HM2OSRNLNKSEK51B**	63,718.590	32,892.542	0.001	0.917 (0.0159
**Asp-GTC (15)**	**i-tRF**	**tRF-37-8HM2OSRNLNKSEKL**	1778.098	974.248	0.005	0.958 (0.008)
**Asp-GTC (4)**	**i-tRF**	**tRF-38-8HM2OSRN2NKSEKDS**	1393.310	632.085	0.001	0.917 (0.015)
**Asp-GTC (2)**	**i-tRF**	**tRF-38-8HM2OSRNLNKSEKDS**	7570.516	3598.703	0.001	0.938 (0.011)
Asp-GTC	i-tRF	tRF-38-NMEH623K7SIR3DDY	58.494	13.524	0.000	0.958 (0.008)
Cys-GCA	5′-tRF	tRF-23-RKJP4P9LDS	14.907	2.813	0.001	
Glu-CTC	i-tRF	tRF-39-W631QJ3KYUYRR6IX	107.336	43.602	0.003	0.917 (0.015)
Glu-TTC	5′-tRF	tRF-29-86V8WPMN1EJ3	109.110	51.641	0.002	0.875 (0.029)
Glu-TTC	5′-tRF	tRF-39-86J8WPMN1E8Y7ZFV	603.362	322.308	0.004	0.813 (0.069)
Glu-TTC	5′-tRF	tRF-41-86J8WPMN1E8Y7Z2RB	392.127	180.753	0.001	0.792 (0.09)
Glu-TTC	i-tRF	tRF-19-1HPSR9HW	54.026	24.074	0.004	0.958 (0.008)
Glu-TTC	i-tRF	tRF-21-3HPSR959D	51.366	20.136	0.000	1 (0.004)
Glu-TTC	i-tRF	tRF-30-KKM1M3WD8S74	19.175	3.742	0.001	
Glu-TTC	i-tRF	tRF-31-KF9MVH7P59N3E	55.184	15.216	0.000	0.854 (0.039)
Glu-TTC_Phe-GAA	3′-tRF	tRF-20-LNK88KO1	6.456	23.778	0.005	
Gly-CCC	5′-tRF	tRF-38-PNR8YP9LON4V4R8	36.464	13.688	0.002	
Gly-CCC	5′-tRF	tRF-49-PNR8YP9LON4V4REH621K	94.450	38.994	0.002	0.958 (0.008)
Gly-CCC	i-tRF	tRF-48-PV94V4SMIFFNKQWYL1D4	117.345	26.507	0.002	0.604 (0.544)
Gly-CCC	i-tRF	tRF-49-LSN3S3RQJVDE86YZQD14	95.553	16.718	0.001	0.854 (0.039)
Gly-GCC	3′-tRF	tRF-20-LNK8KQP1	7.791	22.438	0.003	
Gly-GCC	i-tRF	tRF-37-R918VBY9PYKHM24	25.900	4.972	0.003	
Gly-TCC	i-tRF	tRF-50-789LV47PJPN1Y6FPZD3K	32.068	6.970	0.000	
Ile-AAT	3′-tRF	tRF-25-R95R3LMJ26	1.452	17.314	0.000	
Ile-AAT	3′-tRF	tRF-26-M3WEKSUYP5D	5.313	16.538	0.004	
Leu-CAA	3′-tRF	tRF-37-245J7KYUHRE9XFJ	9.587	25.923	0.003	
Leu-CAG	3′-tRF	tRF-25-R9OMKIWU26	0.000	14.352	0.000	
Leu-TAG	3′-tRF	tRF-42-16ZSIJ7KYUHR0VX6J	3.772	14.836	0.003	
Leu-TAG	3′-tRF	tRF-44-K3JZL4Q4R9OMK1QVFV	0.609	8.496	0.002	
Lys-CTT	5′-half	tRF-33-PSQP4PW3FJI0V	44.135	193.538	0.002	0.833 (0.052)
Lys-TTT	3’-tRF	tRF-29-KK761RNMJJI2	75.525	32.080	0.004	0.979 (0.005)
Met-CAT	3’-tRF	tRF-19-O182RKFV	58.382	140.497	0.001	0.938 (0.011)
Met-CAT	3’-tRF	tRF-29-3U7SF7J01JE2	1465.585	605.153	0.004	0.708 (0.225)
Met-CAT	3’-tRF	tRF-32-D33U7SB7J0RJ1	47.626	99.100	0.000	0.938 (0.011)
Phe-GAA	i-tRF	tRF-24-N3WEK879IU	15.230	40.874	0.003	
Ser-CGA	3’-tRF	tRF-37-ZR3FJ3WB8YYWOV2	2.425	14.123	0.003	
Ser-GCT	3’-tRF	tRF-17-HR0ZMSJ	19.384	8.489	0.004	
Ser-GCT	3′-tRF	tRF-32-0H93WB8689SV2	0.000	13.427	0.000	
Ser-GCT	3′-tRF	tRF-37-NY2V7KYUHRH7MSJ	2.621	15.273	0.003	
Ser-TGA	3′-tRF	tRF-16-3WMIE1B	1.487	12.402	0.004	
Thr-AGT	i-tRF	tRF-30-FMNKYUHRF8X7	8.124	23.767	0.002	
Thr-CGT	3′-tRF	tRF-29-387SDRJHR1J2	2776.908	1271.636	0.004	0.729 (0.182)
Thr-CGT	i-tRF	tRF-25-387SDRJHR1	45.646	12.009	0.001	
Thr-CGT	i-tRF	tRF-26-387SDRJHR1E	267.484	122.209	0.003	0.75 (0.146)
Thr-CGT	i-tRF	tRF-28-H5RNLDKW34D3	5.792	16.783	0.005	
**Thr-CGT_Met-CAT (11)**	**i-tRF**	**tRF-19-F8DHXYIV**	244.338	129.673	0.003	0.854 (0.039)
**Thr-TGT (14)**	**3′-tRF**	**tRF-17-D7LYR15**	83.461	156.086	0.004	0.938 (0.011)
Thr-TGT	i-tRF	tRF-16-KKRSL3E	2.155	12.066	0.005	
Trp-CCA	3′-tRF	tRF-19-2E2WMKJ1	3.209	18.563	0.001	
Trp-CCA	3′-tRF	tRF-29-QV7NLE2WMKJ1	1.724	11.576	0.002	
Tyr-GTA	3′-tRF	tRF-34-YKWIR959MIL0HV	29.606	58.189	0.003	0.833 (0.052)
Tyr-GTA	3′-tRF	tRF-35-NPSV9NLN3JWB61	12.216	38.525	0.001	
**Val-CAC (3)**	**3′-tRF**	**tRF-23-7SB1RHODV**	99.463	238.794	0.001	0.958 (0.008)
**Val-CAC (8)**	**3′-tRF**	**tRF-25-R9ODMJ6B26**	264.720	528.118	0.002	0.917 (0.015)
**Val-CAC (1)**	**3′-tRF**	**tRF-34-D3KS7SB1RHODE2**	127.820	253.723	0.000	1 (0.004)
Val-CAC	i-tRF	tRF-21-7SHRRHODB	3.944	19.708	0.000	
Val-CAC	i-tRF	tRF-34-LBRR33WEK8Q20U	0.609	8.419	0.004	
Val-TAC	3′-tRF	tRF-19-BZOS4YE2	2.759	13.006	0.005	
**B. PB.PC vs. BPH.PA**						
tRNA (*)	**Variant**	**tsRNA ID (MINTbase)**	**PB.PC Mean Value**	**BPH.PA Mean Value**	***p*-Value**	**AUC (*p*-Value)**
Ala-AGC	3′-tRF	tRF-20-LMKPJ7K1	110.791	63.513	0.004	0.813 (0.036)
Ala-TGC	i-tRF	tRF-20-7SHRMFWR	13.269	30.505	0.004	
Ala-TGC	i-tRF	tRF-22-R95R3JH92	59.897	105.289	0.004	0.828 (0.027)
Arg-CCT	3′-tRF	tRF-23-R95MKM9K0M	57.789	31.546	0.001	0.875 (0.012)
Arg-TCG	i-tRF	tRF-19-W08Y3H2K	23.738	14.502	0.001	
Asn-GTT	3′-half	tRF-41-7O3B1NR8YUP6KP6HD	23.628	7.817	0.000	
**Asn-GTT (6)**	**3′-half**	**tRF-41-YDLBRY73WEK5KKOVD**	98.495	182.474	0.003	0.844 (0.021)
Asn-GTT	3′-tRF	tRF-46-QWU1O0789NLJK1JKE1B	88.053	156.816	0.002	0.75 (0.093)
Asn-GTT	i-tRF	tRF-18-1NR8YUDV	5.225	13.414	0.005	
Asn-GTT	i-tRF	tRF-25-Z3R95R2R12	4.010	10.998	0.000	
**Asp-GTC (2)**	**3′-half**	**tRF-41-8HM2OSRN2NKSEK51B**	5079.598	10,492.740	0.001	0.906 (0.006)
**Asp-GTC (4)**	**3′-half**	**tRF-41-8HM2OSRNLNKSEK51B**	26,663.889	54,534.218	0.002	0.906 (0.006)
**Asp-GTC (8)**	**3′-tRF**	**tRF-43-3W2VR008R959KUMK9**	212.975	429.722	0.004	0.797 (0.046)
**Asp-GTC (7)**	**3′-tRF**	**tRF-44-NMEH623K76IR3DR2I2**	730.807	1168.610	0.004	0.875 (0.012)
**Asp-GTC (3)**	**3′-tRF**	**tRF-45-3W2VR008R959KUMKF6**	5154.126	10,895.555	0.001	0.875 (0.012)
**Asp-GTC (10)**	**3′-tRF**	**tRF-45-3W2VR008R9D9KUMKF6**	629.120	1029.586	0.004	0.828 (0.027)
Asp-GTC	3′-tRF	tRF-45-WW2VR008R959KUMKF6	19.267	42.332	0.001	
Asp-GTC	i-tRF	tRF-22-3W2VR0084	7.006	25.388	0.004	
Asp-GTC	i-tRF	tRF-42-3W2VR008R9D9KUMKH	47.995	83.852	0.005	0.734 (0.115)
Cys-GCA	5′-tRF	tRF-18-RKVPNPD4	31.417	60.166	0.000	0.891 (0.009)
Gln-CTG	i-tRF	tRF-24-RXPIN24YED	2.897	15.243	0.000	
Glu-CTC	i-tRF	tRF-20-6NMH490V	5.009	20.243	0.003	
**Glu-TTC (9)**	**3′-tRF**	**tRF-43-7Z2R1HPSR9O9337KR**	112.462	225.466	0.004	0.875 (0.012)
**Glu-TTC (5)**	**3′-tRF**	**tRF-44-7Z2R1HPSR9O9337K0V**	333.927	706.971	0.002	0.859 (0.016)
**Glu-TTC (1)**	**3′-tRF**	**tRF-45-7Z2R1HPSR9O9337KB6**	843.555	2060.741	0.000	0.875 (0.012)
Glu-TTC	3′-tRF	tRF-46-RZ81JJ6RRNLIK898O1B	70.363	126.925	0.003	0.828 (0.027)
Glu-TTC	i-tRF	tRF-18-68K87SW	16.956	35.282	0.004	
Glu-TTC	i-tRF	tRF-19-1HPSR9HW	21.946	41.178	0.004	
Glu-TTC	i-tRF	tRF-21-14PSR9O9D	24.521	44.266	0.005	
Glu-TTC	i-tRF	tRF-22-1HPSR9O9J	42.748	106.953	0.002	0.766 (0.074)
Glu-TTC	i-tRF	tRF-22-JJ6RRNLIJ	7.450	21.103	0.004	
Glu-TTC	i-tRF	tRF-30-KKM1M3WD8S74	3.125	12.076	0.004	
Glu-TTC	i-tRF	tRF-42-7Z2R1HPSR9O9337KF	418.173	937.329	0.004	0.766 (0.074)
Gly-CCC	3′-tRF	tRF-21-2NK8KEP1B	7.678	23.977	0.001	
Gly-CCC	3′-tRF	tRF-47-22YRI7XUK87SIRMJ6V1	3.076	11.248	0.004	
Gly-CCC	5′-tRF	tRF-38-PNR8YP9LON4V4R8	8.893	29.872	0.001	
Gly-CCC	5′-tRF	tRF-47-PNR8YP9LON4V4REH62J	7.027	23.729	0.003	
Gly-CCC	5’-tRF	tRF-48-PNR8YP9LON4V4REH62DJ	12.255	37.189	0.000	
Gly-CCC	5’-tRF	tRF-49-PNR8YP9LON4V4REH621K	28.399	77.318	0.000	0.922 (0.005)
Gly-CCC	5’-tRF	tRF-50-PNR8YP9LON4V4REH62K8	50.762	119.634	0.001	0.797 (0.046)
Gly-CCC	i-tRF	tRF-23-IRJ9ZH0SDZ	11.729	40.484	0.002	
Gly-GCC	i-tRF	tRF-16-2VR0PS0	27.901	64.397	0.000	0.813 (0.036)
Gly-GCC	i-tRF	tRF-16-KHM26R0	9.795	27.739	0.002	
Gly-GCC	i-tRF	tRF-17-2VR0PS4	57.590	111.176	0.003	0.781 (0.059)
Gly-TCC	3’-tRF	tRF-45-392J1Y0SR9593J2H26	115.962	244.794	0.003	0.766 (0.074)
Gly-TCC	i-tRF	tRF-16-DF7UK80	7.804	23.792	0.002	
Ile-TAT	i-tRF	tRF-19-195181JX	14.082	5.033	0.005	
Leu-TAG	3′-tRF	tRF-44-K3JZL4Q4R9OMK1QVFV	10.555	2.493	0.001	
Leu-TAG	5′-tRF	tRF-25-RPM8309MUK	55.657	95.319	0.004	0.781 (0.059)
Leu-TAG	i-tRF	tRF-19-W7WK6WIZ	4.598	15.634	0.003	
Leu-TAG	i-tRF	tRF-21-RJ7KYUHRB	13.978	3.259	0.000	
Lys-TTT	i-tRF	tRF-17-1SS2PMH	15.842	26.108	0.003	
Met-CAT	i-tRF	tRF-26-BRFKWE6MODD	11.898	4.398	0.001	
Ser-CGA	3′-tRF	tRF-29-PFR9OMNNMU1V	24.492	13.877	0.001	
Ser-GCT	3′-tRF	tRF-32-0H93WB8689SV2	17.298	2.842	0.000	
Ser-GCT	i-tRF	tRF-27-Q4R9OMKM4WK	11.003	3.081	0.002	
Thr-CGT_Met-CAT	i-tRF	i-tRF&tRF-17-F8DHXY4	4.394	16.118	0.001	
Trp-CCA	3′-tRF	tRF-19-2E2WMKJ1	20.653	8.796	0.002	
Trp-CCA	3′-tRF	tRF-29-QV7NLE2WMKJ1	12.318	5.909	0.003	
Trp-CCA	i-tRF	tRF-36-HPDJXW7SD6MSRMB	11.209	3.389	0.005	
Val-CAC	3′-tRF	tRF-31-SRMNLM3KMB01B	44.869	23.777	0.005	
Val-CAC	i-tRF	tRF-16-LBRR33E	8.184	22.321	0.003	
Val-CAC	i-tRF	tRF-18-25QB1MDJ	9.511	31.574	0.000	
Val-CAC	i-tRF	tRF-19-0HLBRR14	20.493	46.679	0.001	
Val-CAC	i-tRF	tRF-19-25QB1M1K	3.566	12.637	0.001	
Val-CAC	i-tRF	tRF-19-2VO0SR1Z	36.610	77.462	0.004	0.813 (0.036)
**C. R-PCa vs. nonR-PCa**						
**tRNA**	**Variant**	**tsRNA ID (MINTbase)**	**nonR-PCa Mean Value**	**R-PCa Mean Value**	***p*-Value**	**AUC (*p*-Value)**
Ala-AGC	3′-tRF	tRF-17-I1LQ3R1	47.869	32.200	0.003	
Ala-AGC	3′-tRF	tRF-21-2MK827K1B	762.631	3392.835	0.002	0.667 (0.405)
Ala-AGC	3′-tRF	tRF-32-113KYO8R6546J	25.044	68.351	0.000	1 (0.013)
Ala-AGC	3′-tRF	tRF-34-I0SRRNLMKPJ711	40.794	155.050	0.000	0.815 (0.116)
Ala-AGC	3′-tRF	tRF-36-E0PRRN2MK827K1B	46.627	63.910	0.004	0.704 (0.309)
Ala-TGC	3′-tRF	tRF-21-2MK8J7K1B	288.702	1840.386	0.000	0.667 (0.309)
Ala-TGC	3′-tRF	tRF-21-LMK8J7K1B	874.071	4694.255	0.002	0.704 (0.309)
Ala-TGC	3′-tRF	tRF-24-76HRMFWRE2	297.170	622.736	0.004	0.667 (0.405)
Ala-TGC	3′-tRF	tRF-25-R9DR3JH926	132.983	332.699	0.000	0.63 (0.518)
Ala-TGC	3′-tRF	tRF-32-6RM3WEKSPM852	64.351	147.863	0.003	0.741 (0.229)
Ala-TGC	3’-tRF	tRF-33-5JK87SHRMFWRV	62.587	116.968	0.002	0.815 (0.116)
Ala-TGC	3’-tRF	tRF-38-S4113KYO8R6Q46D2	291.456	811.849	0.002	0.778 (0.116)
Ala-TGC	3’-tRF	tRF-38-S4113KYU8R6Q46D2	708.536	1681.173	0.003	0.852 (0.079)
Ala-TGC	3’-tRF	tRF-40-ELXKKSR9DR3JH926	16.720	61.931	0.004	0.852 (0.079)
Ala-TGC	5’-half	tRF-32-RK9P4P9L5HJVQ	3.381	33.902	0.000	
Arg-CCT	i-tRF	tRF-21-7S1R27R3B	12.402	0.246	0.001	
Asp-GTC	3’-tRF	tRF-19-D9KUMKEV	37.558	24.737	0.002	
Asp-GTC	3’-tRF	tRF-23-76IR3DR2DV	36.587	27.636	0.004	
Gln-CTG	i-tRF	tRF-19-YOH9R80V	14.458	38.203	0.004	
Gln-CTG	i-tRF	tRF-30-FU8U76D7M461	28.577	13.774	0.001	
Gln-CTG	i-tRF	tRF-35-DRFU8U76D7M461	17.976	27.698	0.005	
Glu-TTC	i-tRF	tRF-33-0VL8K87SIRMMY	27.911	49.501	0.001	
Glu-TTC	i-tRF	tRF-42-RZ81J36RRNLIK8V8L	14.976	45.102	0.004	
Glu-TTC_Phe-GAA	i-tRF	tRF-25-K87SIRMM12	49.398	23.005	0.001	
Gly-CCC	i-tRF	tRF-20-7SIRMJ6V	24.014	12.165	0.003	
Gly-TCC	3′-tRF	tRF-38-FPZD3KYUYR66EFD2	202.089	141.088	0.004	0.667 (0.405)
Lys-CTT	3′-tRF	tRF-44-4ZD7JKW4R951KHZK1V	592.902	425.698	0.004	0.741 (0.229)
Met-CAT	3′-tRF	tRF-35-FKWE6MODJRWVU6	13.736	19.984	0.002	
Phe-GAA	3′-tRF	tRF-35-4BRRN3WEK87965	66.749	45.787	0.000	0.667 (0.405)
Pro-AGG	i-tRF	tRF-16-Q01M3KE	16.859	31.412	0.002	
Ser-GCT	i-tRF	tRF-17-OMKM4WK	89.254	89.490	0.002	0.741 (0.229)
Thr-AGT	i-tRF	tRF-19-YUHRF8JV	8.539	16.862	0.004	
Thr-CGT	3′-tRF	tRF-17-BRMR715	21.147	10.813	0.002	
Thr-CGT	3′-tRF	tRF-19-BRMR71J2	16.768	10.524	0.005	
Thr-CGT	3′-tRF	tRF-26-RNLDKW343RB	16.928	7.606	0.002	
Thr-CGT	i-tRF	tRF-18-LDKW34D3	36.448	14.880	0.001	
Thr-TGT	3′-half	tRF-42-SBKKRSL38BWSNKP92	8.892	25.503	0.005	
Thr-TGT	3′-tRF	tRF-19-2E4V9K15	12.402	0.246	0.001	
Thr-TGT	3′-tRF	tRF-35-KKRSL38BWSNKP9	37.558	24.737	0.002	
Trp-CCA	3’-tRF	tRF-19-2E2WMKJ1	36.587	27.636	0.004	
Tyr-GTA	3′-tRF	tRF-21-YUYSQSD2D	14.458	38.203	0.004	
Tyr-GTA	3′-tRF	tRF-32-1MQ8YUYSQSD2J	28.577	13.774	0.001	
Tyr-GTA	i-tRF	tRF-19-WE884UEX	17.976	27.698	0.005	
Val-CAC	5′-half	tRF-34-9Z3L7LQ3V6J9I3	27.911	49.501	0.001	

DE tsRNAs (|FC| > 1.5; *p*-value < 0.005 by DESeq). tsRNAs with more than 50 counts in any of the groups are depicted in blue. Among those, tsRNAs presenting more than 50 counts in both groups (one of them higher than 150) and showing the ability to distinguish between classes after ROC analysis, selected as candidates for further validation, are depicted in bold. Orange-shaded cells denote tsRNAs with statistically significant differences between groups and discriminative performance (AUC > 0.7, *p*-value < 0.05). * Numbers in parentheses correspond to the labelled points in Figure 1 (volcano plot).

**Table 4:** sncRNA sequences and miRPrimer2 sequences.

isomiR/tsRNA	ID_miraligner_MINTbase	RNA Sequence	miRPrimer2 Forward Primer	miRPrimer2 Reverse Primer	Cq	Melting Curve
miR-181a-2-3p	hsa-miR-181a-2-3p&NA&iso-22-05X17UNLJ	ACCACUGACCGUUGACUGUACC	gaccactgaccgttgac	tccagtttttttttttttttggtaca	35	1 curve
**isomiR-187-3p**	hsa-miR-187-3p&iso_add3p:1&iso-23-W4WY9YJ1DZ	UCGUGUCUUGUGUUGCAGCCGGU	tcgtgtcttgtgttgcag	aggtccagtttttttttttttttacc	30	1 curve
**miR-187-3p**	hsa-miR-187-3p&NA&iso-22-W4WY9YJ14	UCGUGUCUUGUGUUGCAGCCGG	gcagtcgtgtcttgtgttg	cagtttttttttttttttccggct	32	1 curve
**miR-193a-5p**	hsa-miR-193a-5p&NA&iso-22-93NYMJ5EL	UGGGUCUUUGCGGGCGAGAUGA	ggtctttgcgggcga	ggtccagtttttttttttttttcatct	29	1 curve
tRF_AsnGTT_41	Asn-GTT&3′-half&tRF-41-YDLBRY73WEK5KKOVD	UUAACCGAAAGGUUGGUGGUUCGAUCCCACCCAGGGACGCC	ccgaaaggttggtggttc	cagtttttttttttttttggcgtc	34	2 curves
**tRF_AspGTC_41**	Asp-GTC&3′-half&tRF-41-8HM2OSRN2NKSEK51B	UCACGCGGGAGACCGGGGUUCAAUUCCCCGACGGGGAGCCA	gaccggggttcaattccccgac	tccagtttttttttttttttggct	21	1 curve
**tRF_AspGTC_41b**	Asp-GTC&3′-half&tRF-41-8HM2OSRNLNKSEK51B	UCACGCGGGAGACCGGGGUUCGAUUCCCCGACGGGGAGCCA	gaccggggttcgattc	tccagtttttttttttttttggct	20	1 curve
**tRF_AspGTC_45**	Asp-GTC&3’-tRF&tRF-45-3W2VR008R959KUMKF6	CCUGUCACGCGGGAGACCGGGGUUCGAUUCCCCGACGGGGAGCCA	gggagaccggggttcgattc	tccagtttttttttttttttggct	19	1 curve
**tRF_AspGTC_45b**	Asp-GTC&3’-tRF&tRF-45-3W2VR008R9D9KUMKF6	CCUGUCACGCGGGAGACCGGGGUUCAAUUCCCCGACGGGGAGCCA	gggagaccggggttcaattccccgac	tccagtttttttttttttttggct	21	1 curve
**tRF_GluTTC_45**	Glu-TTC&3’-tRF&tRF-45-7Z2R1HPSR9O9337KB6	GUUUUCACCCAGGCGGCCCGGGUUCGACUCCCGGUGUGGGAACCA	aggcggcccgggttcgactcccggtgtg	gtccagtttttttttttttttggttc	22	1 curve
**tRF_ValCAC_34**	Val-CAC&3’-tRF&tRF-34-D3KS7SB1RHODE2	AAGGUCCCCGGUUCGAAACCGGGCGGAAACACCA	agaaggtccccggttc	gtccagtttttttttttttttggtg	27	1 curve
miR-30e-3p	hsa-miR-30e-3p&NA&iso-22-N91S5WYFM	CUUUCAGUCGGAUGUUUACAGC	* gcagctttcagtcggatgt	* tccagtttttttttttttttgctgt	30	1 curve

The RNA sequence that corresponds to the miRPrimer2 forward primer is depicted in blue, whereas that corresponding to the sequence complementary to the reverse primer is depicted in green. * Described in Ferre et al., 2023 [7]. Isoforms shown in bold were selected for subsequent analyses based on Cq values and melting curve profiles.

**Table 5:** Receiver operating characteristic (ROC) analysis showing the predictive efficiency of sncRNAs in seminal small extracellular vesicles for PCa diagnosis.

**1. miRNA-Based Models**						
**Markers**	**AUC (*p*-Value)**	**95% CI**	**Sensitivity %**	**Specificity %**	**PPV %**	**NPV %**
**A**. (HCt+BPH) vs. PCa						
miR-187-3p	0.690 (**0.020**)	0.532–0.847	100	0	55.76	0
isomiR-187-3p	0.704 (**0.012**)	0.549–0.859	100	0	55.76	0
miR-193a-5p	0.660 (**0.049**)	0.497–0.824	86.2	56.5	71.4	76.47
Combined miRNA model * (miR-193a-5p)-5p)						
**B**. BPH vs. PCa						
PSA	0.580 (0.495)	0.371–0.789	100	0	78.37	0
miR-187-3p	0.584 (0.472)	0.316–0.852	100	25	82.85	100
isomiR-187-3p	0.526 (0.825)	0.248–0.804	100	12.5	80.55	100
miR-193a-5p	0.588 (0.449)	0.312–0.865	100	0	78.37	0
Combined miRNA model (miR-187-3p+miR-193a-5p)	0.884 (**0.001**)	0.752–1.000	96.6	25	82.35	66.66
**C**. (HCt+BPH+PCa_GS6) vs. (PCa GS7+GS8)						
miR-187-3p	0.700 (**0.023**)	0.559–0.841	0	94.4	0	68
isomiR-187-3p	0.699 (**0.023**)	0.557–0.841	6.3	97.2	50	70
miR-193a-5p	0.661 (0.067)	0.513–0.808	12.5	97.2	66.67	71.4
Combined miRNA model * (miR-193a-5p)						
**D**. (BPH+PCa_GS6) vs. (PCa_GS7+GS8)						
PSA	0.670 (0.081)	0.487–0.852	31.3	76.2	50	59.26
miR-187-3p	0.568 (0.481)	0.382–0.755	0	100	0	58.3
isomiR-187-3p	0.583 (0.391)	0.397–0.769	0	100	0	58.3
miR-193a-5p	0.621 (0.214)	0.437–0.804	25	71.4	40	55.55
Combined PSA-miRNA model (PSA+miR-187-3p+miR-193a-5p)	0.705 (**0.034**)	0.535–0.876	43.8	81	63.6	65.38
Combined miRNA model (miR-187-3p+miR-193a-5p)	0.649 (0.125)	0.469–0.829)	25	81	50	58.62
**E**. (BPH+PCa_I) vs. (PCa_IIA_IIB_III)						
PSA	0.629 (0.180)	0.447–0.812	50	70.6	2	54.54
miR-187-3p	0.512 (0.903)	0.317–0.706	85	23.5	56.66	57.14
isomiR-187-3p	0.547 (0.626)	0.351–0.743	95	17.6	57.57	75
miR-193a-5p	0.522 (0.819)	0.322–0.722	100	0	54.05	0
Combined PSA-miRNA model (PSA+miR-187-3p)	0.606 (0.273)	0.421–0.791	60	64.7	66.66	57.89
Combined miRNA model * (miR-187-3p)						
**2. tsRNA-based models**						
**Markers**	**AUC (*p*-Value)**	**95% CI**	**Sensitivity %**	**Specificity %**	**PPV %**	**NPV %**
**A**. (HCt+BPH) vs. PCa						
tRF-ValCAC34	0.754 (**0.002**)	0.615–0.893	82.8	54.2	68.57	72.22
tRF-AspGTC41	0.763 (**0.001**)	0.623–0.903	75.9	66.7	73.33	69.56
tRF-AspGTC41b	0.788 (**0.000**)	0.660–0.916	65.5	70.8	73.07	62.96
tRF-AspGTC45	0.792 (**0.000**)	0.666–0.919	69	66.7	71.42	64
tRF-AspGTC45b	0.785 (**0.000**)	0.656–0.914	65.5	75	76	64.28
tRF-GluTTC45	0.802 (**0.000**)	0.673–0.931	58.6	79.4	77.27	61.29
Combined tsRNA model (Asp41+Asp45+Asp45b+Glu+Val)	0.937 (**0.000**)	0.871–1.000	89.7	87.5	89.65	87.5
**B**. BPH vs. PCa						
PSA	0.580 (0.495)	0.371–0.789	100	0	78.37	0
tRF-ValCAC34	0.659 (0.172)	0.392–0.927	100	0	78.37	0
tRF-AspGTC41	0.534 (0.768)	0.263–0.806	100	0	78.37	0
tRF-AspGTC41b	0.616 (0.319)	0.370–0.862	100	0	78.37	0
tRF-AspGTC45	0.619 (0.310)	0.375–0.863	100	0	78.37	0
tRF-AspGTC45b	0.580 (0.495)	0.323–0.836	100	0	78.37	0
tRF-GluTTC45	0.638 (0.238)	0.370–0.906	100	0	78.37	0
Combined tsRNA model (Asp41+Asp45+Glu+Val)	0.884 (**0.001**)	0.755–1.000	100	62.5	90.62	100
**C**. (HCt+BPH+PCa_GS6) vs. (PCa GS7+GS8)						
tRF-ValCAC34	0.671 (**0.049**)	0.525–0.818	6.3	97.3	50	70.59
tRF-AspGTC41	0.696 (**0.025**)	0.558–0.834	0	97.3	0	69.23
tRF-AspGTC41b	0.720 (**0.012**)	0.585–0.854	12.5	94.6	50	71.42
tRF-AspGTC45	0.712 (**0.015**)	0.576–0.848	12.5	94.6	50	71.42
tRF-AspGTC45b	0.677 (**0.042**)	0.531–0.824	18.8	91.9	50	72.34
tRF-GluTTC45	0.709 (**0.017**)	0.568–0.849	18.8	94.6	60	72.91
Combined tsRNA model (Asp41+Glu+Val)	0.807 (**0.000**)	0.692–0.923	25	94.6	66.66	74.46
**D**. (BPH+PCa_GS6) vs. (PCa_GS7+GS8)						
PSA	0.670 (0.081)	0.487–0.852	31.3	76.2	50	59.26
tRF-ValCAC34	0.572 (0.453)	0.386–0.759	0	100	0	56.75
tRF-AspGTC41	0.545 (0.646)	0.357–0.732	0	100	0	56.75
tRF-AspGTC41b	0.586 (0.374)	0.401–0.771	0	100	0	56.75
tRF-AspGTC45	0.573 (0.453)	0.387–0.759	6.3	95.2	50	57.14
tRF-AspGTC45b	0.524 (0.806)	0.334–0.714	0	100	0	56.75
tRF-GluTTC45	0.575 (0.453)	0.386–0.760	18.8	90.5	59.37	60
Combined tsRNA model (Asp41+Asp45+Asp45b+Glu)	0.729 (**0.018**)	0.565–0.893	43.8	90.5	77.78	67.85
Combined PSA-tsRNA model (PSA+Asp41+Asp41b+Asp45+Asp45b+Glu)	0.774 (**0.005**)	0.620–0.927	56.3	81	69.23	70.8
**E**. (BPH+PCa_I) vs. (PCa_IIA_IIB_III)						
PSA	0.629 (0.180)	0.447–0.812	50	70.6	2	54.54
tRF-ValCAC34	0.575 (0.437)	0.382–0.768	100	0	54.05	0
tRF-AspGTC41	0.504(0.964)	0.311–0.698	85	11.8	53.12	40
tRF-AspGTC41b	0.559 (0.542)	0.366–0.751)	100	0	54.05	0
tRF-AspGTC45	0.532 (0.737)	0.340–0.724	100	0	54.05	0
tRF-AspGTC45b	0.521 (0.831)	0.330–0.712	100	0	54.05	0
tRF-GluTTC45	0.569 (0.474)	0.376–0.762	100	0	54.05	0
Combined tsRNA model (Asp41+Asp45+Asp45b+Glu)	0.665 (0.088)	0.488–0.841	75	47.1	62.5	61.54
Combined PSA-tsRNA model (PSA+Asp41+Asp41b+Asp45+Asp45b)	0.729 (**0.017**)	0.565–0.894	70	70.6	73.68	66.66

* After multivariate logistic regression analysis, only one variable remained in the final model. isomiR-187-3p (hsa-miR-187-3p &iso_add3p:1); miR-187-3p (hsa-miR-187-3p&NA); miR-193a-5p (hsa-miR-193a-5p&NA) isomiR variants; tRF_AspGTC41 (tRF-41-8HM2OSRN2NKSEK51B); tRF_AspGTC41b (tRF-41-8HM2OSRNLNKSEK51B); tRF_AspGTC45 (tRF-45-3W2VR008R959KUMKF6); tRF_AspGTC45b (tRF-45-3W2VR008R9D9KUMKF6); tRF_GluTTC45 (tRF-45-7Z2R1HPSR9O9337KB6); tRF_ValCAC34 (tRF-34-D3KS7SB1RHODE2) tRF isoforms. AUC: area under the ROC curve; CI: confidence interval; PPV: positive predictive value; NPV: negative predictive value.

## Data Availability

Data is contained within this article and the Supplementary Materials.

## Supplementary Materials

The following supporting information can be downloaded at: https://www.mdpi.com/article/10.3390/ijms27146205/s1.

## References

[1] Oesterling J.E. (1991). Prostate specific antigen: A critical assessment of the most useful tumor marker for adenocarcinoma of the prostate. J. Urol..

[2] Hayes J.H., Barry M.J. (2014). Screening for prostate cancer with the prostate-specific antigen test: A review of current evidence. JAMA.

[3] Epstein J.I., Egevad L., Amin M.B., Delahunt B., Srigley J.R., Humphrey P.A. (2016). The 2014 International Society of Urological Pathology (ISUP) Consensus Conference on Gleason Grading of Prostatic Carcinoma: Definition of Grading Patterns and Proposal for a New Grading System. Am. J. Surg. Pathol..

[4] Vojtech L., Woo S., Hughes S., Levy C., Ballweber L., Sauteraud R.P., Strobl J., Westerberg K., Gottardo R., Tewari M. (2014). Exosomes in human semen carry a distinctive repertoire of small non-coding RNAs with potential regulatory functions. Nucleic Acids Res..

[5] Larriba S., Sánchez-Herrero J.F., Pluvinet R., López-Rodrigo O., Bassas L., Sumoy L. (2024). Seminal extracellular vesicle sncRNA sequencing reveals altered miRNA/isomiR profiles as sperm retrieval biomarkers for azoospermia. Andrology.

[6] Barceló M., Castells M., Bassas L., Vigués F., Larriba S. (2019). Semen miRNAs Contained in Exosomes as Non-Invasive Biomarkers for Prostate Cancer Diagnosis. Sci. Rep..

[7] Ferre A., Santiago L., Sánchez-Herrero J.F., López-Rodrigo O., Sánchez-Curbelo J., Sumoy L., Bassas L., Larriba S. (2023). 3′IsomiR Species Composition Affects Reliable Quantification of miRNA/isomiR Variants by Poly(A) RT-qPCR: Impact on Small RNA-Seq Profiling Validation. Int. J. Mol. Sci..

[8] Choy K.H.K., Chan S.Y., Lam W., Jin J., Zheng T., Law T.Y.S., Yu S.S., Wang W., Li L., Xie G. (2022). The repertoire of testicular extracellular vesicle cargoes and their involvement in inter-compartmental communication associated with spermatogenesis. BMC Biol..

[9] Zedan A.H., Blavnsfeldt S.G., Hansen T.F., Nielsen B.S., Marcussen N., Pleckaitis M., Osther P.J.S., Sørensen F.B. (2017). Heterogeneity of miRNA expression in localized prostate cancer with clinicopathological correlations. PLoS ONE.

[10] Magee R.G., Telonis A.G., Loher P., Londin E., Rigoutsos I. (2018). Profiles of miRNA Isoforms and tRNA Fragments in Prostate Cancer. Sci. Rep..

[11] Liu W., Yu M., Cheng S., Zhou X., Li J., Lu Y., Liu P., Ding S. (2023). tRNA-Derived RNA Fragments Are Novel Biomarkers for Diagnosis, Prognosis, and Tumor Subtypes in Prostate Cancer. Curr. Oncol..

[12] Olvedy M., Scaravilli M., Hoogstrate Y., Visakorpi T., Jenster G., Martens-Uzunova E.S. (2016). A comprehensive repertoire of tRNA-derived fragments in prostate cancer. Oncotarget.

[13] Wang H., Liu C., Dong Z., Chen X., Zhang P. (2024). Prostate-specific antigen, androgen, progesterone and oestrogen receptors in Benign prostatic hyperplasia: Human tissues and animal model study. Aging Male.

[14] Oseni S.O., Naar C., Pavlović M., Asghar W., Hartmann J.X., Fields G.B., Esiobu N., Kumi-Diaka J. (2023). The Molecular Basis and Clinical Consequences of Chronic Inflammation in Prostatic Diseases: Prostatitis, Benign Prostatic Hyperplasia, and Prostate Cancer. Cancers.

[15] Ferre-Giraldo A., Castells M., Sánchez-Herrero J.F., López-Rodrigo O., de Rocco-Ponce M., Bassas L., Vigués F., Sumoy L., Larriba S. (2024). Semen sEV tRF-Based Models Increase Non-Invasive Prediction Accuracy of Clinically Significant Prostate Cancer among Patients with Moderately Altered PSA Levels. Int. J. Mol. Sci..

[16] Paziewska A., Mikula M., Dabrowska M., Kulecka M., Goryca K., Antoniewicz A., Dobruch J., Borowka A., Rutkowski P., Ostrowski J. (2018). Candidate diagnostic miRNAs that can detect cancer in prostate biopsy. Prostate.

[17] Casanova-Salas I., Rubio-Briones J., Calatrava A., Mancarella C., Masiá E., Casanova J., Fernández-Serra A., Rubio L., Ramírez-Backhaus M., Armiñán A. (2014). Identification of miR-187 and miR-182 as biomarkers of early diagnosis and prognosis in patients with prostate cancer treated with radical prostatectomy. J. Urol..

[18] Casanova-Salas I., Masiá E., Armiñán A., Calatrava A., Mancarella C., Rubio-Briones J., Scotlandi K., Vicent M.J., López-Guerrero J.A. (2015). MiR-187 Targets the Androgen-Regulated Gene ALDH1A3 in Prostate Cancer. PLoS ONE.

[19] Li C., Sun Z., Song Y., Zhang Y. (2022). Suppressive function of bone marrow-derived mesenchymal stem cell-derived exosomal microRNA-187 in prostate cancer. Cancer Biol. Ther..

[20] Walter B.A., Valera V.A., Pinto P.A., Merino M.J. (2013). Comprehensive microRNA Profiling of Prostate Cancer. J. Cancer.

[21] Yang Z., Chen J.S., Wen J.K., Gao H.T., Zheng B., Qu C.B., Liu K.L., Zhang M.L., Gu J.F., Li J.D. (2017). Silencing of miR-193a-5p increases the chemosensitivity of prostate cancer cells to docetaxel. J. Exp. Clin. Cancer Res. CR.

[22] Fink K.G., Hutarew G., Lumper W., Jungwirth A., Dietze O., Schmeller N.T. (2001). Prostate cancer detection with two sets of ten-core compared with two sets of sextant biopsies. Urology.

[23] Buyyounouski M.K., Choyke P.L., McKenney J.K., Sartor O., Sandler H.M., Amin M.B., Kattan M.W., Lin D.W. (2017). Prostate cancer—Major changes in the American Joint Committee on Cancer eighth edition cancer staging manual. CA A Cancer J. Clin..

[24] Li H., Huang S., Guo C., Guan H., Xiong C. (2012). Cell-free seminal mRNA and microRNA exist in different forms. PLoS ONE.

[25] Plata-Peña L., López-Rodrigo O., Bassas L., Larriba S. (2023). Experimental validation of seminal miR-31-5p as biomarker for azoospermia and evaluation of the effect of preanalytical variables. Andrology.

[26] Sanchez Herrero J.F., Pluvinet R., Luna de Haro A., Sumoy L. (2021). Paired-end small RNA sequencing reveals a possible overestimation in the isomiR sequence repertoire previously reported from conventional single read data analysis. BMC Bioinform..

[27] Martín M. (2011). Cutadapt removes adapter sequences from high-throughput sequencing reads. EMBnet.journal.

[28] Langmead B., Trapnell C., Pop M., Salzberg S.L. (2009). Ultrafast and memory-efficient alignment of short DNA sequences to the human genome. Genome Biol..

[29] Liao Y., Smyth G.K., Shi W. (2014). featureCounts: An efficient general purpose program for assigning sequence reads to genomic features. Bioinformatics.

[30] The RNAcentral Consortium (2019). RNAcentral: A hub of information for non-coding RNA sequences. Nucleic Acids Res..

[31] Pantano L., Estivill X., Martí E. (2010). SeqBuster, a bioinformatic tool for the processing and analysis of small RNAs datasets, reveals ubiquitous miRNA modifications in human embryonic cells. Nucleic Acids Res..

[32] Kozomara A., Birgaoanu M., Griffiths-Jones S. (2019). miRBase: From microRNA sequences to function. Nucleic Acids Res..

[33] FigureDesvignes T., Loher P., Eilbeck K., Ma J., Urgese G., Fromm B., Sydes J., Aparicio-Puerta E., Barrera V., Espín R. (2020). Unification of miRNA and isomiR research: The mirGFF3 format and the mirtop API. Bioinformatics.

[34] Loher P., Telonis A.G., Rigoutsos I. (2017). MINTmap: Fast and exhaustive profiling of nuclear and mitochondrial tRNA fragments from short RNA-seq data. Sci. Rep..

[35] Patil A.H., Halushka M.K. (2021). miRge3.0: A comprehensive microRNA and tRF sequencing analysis pipeline. NAR. Genom. Bioinform..

[36] Love M.I., Huber W., Anders S. (2014). Moderated estimation of fold change and dispersion for RNA-seq data with DESeq2. Genome Biol..

[37] Zhu A., Ibrahim J.G., Love M.I. (2019). Heavy-tailed prior distributions for sequence count data: Removing the noise and preserving large differences. Bioinformatics.

[38] Balcells I., Cirera S., Busk P.K. (2011). Specific and sensitive quantitative RT-PCR of miRNAs with DNA primers. BMC Biotechnol..

[39] Fawcett T. (2006). An introduction to ROC analysis. Pattern Recognit. Lett..

[40] Skendzel L.P., Youden W.J. (1970). Systematic versus random error in laboratory surveys. Am. J. Clin. Pathol..

[41] Fan Y., Siklenka K., Arora S.K., Ribeiro P., Kimmins S., Xia J. (2016). miRNet—Dissecting miRNA-target interactions and functional associations through network-based visual analysis. Nucleic Acids Res..

[42] Zhou Y., Peng H., Cui Q., Zhou Y. (2021). tRFTar: Prediction of tRF-target gene interactions via systemic re-analysis of Argonaute CLIP-seq datasets. Methods.

[43] Liu Q., Ding C., Lang X., Guo G., Chen J., Su X. (2021). Small noncoding RNA discovery and profiling with sRNAtools based on high-throughput sequencing. Brief. Bioinform..

