# Supplementary material for: Altered sncRNA Signatures in Semen Extracellular Vesicles Between Patients with Benign and Malignant Prostate Disease as Potential Non-Invasive Biomarkers in the PSA Grey Zone
Source: Int J Mol Sci. 2026 Jul 11;27(14):6205. doi: 10.3390/ijms27146205 (PMC13409904; doi:10.3390/ijms27146205)
Supplement: Supplementary file 1 [file ijms-27-06205-s001.zip › ijms-4377470-supplementary.pdf]

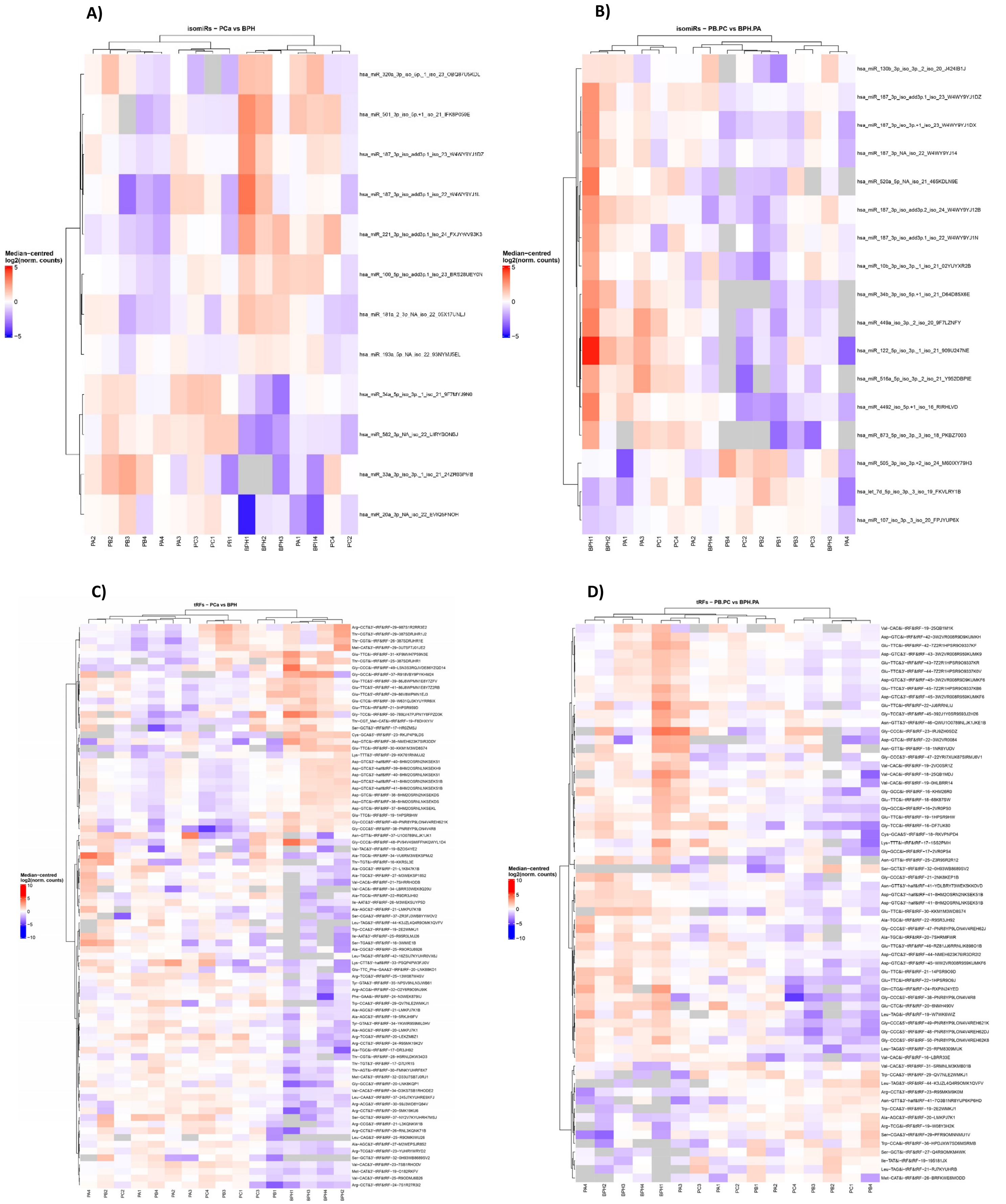

Supplementary Figure S1. Unsupervised hierarchical clustering of differentially expressed small non-coding RNAs in semen sEV samples.

Heatmaps display median-centred, log2-transformed normalised counts for statistically significant (A, B) isomiRs and (C, D) tRNA-derived fragments (tRFs) in two comparisons: (A, C) prostate cancer (PCa, n = 12) versus benign prostatic hyperplasia (BPH, n = 4), and (B, D) high-grade cancer categories (PB + PC, n = 8) versus low-grade/benign categories (BPH + PA, n = 8). Features were selected by DESeq2 differential expression analysis at a nominal *p*-value threshold of $p < 0.005$. After cross-checking and quality filtering, 12 isomiRs (panel A), 17 isomiRs (panel B), 84 tRFs (panel C), and 66 tRFs (panel D) were retained for visualisation.

Each column represents one patient sample and each row one feature. Raw normalised counts were log2-transformed and then median-centred per feature (each row's median expression subtracted), so that colour reflects deviation from the feature's own median rather than absolute abundance. The colour scale runs from blue (below-median expression) through white (at median) to red (above-median expression), with the scale limits set symmetrically to the maximum absolute deviation observed in each dataset. Grey cells indicate missing values (counts of zero after log2 transformation). Both rows (features) and columns (samples) were clustered independently using agglomerative hierarchical clustering with Pearson correlation as the distance metric and Ward's D2 linkage. No a priori group information was used during clustering. Heatmaps were generated with the ComplexHeatmap R package (v2.x).

BPH, benign prostatic hyperplasia; isomiR, isoform of a microRNA; PA/PB/PC, prostate cancer Gleason score categories; PCa, prostate cancer; tRF, tRNA-derived fragment.

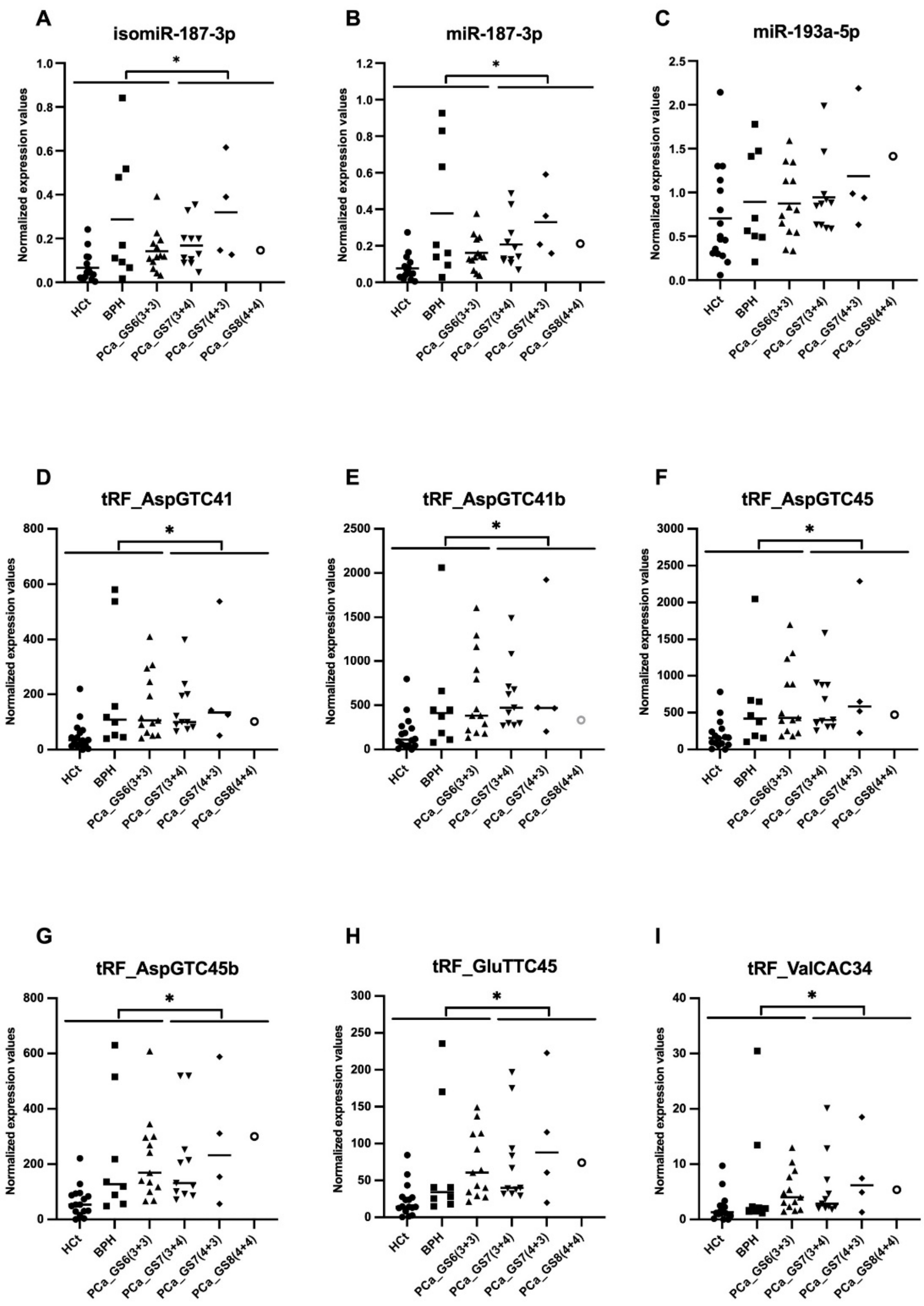

Supplementary Figure S2. sncRNA isoform levels in PCa samples with different tumour severities defined by Gleason Score

Expression levels of selected isomiR and tRF isoforms in seminal small extracellular vesicles (sEVs) were quantified by miRPrimer2 reverse transcriptase–quantitative real-time polymerase chain reaction (RT-qPCR). The following isoforms are shown: hsa-miR-187-3p iso_add3p:1 (A), hsa-miR-187-3p (B), hsa-miR-193a-5p (C), tRF_AspGTC41 (tRF-41-8HM2OSRN2NKSEK51B) (D), tRF_AspGTC41b (tRF-41-8HM2OSRNLNKSEK51B) (E), tRF_AspGTC45 (tRF-45-3W2VR008R959KUMKF6) (F), tRF_AspGTC45b (tRF-45-3W2VR008R9D9KUMKF6) (G), tRF_GluTTC45 (tRF-45-7Z2R1HPSR9O9337KB6) (H) and tRF_ValCAC34 (tRF-34-D3KS7SB1RHODE2) (I). Data are presented as relative quantification (RQ) values calculated using the 2−ΔCq strategy, normalised to miR-30e-3p expression. Horizontal bars indicate median values. The horizontal bar displays the median expression value. Significant differences between groups are indicated: * *p*-value < 0.05 (Mann–Whitney U-test).

HCt: healthy controls; BPH: benign prostatic hyperplasia; PCa (GS6): Gleason 6 (the least aggressive) classified prostate cancer; PCa (GS7): Gleason 7 (a medium grade) classified prostate cancer; PCa (GS8): Gleason 8 (aggressive) classified prostate cancer.

Supplementary Table S1. Clinical data of individuals included in the study of small RNA content of semen extracellular vesicles in prostate cancer.

Patient No.	Subgroups	Age (years)	Vasectomized?	PSA ng/ml (pre-biopsy)	Gleason score (biopsy) GS-B	Clinical stage (cT+N+M)	Prognostic group*	Treatment	Gleason score (surgery) GS-S	Pathologic stage (pT+N)
1	HCt_noV	37	no	nd	--	--	--	--	--	--
2	HCt_noV	37	no	nd	--	--	--	--	--	--
3	HCt_noV	42	no	nd	--	--	--	--	--	--
4	HCt_noV	40	no	nd	--	--	--	--	--	--
5	HCt_noV	44	no	nd	--	--	--	--	--	--
6	HCt_V	32	yes	nd	--	--	--	--	--	--
7	HCt_V	35	yes	nd	--	--	--	--	--	--
8	HCt_V	36	yes	nd	--	--	--	--	--	--
9	HCt_V	39	yes	nd	--	--	--	--	--	--
10	HCt_V	38	yes	nd	--	--	--	--	--	--
11	HCt_V	32	yes	nd	--	--	--	--	--	--
12	HCt_V	36	yes	nd	--	--	--	--	--	--
13	HCt_V	65	yes	nd	--	--	--	--	--	--
14	HCt_V	36	yes	nd	--	--	--	--	--	--
15	HCt_V	41	yes	nd	--	--	--	--	--	--
16	HCt_V	48	yes	nd	--	--	--	--	--	--
17	BPH	59	no	6.07		--	--	--	--	--
18	BPH	56	no	4.68	--	--	--	--	--	--
19	BPH/PIN-AG	63	no	8.42	--	--	--	--	--	--
20	BPH	61	no	5.57	--	--	--	--	--	--
21	BPH	64	no	9.14	--	--	--	--	--	--
22	BPH	52	no	4.7	--	--	--	--	--	--
23	BPH/PIN-AG	53	no	5.93	--	--	--	--	--	--
24	BPH	61	no	4.97	--	--	--	--	--	--
25	PCa_noV	58	no	5.97	6(3+3)	cT1c_NX_MX	I	RP	7(3+4)	pT2c_NX
26	PCa_noV	59	no	6.85	6(3+3)	cT1c_N0_MX	I	RP	7(4+3)	pT2c_NX
27	PCa_noV	53	no	5.03	6(3+3)	cT1c_NX_MX	I	RP	6(3+3)	pT2c_NX
28	PCa_noV	51	no	8.35	6(3+3)	cT1c_N0_MX	I	RP	7(3+4)	pT3a_NX
29	PCa_noV	59	no	4.97	6(3+3)	cT1c_NX_MX	I	RP	6(3+3)	pT2c_NX
30	PCa_noV	53	no	9.4	6(3+3)	cT1c_N0_MX	I	RP	6(3+3)	pT2c_NX
31	PCa_V	50	yes	4.9	6(3+3)	cT1c_NX_MX	I	RP	6(3+3)	pT2c_NX
32	PCa_V	67	yes	5.41	6(3+3)	cT2a_N0_MX	I	AS	nd	nd
33	PCa_noV	50	no	4.5	6(3+3)	cT1c_N0_MX	I	RP	7(3+4)	pT2c_NX
34	PCa_noV	61	no	10	6(3+3)	cT1c_N0_MX	IIA	RP	6(3+3)	pT2c_NX
35	PCa_noV	53	no	4.38	6(3+3)	cT2c_N0_MX	IIA	RP	7(3+4)	pT2c_NX
36	PCa_noV	62	no	5.1	6(3+3)	cT2c_N0_MX	IIA	AS	nd	nd
37	PCa_V	67	yes	5.86	6(3+3)	cT2c_N0_MX	IIA	AS	nd	nd
38	PCa_noV	59	no	4.25	7(3+4)	cT1c_N0_MX	IIB	RP	7(3+4)	pT2c_NX
39	PCa_noV	59	no	6.8	7(3+4)	cT1c_NX_MX	IIB	RP	7(3+4)	pT2c_NX
40	PCa_noV	72	no	5.64	7(3+4)	cT1c_N0_MX	IIB	AS	nd	nd
41	PCa_noV	52	no	4.64	7(3+4)	cT1c_N0_MX	IIB	RP	7(3+4)	pT2c_NX
42	PCa_noV	71	no	5.68	7(3+4)	cT2c_N0_M0	IIB	TULSA-PRO	nd	nd
43	PCa_noV	67	no	10.46	7(3+4)	cT2c_N0_MX	IIB	AS	nd	nd
44	PCa_V	68	yes	5.19	7(3+4)	cT1c_N0_MX	IIB	AS	nd	nd
45	PCa_V	43	yes	11.9	7(3+4)	cT2c_N0_MX	IIB	RP	7(3+4)	pT2c_NX
46	PCa_V	55	yes	17	7(3+4)	cT2c_N0_MX	IIB	RP+LDN	7(3+4)	pT2c_N0
47	PCa_noV	56	no	5.99	7(4+3)	cT1c_NX_MX	IIC	RP	7(4+3)	pT3a_NX
48	PCa_noV	63	no	6.28	7(4+3)	cT2c_N0_MX	IIC	RP	7(4+3)	pT3a_NX
49	PCa_noV	57	no	7	7(4+3)	cT1c_NX_MX	IIC	RP	7(4+3)	pT2c_NX
50	PCa_noV	68	no	5.75	8(4+4)	cT1c_N0_M0	IIC	RP	8(4+4)	pT2c_N0
51	PCa_V	56	yes	12.51	7(3+4)	cT3a_N0_M1	IIIB	RP+LDN	7(3+4)	pT2c_N0
52	PCa_noV	62	no	7.61	7(3+4)	cT3a_N0_M0	IIIB	RP+LDN	7(4+3)	pT3b_N1
53	PCa_noV	54	no	17.7	7(4+3)	cT3a_N0_M0	IIIB	RP+LDN	7(4+3)	pT3a_N0

HCt: healthy control; BPH: benign prostate hyperplasia; HGPIN: high-grade prostatic intraepithelial neoplasia; PCa_noV: prostate cancer in a non-vasectomized individual; PCa_V: prostate cancer in a vasectomized individual; PSA: prostate-specific antigen; T: primary tumor; N: nodal status; M: distal metastasis; RP: radical prostatectomy; AS: active surveillance; LDN: lymphadenectomy; nd: non determined. * American Joint Committee on Cancer Prognostic Stage grouping (8th edition). Samples depicted in blue refer to those samples included in sRNAseq profiling phase of the study

Supplementary Table S2. Expression comparison of pre-selected isomiRs variants with the rest of the variants of the isomiR family in semen EV

A. PCa vs BPH									
miRNA	Variant	UID	Sequence	Seq length	baseMean	NGS normalized counts in BPH	NGS normalized counts in PCA	*p*-value	padj
hsa-miR-100-5p	iso_add3p:1	iso-23-BRS28UEY0N	AACCCGTAGATCCGAACTTGTGT	23	414,177309	685,620788	323,69615	0,00100546	0,35978685
hsa-miR-100-5p	NA	iso-22-BRS28UEYP	AACCCGTAGATCCGAACTTGTG	22		3073,00472	1925,78811	0,07327274	0,9872995
hsa-miR-100-5p	iso_3p:+1	iso-23-BRS28UEY04	AACCCGTAGATCCGAACTTGTGG	23		13,6224493	7,20612476	0,3499162	0,9872995
hsa-miR-100-5p	iso_3p:-1	iso-21-BRS28UEYE	AACCCGTAGATCCGAACTTGT	21		3407,87675	1950,88357	0,04770567	0,9872995
hsa-miR-100-5p	iso_3p:-2	iso-20-BRS28UEY	AACCCGTAGATCCGAACTTG	20		261,710925	161,709703	0,14919089	0,9872995
hsa-miR-100-5p	iso_3p:-3	iso-19-BRS28UEQ	AACCCGTAGATCCGAACTT	19		117,503954	60,8529522	0,01164815	0,8408049
hsa-miR-100-5p	iso_add3p:1	iso-22-BRS28UEYO	AACCCGTAGATCCGAACTTGTA	22		41,3993456	28,3243029	0,29343784	0,9872995
hsa-miR-100-5p	iso_add3p:1	iso-23-BRS28UEY0L	AACCCGTAGATCCGAACTTGTGA	23		2503,93318	1444,4992	0,03922881	0,9872995
hsa-miR-100-5p	iso_add3p:2	iso-24-BRS28UEYJ6	AACCCGTAGATCCGAACTTGTGAA	24		76,5781985	41,0303747	0,08944791	0,9872995
hsa-miR-181a-2-3p	NA	iso-22-05X17UNLJ	ACCACTGACCGTTGACTGTACC	22	79,303727	145,437442	57,2591553	0,00012803	0,14252621
hsa-miR-181a-2-3p	iso_3p:-1	iso-21-05X17UNLD	ACCACTGACCGTTGACTGTAC	21		18,0696547	10,8159505	0,61035769	0,9872995
hsa-miR-181a-2-3p	iso_add3p:1	iso-22-05X17UNL3	ACCACTGACCGTTGACTGTACT	22		11,1923388	7,33774275	0,29718352	0,9872995
hsa-miR-187-3p	iso_add3p:1	iso-23-W4WY9YJ1DZ	TCGTGTCTTGTGTTGCAGCCGGT	23	184,83757	364,585801	124,921493	0,00040652	0,21820013
hsa-miR-187-3p	NA	iso-22-W4WY9YJ14	TCGTGTCTTGTGTTGCAGCCGG	22		262,336682	99,1958865	0,0062162	0,74689456
hsa-miR-187-3p	iso_3p:+1	iso-23-W4WY9YJ1DX	TCGTGTCTTGTGTTGCAGCCGGA	23		333,61752	108,544702	0,00565869	0,74689456
hsa-miR-187-3p	iso_3p:-1	iso-21-W4WY9YJ10	TCGTGTCTTGTGTTGCAGCCG	21		173,781668	92,1854975	0,0476705	0,9872995
hsa-miR-187-3p	iso_3p:-2	iso-20-W4WY9YJ1	TCGTGTCTTGTGTTGCAGCC	20		45,3163994	24,6832064	0,19601416	0,9872995
hsa-miR-187-3p	iso_add3p:1	iso-22-W4WY9YJ1L	TCGTGTCTTGTGTTGCAGCCGA	22		88,2874944	19,9776193	0,00309375	0,74689456
hsa-miR-187-3p	iso_add3p:1	iso-22-W4WY9YJ1N	TCGTGTCTTGTGTTGCAGCCGT	22		57,702626	20,9825781	0,02228611	0,93820162
hsa-miR-187-3p	iso_add3p:2	iso-24-W4WY9YJ12B	TCGTGTCTTGTGTTGCAGCCGGTA	24		37,3696372	13,0489502	0,02347754	0,95106195
hsa-miR-187-3p	iso_add3p:2	iso-24-W4WY9YJ12E	TCGTGTCTTGTGTTGCAGCCGGTT	24		80,0851104	36,9954377	0,0206514	0,8867711
hsa-miR-187-3p	iso_add3p:3	iso-25-W4WY9YJ1RZ	TCGTGTCTTGTGTTGCAGCCGGTTT	25		36,8267207	15,6755991	0,01986797	0,88264071
hsa-miR-193a-5p	NA	iso-22-93NYMJ5EL	TGGGTCTTTGCGGGCGAGATGA	22	227,259676	321,181526	195,952393	0,00336124	0,74689456
hsa-miR-193a-5p	iso_3p:-1	iso-21-93NYMJ5E0	TGGGTCTTTGCGGGCGAGATG	21		132,505394	98,364944	0,10112551	0,9872995
hsa-miR-193a-5p	iso_3p:-2	iso-20-93NYMJ5E	TGGGTCTTTGCGGGCGAGAT	20		73,4745963	56,809097	0,31001879	0,9872995
hsa-miR-193a-5p	iso_3p:-3	iso-19-93NYMJHX	TGGGTCTTTGCGGGCGAGA	19		20,617662	12,5559894	0,14943484	0,9872995
hsa-miR-193a-5p	iso_add3p:1	iso-22-93NYMJ5EN	TGGGTCTTTGCGGGCGAGATGT	22		25,7675432	17,8654121	0,29181361	0,9872995
hsa-miR-193a-5p	iso_add3p:1	iso-23-93NYMJ5ED6	TGGGTCTTTGCGGGCGAGATGAA	23		19,79583	14,7227961	0,38193167	0,9872995
hsa-miR-193a-5p	iso_add3p:1	iso-23-93NYMJ5ED7	TGGGTCTTTGCGGGCGAGATGAT	23		42,9714306	32,6745024	0,39240726	0,9872995
hsa-miR-221-3p	iso_add3p:1	iso-24-FXJYWV93K3	AGCTACATTGTCTGCTGGGTTTCT	24	86,69754	180,426128	55,4546774	0,00013277	0,14252621
hsa-miR-221-3p	NA	iso-23-FXJYWV9305	AGCTACATTGTCTGCTGGGTTTC	23		739,19271	271,060362	0,01513486	0,85623583
hsa-miR-221-3p	iso_3p:+1	iso-24-FXJYWV93K2	AGCTACATTGTCTGCTGGGTTTCA	24		71,6869075	24,1984792	0,0410439	0,9872995
hsa-miR-221-3p	iso_3p:-1	iso-22-FXJYWV93Q	AGCTACATTGTCTGCTGGGTTT	22		2187,18015	1180,42693	0,15380613	0,9872995
hsa-miR-221-3p	iso_3p:-2	iso-21-FXJYWV93E	AGCTACATTGTCTGCTGGGTT	21		1583,40557	740,326995	0,20733253	0,9872995
hsa-miR-221-3p	iso_3p:-3	iso-20-FXJYWV93	AGCTACATTGTCTGCTGGGT	20		528,796092	223,157807	0,08036198	0,9872995
hsa-miR-221-3p	iso_add3p:1	iso-23-FXJYWV930O	AGCTACATTGTCTGCTGGGTTTA	23		299,437762	138,670658	0,26444457	0,9872995
hsa-miR-221-3p	iso_add3p:1	iso-23-FXJYWV930Q	AGCTACATTGTCTGCTGGGTTTT	23		434,295368	211,863407	0,07418128	0,9872995
hsa-miR-221-3p	iso_add3p:2	iso-23-FXJYWV930F	AGCTACATTGTCTGCTGGGTTAA	23		123,848688	70,4302394	0,25783801	0,9872995
hsa-miR-221-3p	iso_add3p:2	iso-23-FXJYWV930I	AGCTACATTGTCTGCTGGGTTAT	23		79,7674273	52,5386761	0,54324468	0,9872995

B. PB.PC vs BPH.PA									
miRNA	Variant	UID	Sequence	Seq length	baseMean	NGS normalized counts in BPH.PA	NGS normalized counts in PB.PC	*p*-value	padj
hsa-miR-107	iso_3p:-3	iso-20-FPJYUP6X	AGCAGCATTGTACAGGGCTA---	20	66,031159	49,9126446	82,1496733	0,00310248	0,51262436
hsa-miR-107	NA	iso-23-FPJYUP6X02		23		62,7794715	57,541529	0,50655727	0,9967231
hsa-miR-107	iso_3p:-1	iso-22-FPJYUP6X5		22		54,9513259	50,8909381	0,7288983	0,9967231
hsa-miR-107	iso_3p:-2	iso-21-FPJYUP6XE		21		355,398759	388,606295	0,74814925	0,9967231
hsa-miR-107	iso_add3p:1	iso-23-FPJYUP6X03		23		68,8232552	84,9446405	0,74347178	0,9967231
hsa-miR-107	iso_add3p:2	iso-23-FPJYUP6X0F		23		21,8677404	21,414741	0,48020216	0,9967231
hsa-miR-107	iso_add3p:2	iso-23-FPJYUP6X0Q		23		15,8132785	22,0139638	0,30021641	0,9967231
hsa-miR-187-3p	iso_add3p:1	iso-23-W4WY9YJ1DZ	TCGTGTCTTGTGTTGCAGCCGGT	23	184,83757	255,081584	114,593556	0,00056595	0,30391705
hsa-miR-187-3p	NA	iso-22-W4WY9YJ14	TCGTGTCTTGTGTTGCAGCCGG	22		193,656203	86,3059681	0,0020124	0,46689218
hsa-miR-187-3p	iso_3p:+1	iso-23-W4WY9YJ1DX	TCGTGTCTTGTGTTGCAGCCGGA	23		231,349985	98,275828	0,00215041	0,46689218
hsa-miR-187-3p	iso_3p:-1	iso-21-W4WY9YJ10	TCGTGTCTTGTGTTGCAGCCG	21		132,602586	92,566495	0,11884947	0,9967231
hsa-miR-187-3p	iso_3p:-2	iso-20-W4WY9YJ1	TCGTGTCTTGTGTTGCAGCC	20		37,4811857	22,2018236	0,0160527	0,93493338
hsa-miR-187-3p	iso_add3p:1	iso-22-W4WY9YJ1L	TCGTGTCTTGTGTTGCAGCCGA	22		55,8358127	18,2743635	0,01008385	0,80222666
hsa-miR-187-3p	iso_add3p:1	iso-22-W4WY9YJ1N	TCGTGTCTTGTGTTGCAGCCGT	22		41,3347581	18,9904221	8,0619E-05	0,17316855
hsa-miR-187-3p	iso_add3p:2	iso-24-W4WY9YJ12B	TCGTGTCTTGTGTTGCAGCCGGTA	24		27,6873088	10,5709351	0,00038881	0,27838668
hsa-miR-187-3p	iso_add3p:2	iso-24-W4WY9YJ12E	TCGTGTCTTGTGTTGCAGCCGGTT	24		63,1069903	32,4287215	0,00920468	0,77140781
hsa-miR-187-3p	iso_add3p:3	iso-25-W4WY9YJ1RZ	TCGTGTCTTGTGTTGCAGCCGGTTT	25		29,0752778	12,8514811	0,01283366	0,87058739

Preselected isomiR are depicted in blue. Those isomiRs presenting more NGS counts that the preselected isomiR are depicted in yellow. NA variant refers to canonical miRNA sequences

Supplementary Table S3. Expression comparison of pre-selected tsRNA variants with the rest of the variants of the tsRNA family in semen EV

A. PCa vs BPH								
tRNA	Variant	tsRNA ID (MINTbase)	Sequence	Seq length	PCa_vs_BPH_pvalue	baseMean	NGS normalized counts in BPH (mean)	NGS normalized counts in PCa (mean)
Ala-AGC	3'-tRF	tRF-21-LMKPJ7K1B	CGATCCCCAGCATCTCCACCA	21	0,00190066	165,186326	98,6909948	187,351436
		tRF-22-WEK6PM852	TCGATCCCCAGCATCTCCACCA	22	0,059160702	356,105558	286,624615	379,265872
		tRF-23-YU8RFQ46D2	TTCGATCCCCAGCATCTCCACCA	23	0,042887454	148,146018	107,355048	161,743008
		tRF-24-7SHRJFWRE2	GTTCGATCCCCAGCATCTCCACCA	24	0,212188517	466,656574	394,078061	490,849411
		tRF-25-R95RKJH926	GGTTCGATCCCCAGCATCTCCACCA	25	0,606182688	33,8971999	28,349581	35,7464063
		tRF-26-RNLMKPJ7K1B	GGGTTCGATCCCCAGCATCTCCACCA	26	0,12956504	37,8665708	25,8305099	41,8785911
		tRF-27-M3WEK6PM852	CGGGTTCGATCCCCAGCATCTCCACCA	27	0,032442229	192,729329	138,884319	210,677666
		tRF-28-3KYU8RFQ46D2	CCGGGTTCGATCCCCAGCATCTCCACCA	28	0,588827197	138,817078	127,677326	142,530329
		tRF-29-K87SHRJFWRE2	CCCGGGTTCGATCCCCAGCATCTCCACCA	29	0,088252295	5340,93292	6683,62556	4893,36871
		tRF-30-8SR95RKJH926	TCCCGGGTTCGATCCCCAGCATCTCCACCA	30	0,346295033	2575,96946	3010,51178	2431,12202
		tRF-31-SRRNLMKPJ7K1B	GTCCCGGGTTCGATCCCCAGCATCTCCACCA	31	0,206015079	94,461655	76,0943019	100,584106
		tRF-32-RRM3WEK6PM852	GGTCCCGGGTTCGATCCCCAGCATCTCCACCA	32	0,665521232	94,7278406	87,9944897	96,9722908
		tRF-33-1M3KYU8RFQ46D2	AGGTCCCGGGTTCGATCCCCAGCATCTCCACCA	33	0,800855703	189,541787	185,046625	191,040174
		tRF-34-53K87SHRJFWRE2	GAGGTCCCGGGTTCGATCCCCAGCATCTCCACCA	34	0,866486216	132,937861	127,392758	134,786228
		tRF-35-XK8SR95RKJH926	TGAGGTCCCGGGTTCGATCCCCAGCATCTCCACCA	35	0,696716373	110,589629	102,73407	113,208148
		tRF-36-I0SRRNLMKPJ7K1B	ATGAGGTCCCGGGTTCGATCCCCAGCATCTCCACCA	36	0,284190626	360,717297	288,738495	384,710231
		tRF-37-JURRM3WEK6PM852	CATGAGGTCCCGGGTTCGATCCCCAGCATCTCCACCA	37	0,3559747	137,101325	118,77403	143,210423
		tRF-38-P41M3KYU8RFQ46D2	GCATGAGGTCCCGGGTTCGATCCCCAGCATCTCCACCA	38	0,968877206	190,644502	184,869635	192,569458
		tRF-39-XQ53K87SHRJFWRE2	TGCATGAGGTCCCGGGTTCGATCCCCAGCATCTCCACCA	39	0,72451481	118,879037	123,844199	117,223983
		tRF-40-IFXK8SR95RKJH926	ATGCATGAGGTCCCGGGTTCGATCCCCAGCATCTCCACCA	40	ND	ND	ND	ND
tRNA	Variant	tsRNA ID (MINTbase)	Sequence	Seq length	PCa_vs_BPH_pvalue	baseMean	BPH mean	PCa mean
Ala-AGC	3'-tRF	tRF-27-M2WEPSJR852	CGGGATCGATGCCCGCATCCTCCACCA	27	0,003484527	181,794674	96,5354311	210,214422
		tRF-28-QKHUXRPM46D2	GCGGGATCGATGCCCGCATCCTCCACCA	28	0,079956385	10,3491581	5,18054403	12,0720294
		tRF-29-F85SI1LQ3RE2	AGCGGGATCGATGCCCGCATCCTCCACCA	29	0,311742076	982,805212	1334,92779	865,431018
		tRF-30-VH6M5V3F8926	TAGCGGGATCGATGCCCGCATCCTCCACCA	30	0,57594837	902,834345	1162,90969	816,142564
		tRF-31-SJREL4KVH7K1B	GTAGCGGGATCGATGCCCGCATCCTCCACCA	31	0,006556761	31,5988807	15,6925867	36,9009787
		tRF-32-RPM2WEPSJR852	GGTAGCGGGATCGATGCCCGCATCCTCCACCA	32	0,220232703	20,9242521	14,5441051	23,0509677
		tRF-33-1LQKHUXRPM46D2	AGGTAGCGGGATCGATGCCCGCATCCTCCACCA	33	0,296769332	33,8169396	25,0685546	36,7330679
		tRF-34-53F85SI1LQ3RE2	GAGGTAGCGGGATCGATGCCCGCATCCTCCACCA	34	ND	ND	ND	ND
		tRF-35-FKVH6M5V3F8926	AGAGGTAGCGGGATCGATGCCCGCATCCTCCACCA	35	ND	ND	ND	ND
		tRF-37-L2RPM2WEPSJR852	CGAGAGGTAGCGGGATCGATGCCCGCATCCTCCACCA	37	ND	ND	ND	ND
		tRF-38-Q01LQKHUXRPM46D2	GCGAGAGGTAGCGGGATCGATGCCCGCATCCTCCACCA	38	ND	ND	ND	ND
		tRF-39-XU53F85SI1LQ3RE2	TGCGAGAGGTAGCGGGATCGATGCCCGCATCCTCCACCA	39	ND	ND	ND	ND
tRNA	Variant	tsRNA ID (MINTbase)	Sequence	Seq length	PCa_vs_BPH_pvalue	baseMean	BPH mean	PCa mean
Asp-GTC	i-tRF	tRF-37-8HM2OSRNLNKSEKL	TCACGCGGGAGACCGGGGTTCGATTCCCCGACGGGGA	37	0,004595801	1175,21022	1778,09836	974,247503
		tRF-38-8HM2OSRNLNKSEKDS	TCACGCGGGAGACCGGGGTTCGATTCCCCGACGGGGAG	38	0,000726874	4591,65647	7570,51613	3598,70325
		tRF-38-S5QKF1R3WE8RO8DX	GTCACGCGGGAGACCGGGGTTCGATTCCCCGACGGGGA	38	0,872752851	32,4310956	33,0310031	32,2311264
		tRF-39-8HM2OSRNLNKSEKH9	TCACGCGGGAGACCGGGGTTCGATTCCCCGACGGGGAGC	39	0,001892638	6119,79439	9992,16479	4829,00426
		tRF-39-96L85DMKYUYRLHIX	TGTCACGCGGGAGACCGGGGTTCGATTCCCCGACGGGGA	39	0,081829206	57,8512362	77,6824069	51,240846

tRF-39-S5QKF1R3WE8RO8IS	GTCACGCGGGAGACCGGGGTTCGATTCCCCGACGGGGAG	39	0,068422437	349,111886	471,037565	308,469993
tRF-40-96L85DMKYUYRLHR0	TGTCACGCGGGAGACCGGGGTTCGATTCCCCGACGGGGAG	40	0,012566653	418,023343	627,705427	348,129315
tRF-40-NMEH623K7SIR3DR2	CTGTCACGCGGGAGACCGGGGTTCGATTCCCCGACGGGGA	40	0,025638344	139,600212	196,983911	120,472312
tRF-40-S5QKF1R3WE8RO86J	GTCACGCGGGAGACCGGGGTTCGATTCCCCGACGGGGAGC	40	0,084157864	252,690667	328,906633	227,285344
tRF-41-3W2VR008R959KUMKB	CCTGTCACGCGGGAGACCGGGGTTCGATTCCCCGACGGGGA	41	0,012535552	120,041708	192,669408	95,8324741
tRF-41-96L85DMKYUYRLHR0D	TGTCACGCGGGAGACCGGGGTTCGATTCCCCGACGGGGAGC	41	0,056189716	340,462167	472,898659	296,316669
tRF-41-NMEH623K7SIR3DR20	CTGTCACGCGGGAGACCGGGGTTCGATTCCCCGACGGGGAG	41	0,014169053	1087,17183	1684,32315	888,121385
tRF-41-WW2VR008R959KUMKB	TCTGTCACGCGGGAGACCGGGGTTCGATTCCCCGACGGGGA	41	ND	ND	ND	ND
tRF-42-3W2VR008R959KUMKH	CCTGTCACGCGGGAGACCGGGGTTCGATTCCCCGACGGGGAG	42	0,101466668	1160,26898	1887,74958	917,775445
tRF-42-NMEH623K7SIR3DR2M	CTGTCACGCGGGAGACCGGGGTTCGATTCCCCGACGGGGAGC	42	0,041270916	619,297151	876,371226	533,605793
tRF-42-PY8HM2OSRNLNKSEKL	GCCTGTCACGCGGGAGACCGGGGTTCGATTCCCCGACGGGGA	42	0,017985864	156,256655	233,368303	130,552772
tRF-42-WW2VR008R959KUMKH	TCTGTCACGCGGGAGACCGGGGTTCGATTCCCCGACGGGGAG	42	ND	ND	ND	ND
tRF-43-3W2VR008R959KUMK9	CCTGTCACGCGGGAGACCGGGGTTCGATTCCCCGACGGGGAGC	43	0,034205101	321,348526	499,08031	262,104598
tRF-43-L7S5QKF1R3WE8RO8DX	CGCCTGTCACGCGGGAGACCGGGGTTCGATTCCCCGACGGGGA	43	0,032872115	169,51593	244,797493	144,422076
tRF-43-M7S5QKF1R3WE8RO8DX	CGTCTGTCACGCGGGAGACCGGGGTTCGATTCCCCGACGGGGA	43	ND	ND	ND	ND
tRF-43-PY8HM2OSRNLNKSEKDS	GCCTGTCACGCGGGAGACCGGGGTTCGATTCCCCGACGGGGAG	43	0,090686843	620,635217	835,841266	548,899868
tRF-43-SY8HM2OSRNLNKSEKDS	GTCTGTCACGCGGGAGACCGGGGTTCGATTCCCCGACGGGGAG	43	ND	ND	ND	ND
tRF-43-WW2VR008R959KUMK9	TCTGTCACGCGGGAGACCGGGGTTCGATTCCCCGACGGGGAGC	43	ND	ND	ND	ND
tRF-44-3196L85DMKYUYRLHIX	CCGCCTGTCACGCGGGAGACCGGGGTTCGATTCCCCGACGGGGA	44	0,090384163	40,891741	59,4670938	34,6999567
tRF-44-L7S5QKF1R3WE8RO8IS	CGCCTGTCACGCGGGAGACCGGGGTTCGATTCCCCGACGGGGAG	44	0,065198797	305,419702	419,012356	267,555483
tRF-44-M7S5QKF1R3WE8RO8IS	CGTCTGTCACGCGGGAGACCGGGGTTCGATTCCCCGACGGGGAG	44	ND	ND	ND	ND
tRF-44-PY8HM2OSRNLNKSEKH9	GCCTGTCACGCGGGAGACCGGGGTTCGATTCCCCGACGGGGAGC	44	0,106323079	541,781666	749,5434	472,527755
tRF-44-SY8HM2OSRNLNKSEKH9	GTCTGTCACGCGGGAGACCGGGGTTCGATTCCCCGACGGGGAGC	44	ND	ND	ND	ND
tRF-45-3196L85DMKYUYRLHR0	CCGCCTGTCACGCGGGAGACCGGGGTTCGATTCCCCGACGGGGAG	45	0,039398218	379,305793	556,854608	320,122854
tRF-45-3M96L85DMKYUYRLHR0	CCGTCTGTCACGCGGGAGACCGGGGTTCGATTCCCCGACGGGGAG	45	ND	ND	ND	ND
tRF-45-KVNMEH623K7SIR3DR2	CCCGCCTGTCACGCGGGAGACCGGGGTTCGATTCCCCGACGGGGA	45	0,159398508	10,9862464	15,9692555	9,32524338
tRF-45-L7S5QKF1R3WE8RO86J	CGCCTGTCACGCGGGAGACCGGGGTTCGATTCCCCGACGGGGAGC	45	0,157006924	182,44855	239,814137	163,326688
tRF-46-3196L85DMKYUYRLHR0D	CCGCCTGTCACGCGGGAGACCGGGGTTCGATTCCCCGACGGGGAGC	46	0,040658898	135,170553	204,575695	112,035505
tRF-46-KS3W2VR008R959KUMKB	CCCCGCCTGTCACGCGGGAGACCGGGGTTCGATTCCCCGACGGGGA	46	0,501126583	10,6414538	12,2682832	10,0991773
tRF-46-KVNMEH623K7SIR3DR20	CCCGCCTGTCACGCGGGAGACCGGGGTTCGATTCCCCGACGGGGAG	46	0,3598833	79,3410438	96,033412	73,7769211
tRF-47-8RPY8HM2OSRNLNKSEKL	TCCCCGCCTGTCACGCGGGAGACCGGGGTTCGATTCCCCGACGGGGA	47	ND	ND	ND	ND
tRF-47-KS3W2VR008R959KUMKH	CCCCGCCTGTCACGCGGGAGACCGGGGTTCGATTCCCCGACGGGGAG	47	0,203589694	180,264581	230,732927	163,441798
tRF-47-KVNMEH623K7SIR3DR2M	CCCGCCTGTCACGCGGGAGACCGGGGTTCGATTCCCCGACGGGGAGC	47	0,208913238	48,3394819	64,974739	42,7943961
tRF-47-KWNMEH623K7SIR3DR2M	CCCGTCTGTCACGCGGGAGACCGGGGTTCGATTCCCCGACGGGGAGC	47	ND	ND	ND	ND
tRF-48-8RPY8HM2OSRNLNKSEKDS	TCCCCGCCTGTCACGCGGGAGACCGGGGTTCGATTCCCCGACGGGGAG	48	0,633596422	42,4067707	48,9685185	40,2195214
tRF-48-HRL7S5QKF1R3WE8RO8DX	ATCCCCGCCTGTCACGCGGGAGACCGGGGTTCGATTCCCCGACGGGGA	48	0,816018157	9,9959924	10,6418562	9,78070447
tRF-48-KS3W2VR008R959KUMK9	CCCCGCCTGTCACGCGGGAGACCGGGGTTCGATTCCCCGACGGGGAGC	48	0,196053651	73,3450077	94,6568167	66,2410713
tRF-49-8RPY8HM2OSRNLNKSEKH9	TCCCCGCCTGTCACGCGGGAGACCGGGGTTCGATTCCCCGACGGGGAGC	49	0,109701403	52,7715643	76,70344	44,7942724
tRF-49-HRL7S5QKF1R3WE8RO8IS	ATCCCCGCCTGTCACGCGGGAGACCGGGGTTCGATTCCCCGACGGGGAG	49	0,331486887	31,7420894	40,4245665	28,8479303
tRF-49-VR3196L85DMKYUYRLHIX	TATCCCCGCCTGTCACGCGGGAGACCGGGGTTCGATTCCCCGACGGGGA	49	ND	ND	ND	ND
tRF-49-VR3196L85DMKYUYRLHIX	TATCCCCGCCTGTCACGCGGGAGACCGGGGTTCGATTCCCCGACGGGGA	49	ND	ND	ND	ND
tRF-50-HRL7S5QKF1R3WE8RO86J	ATCCCCGCCTGTCACGCGGGAGACCGGGGTTCGATTCCCCGACGGGGAGC	50	0,455021201	53,4530864	63,0867668	50,2418595
tRF-50-SMKVNMEH623K7SIR3DR2	GTATCCCCGCCTGTCACGCGGGAGACCGGGGTTCGATTCCCCGACGGGGA	50	ND	ND	ND	ND
tRF-50-VR3196L85DMKYUYRLHR0	TATCCCCGCCTGTCACGCGGGAGACCGGGGTTCGATTCCCCGACGGGGAG	50	0,781990171	10,2286309	11,6932393	9,74042807

tRNA	Variant	tsRNA ID (MINTbase)	Sequence	Seq length	PCa_vs_BPH_pvalue	baseMean	BPH mean	PCa mean
Asp-GTC	i-tRF	tRF-38-8HM2OSRNLNKSEKDS	TCACGCGGGAGACCGGGGTTCGATTCCCCGACGGGGAG	38	0,000726874	4591,65647	7570,51613	3598,70325
		tRF-39-8HM2OSRNLNKSEKH9	TCACGCGGGAGACCGGGGTTCGATTCCCCGACGGGGAGC	39	0,001892638	6119,79439	9992,16479	4829,00426
		tRF-39-S5QKF1R3WE8RO8IS	GTCACGCGGGAGACCGGGGTTCGATTCCCCGACGGGGAG	39	0,068422437	349,111886	471,037565	308,469993
		tRF-40-8HM2OSRNLNKSEK51	TCACGCGGGAGACCGGGGTTCGATTCCCCGACGGGGAGCC	40	0,001508443	10126,2544	16385,9575	8039,68671
		tRF-40-96L85DMKYUYRLHR0	TGTCACGCGGGAGACCGGGGTTCGATTCCCCGACGGGGAG	40	0,012566653	418,023343	627,705427	348,129315
		tRF-40-S5QKF1R3WE8RO86J	GTCACGCGGGAGACCGGGGTTCGATTCCCCGACGGGGAGC	40	0,084157864	252,690667	328,906633	227,285344
		tRF-41-96L85DMKYUYRLHR0D	TGTCACGCGGGAGACCGGGGTTCGATTCCCCGACGGGGAGC	41	0,056189716	340,462167	472,898659	296,316669
		tRF-41-NMEH623K7SIR3DR20	CTGTCACGCGGGAGACCGGGGTTCGATTCCCCGACGGGGAG	41	0,014169053	1087,17183	1684,32315	888,121385
		tRF-41-S5QKF1R3WE8RO86JD	GTCACGCGGGAGACCGGGGTTCGATTCCCCGACGGGGAGCC	41	0,025932413	439,093206	628,057856	376,104989
		tRF-42-3W2VR008R959KUMKH	CCTGTCACGCGGGAGACCGGGGTTCGATTCCCCGACGGGGAG	42	0,101466668	1160,26898	1887,74958	917,775445
		tRF-42-96L85DMKYUYRLHR0J	TGTCACGCGGGAGACCGGGGTTCGATTCCCCGACGGGGAGCC	42	0,021051174	581,942591	817,793986	503,325459
		tRF-42-NMEH623K7SIR3DR2M	CTGTCACGCGGGAGACCGGGGTTCGATTCCCCGACGGGGAGC	42	0,041270916	619,297151	876,371226	533,605793
		tRF-42-WW2VR008R959KUMKH	TCTGTCACGCGGGAGACCGGGGTTCGATTCCCCGACGGGGAG	42	ND	ND	ND	ND
		tRF-43-3W2VR008R959KUMK9	CCTGTCACGCGGGAGACCGGGGTTCGATTCCCCGACGGGGAGC	43	0,034205101	321,348526	499,08031	262,104598
		tRF-43-NMEH623K7SIR3DR2DV	CTGTCACGCGGGAGACCGGGGTTCGATTCCCCGACGGGGAGCC	43	0,025162545	1128,09041	1650,83691	953,841573
		tRF-43-PY8HM2OSRNLNKSEKDS	GCCTGTCACGCGGGAGACCGGGGTTCGATTCCCCGACGGGGAG	43	0,090686843	620,635217	835,841266	548,899868
		tRF-43-SY8HM2OSRNLNKSEKDS	GTCTGTCACGCGGGAGACCGGGGTTCGATTCCCCGACGGGGAG	43	ND	ND	ND	ND
		tRF-43-WW2VR008R959KUMK9	TCTGTCACGCGGGAGACCGGGGTTCGATTCCCCGACGGGGAGC	43	ND	ND	ND	ND
		tRF-44-3W2VR008R959KUMKEV	CCTGTCACGCGGGAGACCGGGGTTCGATTCCCCGACGGGGAGCC	44	0,033336299	855,244196	1261,88379	719,697666
		tRF-44-L7S5QKF1R3WE8RO8IS	CGCCTGTCACGCGGGAGACCGGGGTTCGATTCCCCGACGGGGAG	44	0,065198797	305,419702	419,012356	267,555483
		tRF-44-M7S5QKF1R3WE8RO8IS	CGTCTGTCACGCGGGAGACCGGGGTTCGATTCCCCGACGGGGAG	44	ND	ND	ND	ND
		tRF-44-PY8HM2OSRNLNKSEKH9	GCCTGTCACGCGGGAGACCGGGGTTCGATTCCCCGACGGGGAGC	44	0,106323079	541,781666	749,5434	472,527755
		tRF-44-SY8HM2OSRNLNKSEKH9	GTCTGTCACGCGGGAGACCGGGGTTCGATTCCCCGACGGGGAGC	44	ND	ND	ND	ND
		tRF-44-WW2VR008R959KUMKEV	TCTGTCACGCGGGAGACCGGGGTTCGATTCCCCGACGGGGAGCC	44	ND	ND	ND	ND
		tRF-45-3196L85DMKYUYRLHR0	CCGCCTGTCACGCGGGAGACCGGGGTTCGATTCCCCGACGGGGAG	45	0,039398218	379,305793	556,854608	320,122854
		tRF-45-3M96L85DMKYUYRLHR0	CCGTCTGTCACGCGGGAGACCGGGGTTCGATTCCCCGACGGGGAG	45	ND	ND	ND	ND
		tRF-45-L7S5QKF1R3WE8RO86J	CGCCTGTCACGCGGGAGACCGGGGTTCGATTCCCCGACGGGGAGC	45	0,157006924	182,44855	239,814137	163,326688
		tRF-45-PY8HM2OSRNLNKSEK51	GCCTGTCACGCGGGAGACCGGGGTTCGATTCCCCGACGGGGAGCC	45	0,06603806	1533,83682	2165,19119	1323,38536
		tRF-45-SY8HM2OSRNLNKSEK51	GTCTGTCACGCGGGAGACCGGGGTTCGATTCCCCGACGGGGAGCC	45	ND	ND	ND	ND
		tRF-46-3196L85DMKYUYRLHR0D	CCGCCTGTCACGCGGGAGACCGGGGTTCGATTCCCCGACGGGGAGC	46	0,040658898	135,170553	204,575695	112,035505
		tRF-46-KVNMEH623K7SIR3DR20	CCCGCCTGTCACGCGGGAGACCGGGGTTCGATTCCCCGACGGGGAG	46	0,3598833	79,3410438	96,033412	73,7769211
		tRF-46-L7S5QKF1R3WE8RO86JD	CGCCTGTCACGCGGGAGACCGGGGTTCGATTCCCCGACGGGGAGCC	46	0,049062725	271,877387	397,907637	229,867304
		tRF-46-M7S5QKF1R3WE8RO86JD	CGTCTGTCACGCGGGAGACCGGGGTTCGATTCCCCGACGGGGAGCC	46	ND	ND	ND	ND
		tRF-47-3196L85DMKYUYRLHR0J	CCGCCTGTCACGCGGGAGACCGGGGTTCGATTCCCCGACGGGGAGCC	47	0,024924742	404,887912	631,502681	329,349655
		tRF-47-KS3W2VR008R959KUMKH	CCCCGCCTGTCACGCGGGAGACCGGGGTTCGATTCCCCGACGGGGAG	47	0,203589694	180,264581	230,732927	163,441798
		tRF-47-KVNMEH623K7SIR3DR2M	CCCGCCTGTCACGCGGGAGACCGGGGTTCGATTCCCCGACGGGGAGC	47	0,208913238	48,3394819	64,974739	42,7943961
		tRF-47-KWNMEH623K7SIR3DR2M	CCCGTCTGTCACGCGGGAGACCGGGGTTCGATTCCCCGACGGGGAGC	47	ND	ND	ND	ND
		tRF-48-8RPY8HM2OSRNLNKSEKDS	TCCCCGCCTGTCACGCGGGAGACCGGGGTTCGATTCCCCGACGGGGAG	48	0,633596422	42,4067707	48,9685185	40,2195214
		tRF-48-KS3W2VR008R959KUMK9	CCCCGCCTGTCACGCGGGAGACCGGGGTTCGATTCCCCGACGGGGAGC	48	0,196053651	73,3450077	94,6568167	66,2410713
		tRF-48-KVNMEH623K7SIR3DR2DV	CCCGCCTGTCACGCGGGAGACCGGGGTTCGATTCCCCGACGGGGAGCC	48	0,2931794	145,235293	181,400865	133,180103
		tRF-49-8RPY8HM2OSRNLNKSEKH9	TCCCCGCCTGTCACGCGGGAGACCGGGGTTCGATTCCCCGACGGGGAGC	49	0,109701403	52,7715643	76,70344	44,7942724
		tRF-49-HRL7S5QKF1R3WE8RO8IS	ATCCCCGCCTGTCACGCGGGAGACCGGGGTTCGATTCCCCGACGGGGAG	49	0,331486887	31,7420894	40,4245665	28,8479303
		tRF-49-KS3W2VR008R959KUMKEV	CCCCGCCTGTCACGCGGGAGACCGGGGTTCGATTCCCCGACGGGGAGCC	49	0,048594852	395,52904	586,345529	331,923543
		tRF-50-8RPY8HM2OSRNLNKSEK51	TCCCCGCCTGTCACGCGGGAGACCGGGGTTCGATTCCCCGACGGGGAGCC	50	0,119514471	131,457434	180,344264	115,161824

		tRF-50-8RSY8HM2OSRNLNKSEK51	TCCCCGTCTGTCACGCGGGAGACCGGGGTTCGATTCCCCGACGGGGAGCC	50	ND	ND	ND	ND
		tRF-50-HRL7S5QKF1R3WE8RO86J	ATCCCCGCCTGTCACGCGGGAGACCGGGGTTCGATTCCCCGACGGGGAGC	50	0,455021201	53,4530864	63,0867668	50,2418595
		tRF-50-VR3196L85DMKYUYRLHR0	TATCCCCGCCTGTCACGCGGGAGACCGGGGTTCGATTCCCCGACGGGGAG	50	0,781990171	10,2286309	11,6932393	9,74042807

tRNA	Variant	tsRNA ID (MINTbase)	Sequence	Seq length	PCa_vs_BPH_pvalue	baseMean	BPH mean	PCa mean
Asp-GTC	3'-half	tRF-39-8HM2OSRNLNKSEKH9	TCACGCGGGAGACCGGGGTTCGATTCCCCGACGGGGAGC	39	0,001892638	6119,79439	9992,16479	4829,00426
		tRF-40-8HM2OSRNLNKSEK51	TCACGCGGGAGACCGGGGTTCGATTCCCCGACGGGGAGCC	40	0,001508443	10126,2544	16385,9575	8039,68671
		tRF-40-S5QKF1R3WE8RO86J	GTCACGCGGGAGACCGGGGTTCGATTCCCCGACGGGGAGC	40	0,084157864	252,690667	328,906633	227,285344
		tRF-41-8HM2OSRNLNKSEK51B	TCACGCGGGAGACCGGGGTTCGATTCCCCGACGGGGAGCCA	41	0,001363246	40599,0536	63718,5897	32892,5415
		tRF-41-96L85DMKYUYRLHR0D	TGTCACGCGGGAGACCGGGGTTCGATTCCCCGACGGGGAGC	41	0,056189716	340,462167	472,898659	296,316669
		tRF-41-S5QKF1R3WE8RO86JD	GTCACGCGGGAGACCGGGGTTCGATTCCCCGACGGGGAGCC	41	0,025932413	439,093206	628,057856	376,104989
		tRF-42-96L85DMKYUYRLHR0J	TGTCACGCGGGAGACCGGGGTTCGATTCCCCGACGGGGAGCC	42	0,021051174	581,942591	817,793986	503,325459
		tRF-42-NMEH623K7SIR3DR2M	CTGTCACGCGGGAGACCGGGGTTCGATTCCCCGACGGGGAGC	42	0,041270916	619,297151	876,371226	533,605793
		tRF-42-S5QKF1R3WE8RO86J2	GTCACGCGGGAGACCGGGGTTCGATTCCCCGACGGGGAGCCA	42	0,080889154	2501,30568	3239,07993	2255,38093
		tRF-43-3W2VR008R959KUMK9	CCTGTCACGCGGGAGACCGGGGTTCGATTCCCCGACGGGGAGC	43	0,034205101	321,348526	499,08031	262,104598
		tRF-43-96L85DMKYUYRLHR0D2	TGTCACGCGGGAGACCGGGGTTCGATTCCCCGACGGGGAGCCA	43	0,028287805	2336,42834	3184,00274	2053,90354
		tRF-43-NMEH623K7SIR3DR2DV	CTGTCACGCGGGAGACCGGGGTTCGATTCCCCGACGGGGAGCC	43	0,025162545	1128,09041	1650,83691	953,841573
		tRF-43-WW2VR008R959KUMK9	TCTGTCACGCGGGAGACCGGGGTTCGATTCCCCGACGGGGAGC	43	ND	ND	ND	ND
		tRF-44-3W2VR008R959KUMKEV	CCTGTCACGCGGGAGACCGGGGTTCGATTCCCCGACGGGGAGCC	44	0,033336299	855,244196	1261,88379	719,697666
		tRF-44-NMEH623K7SIR3DR2I2	CTGTCACGCGGGAGACCGGGGTTCGATTCCCCGACGGGGAGCCA	44	0,034854092	6531,36398	9070,61392	5684,94734
		tRF-44-PY8HM2OSRNLNKSEKH9	GCCTGTCACGCGGGAGACCGGGGTTCGATTCCCCGACGGGGAGC	44	0,106323079	541,781666	749,5434	472,527755
		tRF-44-SY8HM2OSRNLNKSEKH9	GTCTGTCACGCGGGAGACCGGGGTTCGATTCCCCGACGGGGAGC	44	ND	ND	ND	ND
		tRF-44-WW2VR008R959KUMKEV	TCTGTCACGCGGGAGACCGGGGTTCGATTCCCCGACGGGGAGCC	44	ND	ND	ND	ND
		tRF-45-3W2VR008R959KUMKF6	CCTGTCACGCGGGAGACCGGGGTTCGATTCCCCGACGGGGAGCCA	45	0,02436288	8024,84073	12727,3508	6457,33737
		tRF-45-L7S5QKF1R3WE8RO86J	CGCCTGTCACGCGGGAGACCGGGGTTCGATTCCCCGACGGGGAGC	45	0,157006924	182,44855	239,814137	163,326688
		tRF-45-PY8HM2OSRNLNKSEK51	GCCTGTCACGCGGGAGACCGGGGTTCGATTCCCCGACGGGGAGCC	45	0,06603806	1533,83682	2165,19119	1323,38536
		tRF-45-SY8HM2OSRNLNKSEK51	GTCTGTCACGCGGGAGACCGGGGTTCGATTCCCCGACGGGGAGCC	45	ND	ND	ND	ND
		tRF-45-WW2VR008R959KUMKF6	TCTGTCACGCGGGAGACCGGGGTTCGATTCCCCGACGGGGAGCCA	45	0,022626877	30,7995647	50,3645973	24,2778872
		tRF-46-3196L85DMKYUYRLHR0D	CCGCCTGTCACGCGGGAGACCGGGGTTCGATTCCCCGACGGGGAGC	46	0,040658898	135,170553	204,575695	112,035505
		tRF-46-L7S5QKF1R3WE8RO86JD	CGCCTGTCACGCGGGAGACCGGGGTTCGATTCCCCGACGGGGAGCC	46	0,049062725	271,877387	397,907637	229,867304
		tRF-46-M7S5QKF1R3WE8RO86JD	CGTCTGTCACGCGGGAGACCGGGGTTCGATTCCCCGACGGGGAGCC	46	ND	ND	ND	ND
		tRF-46-PY8HM2OSRNLNKSEK51B	GCCTGTCACGCGGGAGACCGGGGTTCGATTCCCCGACGGGGAGCCA	46	0,062444924	6296,95805	8527,53783	5553,43145
		tRF-46-SY8HM2OSRNLNKSEK51B	GTCTGTCACGCGGGAGACCGGGGTTCGATTCCCCGACGGGGAGCCA	46	ND	ND	ND	ND
		tRF-47-3196L85DMKYUYRLHR0J	CCGCCTGTCACGCGGGAGACCGGGGTTCGATTCCCCGACGGGGAGCC	47	0,024924742	404,887912	631,502681	329,349655
		tRF-47-KVNMEH623K7SIR3DR2M	CCCGCCTGTCACGCGGGAGACCGGGGTTCGATTCCCCGACGGGGAGC	47	0,208913238	48,3394819	64,974739	42,7943961
		tRF-47-KWNMEH623K7SIR3DR2M	CCCGTCTGTCACGCGGGAGACCGGGGTTCGATTCCCCGACGGGGAGC	47	ND	ND	ND	ND
		tRF-47-L7S5QKF1R3WE8RO86J2	CGCCTGTCACGCGGGAGACCGGGGTTCGATTCCCCGACGGGGAGCCA	47	0,071093769	4600,0464	6165,43342	4078,25072
		tRF-47-M7S5QKF1R3WE8RO86J2	CGTCTGTCACGCGGGAGACCGGGGTTCGATTCCCCGACGGGGAGCCA	47	ND	ND	ND	ND
		tRF-48-3196L85DMKYUYRLHR0D2	CCGCCTGTCACGCGGGAGACCGGGGTTCGATTCCCCGACGGGGAGCCA	48	0,029369619	3689,18503	5375,89741	3126,94757
		tRF-48-3M96L85DMKYUYRLHR0D2	CCGTCTGTCACGCGGGAGACCGGGGTTCGATTCCCCGACGGGGAGCCA	48	ND	ND	ND	ND
		tRF-48-KS3W2VR008R959KUMK9	CCCCGCCTGTCACGCGGGAGACCGGGGTTCGATTCCCCGACGGGGAGC	48	0,196053651	73,3450077	94,6568167	66,2410713
		tRF-48-KVNMEH623K7SIR3DR2DV	CCCGCCTGTCACGCGGGAGACCGGGGTTCGATTCCCCGACGGGGAGCC	48	0,2931794	145,235293	181,400865	133,180103
		tRF-49-8RPY8HM2OSRNLNKSEKH9	TCCCCGCCTGTCACGCGGGAGACCGGGGTTCGATTCCCCGACGGGGAGC	49	0,109701403	52,7715643	76,70344	44,7942724
		tRF-49-KS3W2VR008R959KUMKEV	CCCCGCCTGTCACGCGGGAGACCGGGGTTCGATTCCCCGACGGGGAGCC	49	0,048594852	395,52904	586,345529	331,923543
		tRF-49-KVNMEH623K7SIR3DR2I2	CCCGCCTGTCACGCGGGAGACCGGGGTTCGATTCCCCGACGGGGAGCCA	49	0,056111452	1139,29472	1519,17311	1012,66859
		tRF-50-8RPY8HM2OSRNLNKSEK51	TCCCCGCCTGTCACGCGGGAGACCGGGGTTCGATTCCCCGACGGGGAGCC	50	0,119514471	131,457434	180,344264	115,161824

		tRF-50-8RSY8HM2OSRNLNKSEK51	TCCCCGTCTGTCACGCGGGAGACCGGGGTTCGATTCCCCGACGGGGAGCC	50	ND	ND	ND	ND
		tRF-50-HRL7S5QKF1R3WE8RO86J	ATCCCCGCCTGTCACGCGGGAGACCGGGGTTCGATTCCCCGACGGGGAGC	50	0,455021201	53,4530864	63,0867668	50,2418595
		tRF-50-KS3W2VR008R959KUMKF6	CCCCGCCTGTCACGCGGGAGACCGGGGTTCGATTCCCCGACGGGGAGCCA	50	0,22653777	1803,82573	2208,72163	1668,86044

tRNA	Variant	tsRNA ID (MINTbase)	Sequence	Seq length	PCa_vs_BPH_pvalue	baseMean	BPH mean	PCa mean
Asp-GTC	3'-half	tRF-40-8HM2OSRNLNKSEK51	TCACGCGGGAGACCGGGGTTCGATTCCCCGACGGGGAGCC	40	0,001508443	10126,2544	16385,9575	8039,68671
		tRF-41-8HM2OSRNLNKSEK51B	TCACGCGGGAGACCGGGGTTCGATTCCCCGACGGGGAGCCA	41	0,001363246	40599,0536	63718,5897	32892,5415
		tRF-41-S5QKF1R3WE8RO86JD	GTCACGCGGGAGACCGGGGTTCGATTCCCCGACGGGGAGCC	41	0,025932413	439,093206	628,057856	376,104989
		tRF-42-96L85DMKYUYRLHR0J	TGTCACGCGGGAGACCGGGGTTCGATTCCCCGACGGGGAGCC	42	0,021051174	581,942591	817,793986	503,325459
		tRF-42-S5QKF1R3WE8RO86J2	GTCACGCGGGAGACCGGGGTTCGATTCCCCGACGGGGAGCCA	42	0,080889154	2501,30568	3239,07993	2255,38093
		tRF-43-96L85DMKYUYRLHR0D2	TGTCACGCGGGAGACCGGGGTTCGATTCCCCGACGGGGAGCCA	43	0,028287805	2336,42834	3184,00274	2053,90354
		tRF-43-NMEH623K7SIR3DR2DV	CTGTCACGCGGGAGACCGGGGTTCGATTCCCCGACGGGGAGCC	43	0,025162545	1128,09041	1650,83691	953,841573
		tRF-44-3W2VR008R959KUMKEV	CCTGTCACGCGGGAGACCGGGGTTCGATTCCCCGACGGGGAGCC	44	0,033336299	855,244196	1261,88379	719,697666
		tRF-44-NMEH623K7SIR3DR2I2	CTGTCACGCGGGAGACCGGGGTTCGATTCCCCGACGGGGAGCCA	44	0,034854092	6531,36398	9070,61392	5684,94734
		tRF-44-WW2VR008R959KUMKEV	TCTGTCACGCGGGAGACCGGGGTTCGATTCCCCGACGGGGAGCC	44	ND	ND	ND	ND
		tRF-45-3W2VR008R959KUMKF6	CCTGTCACGCGGGAGACCGGGGTTCGATTCCCCGACGGGGAGCCA	45	0,02436288	8024,84073	12727,3508	6457,33737
		tRF-45-PY8HM2OSRNLNKSEK51	GCCTGTCACGCGGGAGACCGGGGTTCGATTCCCCGACGGGGAGCC	45	0,06603806	1533,83682	2165,19119	1323,38536
		tRF-45-SY8HM2OSRNLNKSEK51	GTCTGTCACGCGGGAGACCGGGGTTCGATTCCCCGACGGGGAGCC	45	ND	ND	ND	ND
		tRF-45-WW2VR008R959KUMKF6	TCTGTCACGCGGGAGACCGGGGTTCGATTCCCCGACGGGGAGCCA	45	0,022626877	30,7995647	50,3645973	24,2778872
		tRF-46-L7S5QKF1R3WE8RO86JD	CGCCTGTCACGCGGGAGACCGGGGTTCGATTCCCCGACGGGGAGCC	46	0,049062725	271,877387	397,907637	229,867304
		tRF-46-M7S5QKF1R3WE8RO86JD	CGTCTGTCACGCGGGAGACCGGGGTTCGATTCCCCGACGGGGAGCC	46	ND	ND	ND	ND
		tRF-46-PY8HM2OSRNLNKSEK51B	GCCTGTCACGCGGGAGACCGGGGTTCGATTCCCCGACGGGGAGCCA	46	0,062444924	6296,95805	8527,53783	5553,43145
		tRF-46-SY8HM2OSRNLNKSEK51B	GTCTGTCACGCGGGAGACCGGGGTTCGATTCCCCGACGGGGAGCCA	46	ND	ND	ND	ND
		tRF-47-3196L85DMKYUYRLHR0J	CCGCCTGTCACGCGGGAGACCGGGGTTCGATTCCCCGACGGGGAGCC	47	0,024924742	404,887912	631,502681	329,349655
		tRF-47-L7S5QKF1R3WE8RO86J2	CGCCTGTCACGCGGGAGACCGGGGTTCGATTCCCCGACGGGGAGCCA	47	0,071093769	4600,0464	6165,43342	4078,25072
		tRF-47-M7S5QKF1R3WE8RO86J2	CGTCTGTCACGCGGGAGACCGGGGTTCGATTCCCCGACGGGGAGCCA	47	ND	ND	ND	ND
		tRF-48-3196L85DMKYUYRLHR0D2	CCGCCTGTCACGCGGGAGACCGGGGTTCGATTCCCCGACGGGGAGCCA	48	0,029369619	3689,18503	5375,89741	3126,94757
		tRF-48-3M96L85DMKYUYRLHR0D2	CCGTCTGTCACGCGGGAGACCGGGGTTCGATTCCCCGACGGGGAGCCA	48	ND	ND	ND	ND
		tRF-48-KVNMEH623K7SIR3DR2DV	CCCGCCTGTCACGCGGGAGACCGGGGTTCGATTCCCCGACGGGGAGCC	48	0,2931794	145,235293	181,400865	133,180103
		tRF-49-KS3W2VR008R959KUMKEV	CCCCGCCTGTCACGCGGGAGACCGGGGTTCGATTCCCCGACGGGGAGCC	49	0,048594852	395,52904	586,345529	331,923543
		tRF-49-KVNMEH623K7SIR3DR2I2	CCCGCCTGTCACGCGGGAGACCGGGGTTCGATTCCCCGACGGGGAGCCA	49	0,056111452	1139,29472	1519,17311	1012,66859
		tRF-50-8RPY8HM2OSRNLNKSEK51	TCCCCGCCTGTCACGCGGGAGACCGGGGTTCGATTCCCCGACGGGGAGCC	50	0,119514471	131,457434	180,344264	115,161824
		tRF-50-8RSY8HM2OSRNLNKSEK51	TCCCCGTCTGTCACGCGGGAGACCGGGGTTCGATTCCCCGACGGGGAGCC	50	ND	ND	ND	ND
		tRF-50-KS3W2VR008R959KUMKF6	CCCCGCCTGTCACGCGGGAGACCGGGGTTCGATTCCCCGACGGGGAGCCA	50	0,22653777	1803,82573	2208,72163	1668,86044

tRNA	Variant	tsRNA ID (MINTbase)	Sequence	Seq length	PCa_vs_BPH_pvalue	baseMean	BPH mean	PCa mean
Asp-GTC	3'-half	tRF-41-8HM2OSRNLNKSEK51B	TCACGCGGGAGACCGGGGTTCGATTCCCCGACGGGGAGCCA	41	0,001363246	40599,0536	63718,5897	32892,5415
		tRF-45-3W2VR008R959KUMKF6	CCTGTCACGCGGGAGACCGGGGTTCGATTCCCCGACGGGGAGCCA	45	0,02436288	8024,84073	12727,3508	6457,33737
		tRF-44-NMEH623K7SIR3DR2I2	CTGTCACGCGGGAGACCGGGGTTCGATTCCCCGACGGGGAGCCA	44	0,034854092	6531,36398	9070,61392	5684,94734
		tRF-46-PY8HM2OSRNLNKSEK51B	GCCTGTCACGCGGGAGACCGGGGTTCGATTCCCCGACGGGGAGCCA	46	0,062444924	6296,95805	8527,53783	5553,43145
		tRF-47-L7S5QKF1R3WE8RO86J2	CGCCTGTCACGCGGGAGACCGGGGTTCGATTCCCCGACGGGGAGCCA	47	0,071093769	4600,0464	6165,43342	4078,25072
		tRF-48-3196L85DMKYUYRLHR0D2	CCGCCTGTCACGCGGGAGACCGGGGTTCGATTCCCCGACGGGGAGCCA	48	0,029369619	3689,18503	5375,89741	3126,94757
		tRF-42-S5QKF1R3WE8RO86J2	GTCACGCGGGAGACCGGGGTTCGATTCCCCGACGGGGAGCCA	42	0,080889154	2501,30568	3239,07993	2255,38093
		tRF-43-96L85DMKYUYRLHR0D2	TGTCACGCGGGAGACCGGGGTTCGATTCCCCGACGGGGAGCCA	43	0,028287805	2336,42834	3184,00274	2053,90354
		tRF-50-KS3W2VR008R959KUMKF6	CCCCGCCTGTCACGCGGGAGACCGGGGTTCGATTCCCCGACGGGGAGCCA	50	0,22653777	1803,82573	2208,72163	1668,86044
		tRF-49-KVNMEH623K7SIR3DR2I2	CCCGCCTGTCACGCGGGAGACCGGGGTTCGATTCCCCGACGGGGAGCCA	49	0,056111452	1139,29472	1519,17311	1012,66859
		tRF-45-WW2VR008R959KUMKF6	TCTGTCACGCGGGAGACCGGGGTTCGATTCCCCGACGGGGAGCCA	45	0,022626877	30,7995647	50,3645973	24,2778872

		tRF-48-3M96L85DMKYUYRLHR0D2	CCGTCTGTCACGCGGGAGACCGGGGTTCGATTCCCCGACGGGGAGCCA	48	ND	ND	ND	ND
		tRF-46-SY8HM2OSRNLNKSEK51B	GTCTGTCACGCGGGAGACCGGGGTTCGATTCCCCGACGGGGAGCCA	46	ND	ND	ND	ND
		tRF-47-M7S5QKF1R3WE8RO86J2	CGTCTGTCACGCGGGAGACCGGGGTTCGATTCCCCGACGGGGAGCCA	47	ND	ND	ND	ND

tRNA	Variant	tsRNA ID (MINTbase)	Sequence	Seq length	PCa_vs_BPH_pvalue	baseMean	BPH mean	PCa mean
Asp-GTC	i-tRF	tRF-38-8HM2OSRN2NKSEKDS	TCACGCGGGAGACCGGGGTTCAATTCCCCGACGGGGAG	38	0,000972009	822,391044	1393,31049	632,084562
		tRF-39-8HM2OSRN2NKSEKH9	TCACGCGGGAGACCGGGGTTCAATTCCCCGACGGGGAGC	39	0,01229359	804,87467	1231,02189	662,825595
		tRF-39-S5QKF1R38E8RO8IS	GTCACGCGGGAGACCGGGGTTCAATTCCCCGACGGGGAG	39	0,075111488	62,8506797	92,9951475	52,8025238
		tRF-40-8HM2OSRN2NKSEK51	TCACGCGGGAGACCGGGGTTCAATTCCCCGACGGGGAGCC	40	0,003353302	1470,10996	2319,01199	1187,14262
		tRF-40-96L85DMKYOYRLHR0	TGTCACGCGGGAGACCGGGGTTCAATTCCCCGACGGGGAG	40	0,113132641	52,2332097	71,1561831	45,9255519
		tRF-40-S5QKF1R38E8RO86J	GTCACGCGGGAGACCGGGGTTCAATTCCCCGACGGGGAGC	40	0,081715785	61,2209136	86,7133537	52,7234336
		tRF-41-8HM2OSRN2NKSEK51B	TCACGCGGGAGACCGGGGTTCAATTCCCCGACGGGGAGCCA	41	0,00099071	7786,16879	12374,0054	6256,88993
		tRF-41-96L85DMKYOYRLHR0D	TGTCACGCGGGAGACCGGGGTTCAATTCCCCGACGGGGAGC	41	0,39840753	44,4424672	54,2472531	41,1742052
		tRF-41-NMEH623K76IR3DR20	CTGTCACGCGGGAGACCGGGGTTCAATTCCCCGACGGGGAG	41	0,03797003	138,622912	207,408106	115,694514
		tRF-41-S5QKF1R38E8RO86JD	GTCACGCGGGAGACCGGGGTTCAATTCCCCGACGGGGAGCC	41	0,12491749	100,2161	136,818144	88,0154182
		tRF-42-3W2VR008R9D9KUMKH	CCTGTCACGCGGGAGACCGGGGTTCAATTCCCCGACGGGGAG	42	0,05168581	65,9237174	101,848442	53,9488093
		tRF-42-96L85DMKYOYRLHR0J	TGTCACGCGGGAGACCGGGGTTCAATTCCCCGACGGGGAGCC	42	0,108612668	87,8951927	118,344823	77,745316
		tRF-42-NMEH623K76IR3DR2M	CTGTCACGCGGGAGACCGGGGTTCAATTCCCCGACGGGGAGC	42	0,177770021	74,924502	99,0649491	66,8776864
		tRF-42-S5QKF1R38E8RO86J2	GTCACGCGGGAGACCGGGGTTCAATTCCCCGACGGGGAGCCA	42	0,233029438	416,055461	511,28537	384,312159
		tRF-43-3W2VR008R9D9KUMK9	CCTGTCACGCGGGAGACCGGGGTTCAATTCCCCGACGGGGAGC	43	0,173679498	24,3731431	35,0671385	20,808478
		tRF-43-96L85DMKYOYRLHR0D2	TGTCACGCGGGAGACCGGGGTTCAATTCCCCGACGGGGAGCCA	43	0,06808648	374,207029	484,572322	337,418598
		tRF-43-NMEH623K76IR3DR2DV	CTGTCACGCGGGAGACCGGGGTTCAATTCCCCGACGGGGAGCC	43	0,0627141	139,333546	191,18656	122,049208
		tRF-43-PY8HM2OSRN2NKSEKDS	GCCTGTCACGCGGGAGACCGGGGTTCAATTCCCCGACGGGGAG	43	0,267430162	82,2640534	104,456731	74,8664943
		tRF-44-3W2VR008R9D9KUMKEV	CCTGTCACGCGGGAGACCGGGGTTCAATTCCCCGACGGGGAGCC	44	0,2081577	57,0944839	75,1522411	51,0752316
		tRF-44-L7S5QKF1R38E8RO8IS	CGCCTGTCACGCGGGAGACCGGGGTTCAATTCCCCGACGGGGAG	44	0,100271602	34,8778566	48,8971583	30,204756
		tRF-44-NMEH623K76IR3DR2I2	CTGTCACGCGGGAGACCGGGGTTCAATTCCCCGACGGGGAGCCA	44	0,042968156	949,708407	1278,75129	840,027445
		tRF-44-PY8HM2OSRN2NKSEKH9	GCCTGTCACGCGGGAGACCGGGGTTCAATTCCCCGACGGGGAGC	44	0,093505519	71,1083119	100,504629	61,3095397
		tRF-45-3196L85DMKYOYRLHR0	CCGCCTGTCACGCGGGAGACCGGGGTTCAATTCCCCGACGGGGAG	45	0,582288865	44,261571	50,446634	42,1998833
		tRF-45-3W2VR008R9D9KUMKF6	CCTGTCACGCGGGAGACCGGGGTTCAATTCCCCGACGGGGAGCCA	45	0,034470265	829,353411	1172,68301	714,910211
		tRF-45-L7S5QKF1R38E8RO86J	CGCCTGTCACGCGGGAGACCGGGGTTCAATTCCCCGACGGGGAGC	45	0,310024207	23,209286	28,6454876	21,3972188
		tRF-45-PY8HM2OSRN2NKSEK51	GCCTGTCACGCGGGAGACCGGGGTTCAATTCCCCGACGGGGAGCC	45	0,198641346	184,467772	241,051398	165,606563
		tRF-46-3196L85DMKYOYRLHR0D	CCGCCTGTCACGCGGGAGACCGGGGTTCAATTCCCCGACGGGGAGC	46	0,82598373	13,7731501	14,1287458	13,6546183
		tRF-46-KVNMEH623K76IR3DR20	CCCGCCTGTCACGCGGGAGACCGGGGTTCAATTCCCCGACGGGGAG	46	0,225450424	19,3546679	26,2770046	17,0472223
		tRF-46-L7S5QKF1R38E8RO86JD	CGCCTGTCACGCGGGAGACCGGGGTTCAATTCCCCGACGGGGAGCC	46	0,218786371	27,2039127	36,2126396	24,2010037
		tRF-46-PY8HM2OSRN2NKSEK51B	GCCTGTCACGCGGGAGACCGGGGTTCAATTCCCCGACGGGGAGCCA	46	0,083981876	954,905858	1254,3434	855,093342
		tRF-47-3196L85DMKYOYRLHR0J	CCGCCTGTCACGCGGGAGACCGGGGTTCAATTCCCCGACGGGGAGCC	47	0,073455211	42,9157207	60,6486904	37,0047309
		tRF-47-KS3W2VR008R9D9KUMKH	CCCCGCCTGTCACGCGGGAGACCGGGGTTCAATTCCCCGACGGGGAG	47	0,543464583	12,8268923	15,6580304	11,8831796
		tRF-47-KVNMEH623K76IR3DR2M	CCCGCCTGTCACGCGGGAGACCGGGGTTCAATTCCCCGACGGGGAGC	47	ND	ND	ND	ND
		tRF-47-L7S5QKF1R38E8RO86J2	CGCCTGTCACGCGGGAGACCGGGGTTCAATTCCCCGACGGGGAGCCA	47	0,116895052	634,817229	818,023082	573,748611
		tRF-48-3196L85DMKYOYRLHR0D2	CCGCCTGTCACGCGGGAGACCGGGGTTCAATTCCCCGACGGGGAGCCA	48	0,038245798	535,934567	770,442752	457,765171
		tRF-48-8RPY8HM2OSRN2NKSEKDS	TCCCCGCCTGTCACGCGGGAGACCGGGGTTCAATTCCCCGACGGGGAG	48	ND	ND	ND	ND
		tRF-48-KS3W2VR008R9D9KUMK9	CCCCGCCTGTCACGCGGGAGACCGGGGTTCAATTCCCCGACGGGGAGC	48	ND	ND	ND	ND
		tRF-48-KVNMEH623K76IR3DR2DV	CCCGCCTGTCACGCGGGAGACCGGGGTTCAATTCCCCGACGGGGAGCC	48	0,133414842	9,01748589	13,7012395	7,4562347
		tRF-49-8RPY8HM2OSRN2NKSEKH9	TCCCCGCCTGTCACGCGGGAGACCGGGGTTCAATTCCCCGACGGGGAGC	49	ND	ND	ND	ND
		tRF-49-HRL7S5QKF1R38E8RO8IS	ATCCCCGCCTGTCACGCGGGAGACCGGGGTTCAATTCCCCGACGGGGAG	49	ND	ND	ND	ND
		tRF-49-KS3W2VR008R9D9KUMKEV	CCCCGCCTGTCACGCGGGAGACCGGGGTTCAATTCCCCGACGGGGAGCC	49	0,670558393	25,5493353	28,4080133	24,5964426

		tRF-49-KVNMEH623K76IR3DR2I2	CCCGCCTGTCACGCGGGAGACCGGGGTTCAATTCCCCGACGGGGAGCCA	49	0,075443596	122,601382	160,175877	110,07655
		tRF-50-8RPY8HM2OSRN2NKSEK51	TCCCCGCCTGTCACGCGGGAGACCGGGGTTCAATTCCCCGACGGGGAGCC	50	0,127584127	9,45997139	15,1316147	7,56942361
		tRF-50-HRL7S5QKF1R38E8RO86J	ATCCCCGCCTGTCACGCGGGAGACCGGGGTTCAATTCCCCGACGGGGAGC	50	0,927135368	7,21918707	7,96599334	6,97025165
		tRF-50-KS3W2VR008R9D9KUMKF6	CCCCGCCTGTCACGCGGGAGACCGGGGTTCAATTCCCCGACGGGGAGCCA	50	0,075275944	174,753841	223,979355	158,345336
		tRF-50-VR3196L85DMKYOYRLHR0	TATCCCCGCCTGTCACGCGGGAGACCGGGGTTCAATTCCCCGACGGGGAG	50	ND	ND	ND	ND

tRNA	Variant	tsRNA ID (MINTbase)	Sequence	Seq length	PCa_vs_BPH_pvalue	baseMean	BPH mean	PCa mean
Asp-GTC	3'-half	tRF-40-8HM2OSRN2NKSEK51	TCACGCGGGAGACCGGGGTTCAATTCCCCGACGGGGAGCC	40	0,003353302	1470,10996	2319,01199	1187,14262
		tRF-41-8HM2OSRN2NKSEK51B	TCACGCGGGAGACCGGGGTTCAATTCCCCGACGGGGAGCCA	41	0,00099071	7786,16879	12374,0054	6256,88993
		tRF-41-S5QKF1R38E8RO86JD	GTCACGCGGGAGACCGGGGTTCAATTCCCCGACGGGGAGCC	41	0,12491749	100,2161	136,818144	88,0154182
		tRF-42-96L85DMKYOYRLHR0J	TGTCACGCGGGAGACCGGGGTTCAATTCCCCGACGGGGAGCC	42	0,108612668	87,8951927	118,344823	77,745316
		tRF-42-S5QKF1R38E8RO86J2	GTCACGCGGGAGACCGGGGTTCAATTCCCCGACGGGGAGCCA	42	0,233029438	416,055461	511,28537	384,312159
		tRF-43-96L85DMKYOYRLHR0D2	TGTCACGCGGGAGACCGGGGTTCAATTCCCCGACGGGGAGCCA	43	0,06808648	374,207029	484,572322	337,418598
		tRF-43-NMEH623K76IR3DR2DV	CTGTCACGCGGGAGACCGGGGTTCAATTCCCCGACGGGGAGCC	43	0,0627141	139,333546	191,18656	122,049208
		tRF-44-3W2VR008R9D9KUMKEV	CCTGTCACGCGGGAGACCGGGGTTCAATTCCCCGACGGGGAGCC	44	0,2081577	57,0944839	75,1522411	51,0752316
		tRF-44-NMEH623K76IR3DR2I2	CTGTCACGCGGGAGACCGGGGTTCAATTCCCCGACGGGGAGCCA	44	0,042968156	949,708407	1278,75129	840,027445
		tRF-45-3W2VR008R9D9KUMKF6	CCTGTCACGCGGGAGACCGGGGTTCAATTCCCCGACGGGGAGCCA	45	0,034470265	829,353411	1172,68301	714,910211
		tRF-45-PY8HM2OSRN2NKSEK51	GCCTGTCACGCGGGAGACCGGGGTTCAATTCCCCGACGGGGAGCC	45	0,198641346	184,467772	241,051398	165,606563
		tRF-46-L7S5QKF1R38E8RO86JD	CGCCTGTCACGCGGGAGACCGGGGTTCAATTCCCCGACGGGGAGCC	46	0,218786371	27,2039127	36,2126396	24,2010037
		tRF-46-PY8HM2OSRN2NKSEK51B	GCCTGTCACGCGGGAGACCGGGGTTCAATTCCCCGACGGGGAGCCA	46	0,083981876	954,905858	1254,3434	855,093342
		tRF-47-3196L85DMKYOYRLHR0J	CCGCCTGTCACGCGGGAGACCGGGGTTCAATTCCCCGACGGGGAGCC	47	0,073455211	42,9157207	60,6486904	37,0047309
		tRF-47-L7S5QKF1R38E8RO86J2	CGCCTGTCACGCGGGAGACCGGGGTTCAATTCCCCGACGGGGAGCCA	47	0,116895052	634,817229	818,023082	573,748611
		tRF-48-3196L85DMKYOYRLHR0D2	CCGCCTGTCACGCGGGAGACCGGGGTTCAATTCCCCGACGGGGAGCCA	48	0,038245798	535,934567	770,442752	457,765171
		tRF-48-KVNMEH623K76IR3DR2DV	CCCGCCTGTCACGCGGGAGACCGGGGTTCAATTCCCCGACGGGGAGCC	48	0,133414842	9,01748589	13,7012395	7,4562347
		tRF-49-KS3W2VR008R9D9KUMKEV	CCCCGCCTGTCACGCGGGAGACCGGGGTTCAATTCCCCGACGGGGAGCC	49	0,670558393	25,5493353	28,4080133	24,5964426
		tRF-49-KVNMEH623K76IR3DR2I2	CCCGCCTGTCACGCGGGAGACCGGGGTTCAATTCCCCGACGGGGAGCCA	49	0,075443596	122,601382	160,175877	110,07655
		tRF-50-8RPY8HM2OSRN2NKSEK51	TCCCCGCCTGTCACGCGGGAGACCGGGGTTCAATTCCCCGACGGGGAGCC	50	0,127584127	9,45997139	15,1316147	7,56942361
		tRF-50-KS3W2VR008R9D9KUMKF6	CCCCGCCTGTCACGCGGGAGACCGGGGTTCAATTCCCCGACGGGGAGCCA	50	0,075275944	174,753841	223,979355	158,345336

tRNA	Variant	tsRNA ID (MINTbase)	Sequence	Seq length	PCa_vs_BPH_pvalue	baseMean	BPH mean	PCa mean
Asp-GTC	3'-half	tRF-41-8HM2OSRN2NKSEK51B	TCACGCGGGAGACCGGGGTTCAATTCCCCGACGGGGAGCCA	41	0,00099071	7786,16879	12374,0054	6256,88993
		tRF-45-3W2VR008R9D9KUMKF6	CCTGTCACGCGGGAGACCGGGGTTCAATTCCCCGACGGGGAGCCA	45	0,034470265	829,353411	1172,68301	714,910211
		tRF-46-PY8HM2OSRN2NKSEK51B	GCCTGTCACGCGGGAGACCGGGGTTCAATTCCCCGACGGGGAGCCA	46	0,083981876	954,905858	1254,3434	855,093342
		tRF-44-NMEH623K76IR3DR2I2	CTGTCACGCGGGAGACCGGGGTTCAATTCCCCGACGGGGAGCCA	44	0,042968156	949,708407	1278,75129	840,027445
		tRF-47-L7S5QKF1R38E8RO86J2	CGCCTGTCACGCGGGAGACCGGGGTTCAATTCCCCGACGGGGAGCCA	47	0,116895052	634,817229	818,023082	573,748611
		tRF-48-3196L85DMKYOYRLHR0D2	CCGCCTGTCACGCGGGAGACCGGGGTTCAATTCCCCGACGGGGAGCCA	48	0,038245798	535,934567	770,442752	457,765171
		tRF-43-96L85DMKYOYRLHR0D2	TGTCACGCGGGAGACCGGGGTTCAATTCCCCGACGGGGAGCCA	43	0,06808648	374,207029	484,572322	337,418598
		tRF-42-S5QKF1R38E8RO86J2	GTCACGCGGGAGACCGGGGTTCAATTCCCCGACGGGGAGCCA	42	0,233029438	416,055461	511,28537	384,312159
		tRF-50-KS3W2VR008R9D9KUMKF6	CCCCGCCTGTCACGCGGGAGACCGGGGTTCAATTCCCCGACGGGGAGCCA	50	0,075275944	174,753841	223,979355	158,345336
		tRF-49-KVNMEH623K76IR3DR2I2	CCCGCCTGTCACGCGGGAGACCGGGGTTCAATTCCCCGACGGGGAGCCA	49	0,075443596	122,601382	160,175877	110,07655

tRNA	Variant	tsRNA ID (MINTbase)	Sequence	Seq length	PCa_vs_BPH_pvalue	baseMean	BPH mean	PCa mean
Thr-TGT	3'-tRF	tRF-17-D7LYR15	AATCTCGCTGGGGCCTC	17	0,004220627	137,929953	83,4608814	156,08631
		tRF-18-BMWIRJD5	AAATCTCGCTGGGGCCTC	18	0,097388429	107,640878	77,6141967	117,649772
		tRF-18-D7LYR10J	AATCTCGCTGGGGCCTCC	18	0,734396957	184,092039	176,546891	186,607088
		tRF-19-2E4V9K15	CAAATCTCGCTGGGGCCTC	19	0,187892582	80,8727369	55,5506267	89,3134402
		tRF-19-BMWIRJHJ	AAATCTCGCTGGGGCCTCC	19	0,674672349	138,087718	121,642374	143,569499
		tRF-19-D7LYR1J2	AATCTCGCTGGGGCCTCCA	19	0,367856668	695,727319	628,535469	718,124602

tRF-20-2E4V9K3R	CAAATCTCGCTGGGGCCTCC	20	0,732951309	66,3514538	60,5763406	68,2764916
tRF-20-8BWSNKP9	TCAAATCTCGCTGGGGCCTC	20	0,319028126	113,007836	99,2562092	117,591711
tRF-20-BMWIRJ46	AAATCTCGCTGGGGCCTCCA	20	0,392487645	550,888574	481,167964	574,128777
tRF-21-2E4V9K3RB	CAAATCTCGCTGGGGCCTCCA	21	0,47270674	341,681967	304,535756	354,064037
tRF-21-8BWSNKP9D	TCAAATCTCGCTGGGGCCTCC	21	0,719223302	99,4958891	107,77308	96,7368254
tRF-21-YOH9Q867D	TTCAAATCTCGCTGGGGCCTC	21	0,291351723	71,957566	60,0897205	75,9135144
tRF-22-76D7LYR15	GTTCAAATCTCGCTGGGGCCTC	22	0,142513353	107,80743	70,8149294	120,138263
tRF-22-8BWSNKP92	TCAAATCTCGCTGGGGCCTCCA	22	0,545906852	533,811228	513,469423	540,591829
tRF-22-YOH9Q867J	TTCAAATCTCGCTGGGGCCTCC	22	0,803859533	73,0875526	73,78681	72,8544668
tRF-23-19BMWIRJD5	AGTTCAAATCTCGCTGGGGCCTC	23	0,013710113	171,449084	106,738533	193,019267
tRF-23-76D7LYR10J	GTTCAAATCTCGCTGGGGCCTCC	23	0,277362445	103,605271	87,3247463	109,032112
tRF-23-YOH9Q867D2	TTCAAATCTCGCTGGGGCCTCCA	23	0,546004511	291,857602	264,126728	301,101227
tRF-24-19BMWIRJHJ	AGTTCAAATCTCGCTGGGGCCTCC	24	0,028473867	97,2641582	67,3006367	107,251999
tRF-24-5N2E4V9K15	GAGTTCAAATCTCGCTGGGGCCTC	24	0,054743225	102,894017	65,4431226	115,377648
tRF-24-76D7LYR1J2	GTTCAAATCTCGCTGGGGCCTCCA	24	0,361092648	211,565539	187,759416	219,500913
tRF-25-19BMWIRJ46	AGTTCAAATCTCGCTGGGGCCTCCA	25	0,386023641	495,202546	419,667997	520,380729
tRF-25-5N2E4V9K3R	GAGTTCAAATCTCGCTGGGGCCTCC	25	0,65596541	65,0119998	57,1937209	67,6180928
tRF-25-L38BWSNKP9	CGAGTTCAAATCTCGCTGGGGCCTC	25	0,066210429	132,177559	89,4431207	146,422371
tRF-26-5N2E4V9K3RB	GAGTTCAAATCTCGCTGGGGCCTCCA	26	0,396462116	162,285576	132,085714	172,352197
tRF-26-L38BWSNKP9D	CGAGTTCAAATCTCGCTGGGGCCTCC	26	0,729281929	95,4688573	86,0373504	98,6126929
tRF-26-Q0YOH9Q867D	GCGAGTTCAAATCTCGCTGGGGCCTC	26	0,917305113	6,91394001	6,49914796	7,05220402
tRF-27-L38BWSNKP92	CGAGTTCAAATCTCGCTGGGGCCTCCA	27	0,806373113	250,425965	228,709972	257,664629
tRF-27-LU76D7LYR15	CGCGAGTTCAAATCTCGCTGGGGCCTC	27	0,063762568	6086,13593	8425,40457	5306,37972
tRF-27-Q0YOH9Q867J	GCGAGTTCAAATCTCGCTGGGGCCTCC	27	ND	ND	ND	ND
tRF-28-LU76D7LYR10J	CGCGAGTTCAAATCTCGCTGGGGCCTCC	28	0,014864381	1874,10372	2996,48061	1499,97809
tRF-28-Q0YOH9Q867D2	GCGAGTTCAAATCTCGCTGGGGCCTCCA	28	ND	ND	ND	ND
tRF-28-WH19BMWIRJD5	TCGCGAGTTCAAATCTCGCTGGGGCCTC	28	0,231201379	3502,44871	4498,95286	3170,28065
tRF-29-LU76D7LYR1J2	CGCGAGTTCAAATCTCGCTGGGGCCTCCA	29	0,101208686	3807,78992	5371,00467	3286,71833
tRF-29-SV5N2E4V9K15	GTCGCGAGTTCAAATCTCGCTGGGGCCTC	29	0,812623272	108,408189	108,540802	108,363984
tRF-29-WH19BMWIRJHJ	TCGCGAGTTCAAATCTCGCTGGGGCCTCC	29	0,075153457	1818,80417	2685,74908	1529,82253
tRF-30-RSL38BWSNKP9	GGTCGCGAGTTCAAATCTCGCTGGGGCCTC	30	0,414441145	76,1590621	63,3343575	80,4339637
tRF-30-SV5N2E4V9K3R	GTCGCGAGTTCAAATCTCGCTGGGGCCTCC	30	0,505824152	51,1861729	62,8893666	47,2851083
tRF-30-WH19BMWIRJ46	TCGCGAGTTCAAATCTCGCTGGGGCCTCCA	30	0,242784807	2128,84651	2797,85969	1905,84211
tRF-31-RMQ0YOH9Q867D	GGGTCGCGAGTTCAAATCTCGCTGGGGCCTC	31	0,50129508	16,9151632	12,1060138	18,518213
tRF-31-RSL38BWSNKP9D	GGTCGCGAGTTCAAATCTCGCTGGGGCCTCC	31	0,739429026	46,1292534	41,4110863	47,7019758
tRF-31-SV5N2E4V9K3RB	GTCGCGAGTTCAAATCTCGCTGGGGCCTCCA	31	0,395864238	107,944361	125,270625	102,168939
tRF-32-R3LU76D7LYR15	GGGGTCGCGAGTTCAAATCTCGCTGGGGCCTC	32	ND	ND	ND	ND
tRF-32-RMQ0YOH9Q867J	GGGTCGCGAGTTCAAATCTCGCTGGGGCCTCC	32	0,724945477	17,3409545	14,8116671	18,1840504
tRF-32-RSL38BWSNKP92	GGTCGCGAGTTCAAATCTCGCTGGGGCCTCCA	32	0,690109288	103,51999	108,056065	102,007965
tRF-33-1KWH19BMWIRJD5	AGGGGTCGCGAGTTCAAATCTCGCTGGGGCCTC	33	ND	ND	ND	ND
tRF-33-R3LU76D7LYR10J	GGGGTCGCGAGTTCAAATCTCGCTGGGGCCTCC	33	ND	ND	ND	ND
tRF-33-RMQ0YOH9Q867D2	GGGTCGCGAGTTCAAATCTCGCTGGGGCCTCCA	33	0,179368845	27,2856245	15,7572659	31,1284107
tRF-34-13LU76D7LYR1J2	AGGGTCGCGAGTTCAAATCTCGCTGGGGCCTCCA	34	ND	ND	ND	ND
tRF-34-1KWH19BMWIRJHJ	AGGGGTCGCGAGTTCAAATCTCGCTGGGGCCTCC	34	ND	ND	ND	ND
tRF-34-JKSV5N2E4V9K15	CAGGGGTCGCGAGTTCAAATCTCGCTGGGGCCTC	34	0,616619615	11,0554868	13,1099389	10,3706694

tRF-34-KKSV5N2E4V9K15	CCAGGGTCGCGAGTTCAAATCTCGCTGGGGCCTC	34	ND	ND	ND	ND
tRF-34-R3LU76D7LYR1J2	GGGGTCGCGAGTTCAAATCTCGCTGGGGCCTCCA	34	ND	ND	ND	ND
tRF-35-1KWH19BMWIRJ46	AGGGGTCGCGAGTTCAAATCTCGCTGGGGCCTCCA	35	ND	ND	ND	ND
tRF-35-JKSV5N2E4V9K3R	CAGGGGTCGCGAGTTCAAATCTCGCTGGGGCCTCC	35	0,587420772	13,1295189	11,0444199	13,8245519
tRF-35-KKRSL38BWSNKP9	CCAGGGGTCGCGAGTTCAAATCTCGCTGGGGCCTC	35	0,973942907	10,9267369	11,8471489	10,6199329
tRF-36-0PRMQ0YOH9Q867D	ACCAGGGGTCGCGAGTTCAAATCTCGCTGGGGCCTC	36	0,161446215	10,6981287	7,09823214	11,8980942
tRF-36-0PRSL38BWSNKP9D	ACCAGGGTCGCGAGTTCAAATCTCGCTGGGGCCTCC	36	ND	ND	ND	ND
tRF-36-B6RMQ0YOH9Q867D	AACCAGGGTCGCGAGTTCAAATCTCGCTGGGGCCTC	36	ND	ND	ND	ND
tRF-36-JKSV5N2E4V9K3RB	CAGGGGTCGCGAGTTCAAATCTCGCTGGGGCCTCCA	36	0,688862822	21,2571683	20,0529821	21,6585637
tRF-36-KKRSL38BWSNKP9D	CCAGGGGTCGCGAGTTCAAATCTCGCTGGGGCCTCC	36	0,676000203	15,7274615	13,5645256	16,4484401
tRF-37-0PRMQ0YOH9Q867J	ACCAGGGGTCGCGAGTTCAAATCTCGCTGGGGCCTCC	37	0,497490609	20,502518	16,622431	21,7958804
tRF-37-B6R3LU76D7LYR15	AACCAGGGGTCGCGAGTTCAAATCTCGCTGGGGCCTC	37	0,897822798	32,5431895	33,8933667	32,0931304
tRF-37-B6RMQ0YOH9Q867J	AACCAGGGTCGCGAGTTCAAATCTCGCTGGGGCCTCC	37	ND	ND	ND	ND
tRF-37-KKRSL38BWSNKP92	CCAGGGGTCGCGAGTTCAAATCTCGCTGGGGCCTCCA	37	0,305266652	24,945448	18,8307616	26,9836768
tRF-38-0PRMQ0YOH9Q867D2	ACCAGGGGTCGCGAGTTCAAATCTCGCTGGGGCCTCCA	38	0,338428538	23,5405182	16,9606598	25,7338043
tRF-38-B11KWH19BMWIRJD5	AAACCAGGGGTCGCGAGTTCAAATCTCGCTGGGGCCTC	38	0,348838911	15,2344649	12,3061171	16,2105808
tRF-38-B6R3LU76D7LYR10J	AACCAGGGGTCGCGAGTTCAAATCTCGCTGGGGCCTCC	38	0,066987447	33,3599362	53,0744593	26,7884284
tRF-38-B6RMQ0YOH9Q867D2	AACCAGGGTCGCGAGTTCAAATCTCGCTGGGGCCTCCA	38	ND	ND	ND	ND
tRF-39-B113LU76D7LYR1J2	AAACCAGGGTCGCGAGTTCAAATCTCGCTGGGGCCTCCA	39	ND	ND	ND	ND
tRF-39-B11KWH19BMWIRJHJ	AAACCAGGGGTCGCGAGTTCAAATCTCGCTGGGGCCTCC	39	0,899945271	10,8497302	10,0686313	11,1100965
tRF-39-B6R3LU76D7LYR1J2	AACCAGGGGTCGCGAGTTCAAATCTCGCTGGGGCCTCCA	39	0,549002616	60,0209785	71,0679791	56,338645
tRF-39-UDJKSV5N2E4V9K15	TAAACCAGGGGTCGCGAGTTCAAATCTCGCTGGGGCCTC	39	0,666831145	15,4853901	17,1187381	14,9409407
tRF-39-UDJKWH19BMWIRJHJ	TAAACCAGGGTCGCGAGTTCAAATCTCGCTGGGGCCTCC	39	ND	ND	ND	ND
tRF-40-B11KWH19BMWIRJ46	AAACCAGGGGTCGCGAGTTCAAATCTCGCTGGGGCCTCCA	40	0,06240251	17,37327	9,42296835	20,0233705
tRF-40-SBKKRSL38BWSNKP9	GTAAACCAGGGGTCGCGAGTTCAAATCTCGCTGGGGCCTC	40	0,676954487	17,8135927	16,3240455	18,3101084
tRF-40-UDJKSV5N2E4V9K3R	TAAACCAGGGGTCGCGAGTTCAAATCTCGCTGGGGCCTCC	40	0,786530862	9,26691758	7,81019156	9,75249293
tRF-40-UDJKWH19BMWIRJ46	TAAACCAGGGTCGCGAGTTCAAATCTCGCTGGGGCCTCCA	40	ND	ND	ND	ND
tRF-41-9O0PRMQ0YOH9Q867D	TGTAAACCAGGGGTCGCGAGTTCAAATCTCGCTGGGGCCTC	41	ND	ND	ND	ND
tRF-41-9O0PRSL38BWSNKP9D	TGTAAACCAGGGTCGCGAGTTCAAATCTCGCTGGGGCCTCC	41	ND	ND	ND	ND
tRF-41-SBKKRSL38BWSNKP9D	GTAAACCAGGGGTCGCGAGTTCAAATCTCGCTGGGGCCTCC	41	0,201606505	14,6513432	9,88097206	16,2414669
tRF-41-SBKKSV5N2E4V9K3RB	GTAAACCAGGGTCGCGAGTTCAAATCTCGCTGGGGCCTCCA	41	ND	ND	ND	ND
tRF-41-UDJKSV5N2E4V9K3RB	TAAACCAGGGGTCGCGAGTTCAAATCTCGCTGGGGCCTCCA	41	0,59247387	14,5331022	16,1907926	13,9805388
tRF-41-ZLB6RMQ0YOH9Q867D	TTGTAAACCAGGGTCGCGAGTTCAAATCTCGCTGGGGCCTC	41	ND	ND	ND	ND
tRF-42-9O0PRMQ0YOH9Q867J	TGTAAACCAGGGGTCGCGAGTTCAAATCTCGCTGGGGCCTCC	42	ND	ND	ND	ND
tRF-42-SBKKRSL38BWSNKP92	GTAAACCAGGGGTCGCGAGTTCAAATCTCGCTGGGGCCTCCA	42	0,587913832	19,2781349	15,6207131	20,4972756
tRF-42-ZLB6R3LU76D7LYR15	TTGTAAACCAGGGGTCGCGAGTTCAAATCTCGCTGGGGCCTC	42	0,731178049	155,866325	193,82799	143,212437
tRF-42-ZLB6RMQ0YOH9Q867J	TTGTAAACCAGGGTCGCGAGTTCAAATCTCGCTGGGGCCTCC	42	ND	ND	ND	ND
tRF-43-9O0PRMQ0YOH9Q867D2	TGTAAACCAGGGGTCGCGAGTTCAAATCTCGCTGGGGCCTCCA	43	0,680469038	6,26499549	4,70376075	6,78540707
tRF-43-NWB11KWH19BMWIRJD5	CTTGTAAACCAGGGGTCGCGAGTTCAAATCTCGCTGGGGCCTC	43	ND	ND	ND	ND
tRF-43-ZLB6R3LU76D7LYR10J	TTGTAAACCAGGGGTCGCGAGTTCAAATCTCGCTGGGGCCTCC	43	0,319952982	124,194208	186,345336	103,477166
tRF-43-ZLB6RMQ0YOH9Q867D2	TTGTAAACCAGGGTCGCGAGTTCAAATCTCGCTGGGGCCTCCA	43	ND	ND	ND	ND
tRF-44-NWB11KWH19BMWIRJHJ	CTTGTAAACCAGGGGTCGCGAGTTCAAATCTCGCTGGGGCCTCC	44	ND	ND	ND	ND
tRF-44-WYUDJKSV5N2E4V9K15	TCTTGTAAACCAGGGGTCGCGAGTTCAAATCTCGCTGGGGCCTC	44	ND	ND	ND	ND
tRF-44-ZLB6R3LU76D7LYR1J2	TTGTAAACCAGGGGTCGCGAGTTCAAATCTCGCTGGGGCCTCCA	44	0,48052987	193,601889	262,860305	170,51575
tRF-45-NWB11KWH19BMWIRJ46	CTTGTAAACCAGGGGTCGCGAGTTCAAATCTCGCTGGGGCCTCCA	45	ND	ND	ND	ND

		tRF-45-SZSBKKRSL38BWSNKP9	GTCTTGTAAACCAGGGGTCGCGAGTTCAAATCTCGCTGGGGCCTC	45	ND	ND	ND	ND
		tRF-45-WYUDJKSV5N2E4V9K3R	TCTTGTAAACCAGGGGTCGCGAGTTCAAATCTCGCTGGGGCCTCC	45	ND	ND	ND	ND
		tRF-46-SZSBKKRSL38BWSNKP9D	GTCTTGTAAACCAGGGGTCGCGAGTTCAAATCTCGCTGGGGCCTCC	46	ND	ND	ND	ND
		tRF-46-WYUDJKSV5N2E4V9K3RB	TCTTGTAAACCAGGGGTCGCGAGTTCAAATCTCGCTGGGGCCTCCA	46	ND	ND	ND	ND
		tRF-47-SZSBKKRSL38BWSNKP92	GTCTTGTAAACCAGGGGTCGCGAGTTCAAATCTCGCTGGGGCCTCCA	47	ND	ND	ND	ND
		tRF-48-9MZLB6R3LU76D7LYR10J	TGGTCTTGTAAACCAGGGGTCGCGAGTTCAAATCTCGCTGGGGCCTCC	48	ND	ND	ND	ND
		tRF-49-9MZLB6R3LU76D7LYR1J2	TGGTCTTGTAAACCAGGGGTCGCGAGTTCAAATCTCGCTGGGGCCTCCA	49	ND	ND	ND	ND
		tRF-49-E8WYUDJKSV5N2E4V9K15	ACTGGTCTTGTAAACCAGGGGTCGCGAGTTCAAATCTCGCTGGGGCCTC	49	ND	ND	ND	ND
		tRF-50-2YSZSBKKRSL38BWSNKP9	CACTGGTCTTGTAAACCAGGGGTCGCGAGTTCAAATCTCGCTGGGGCCTC	50	ND	ND	ND	ND
		tRF-50-E8WYUDJKSV5N2E4V9K3R	ACTGGTCTTGTAAACCAGGGGTCGCGAGTTCAAATCTCGCTGGGGCCTCC	50	ND	ND	ND	ND
		tRF-50-N3NWB11KWH19BMWIRJ46	CTGGTCTTGTAAACCAGGGGTCGCGAGTTCAAATCTCGCTGGGGCCTCCA	50	ND	ND	ND	ND

tRNA	Variant	tsRNA ID (MINTbase)	Sequence	Seq length	PCa_vs_BPH_pvalue	baseMean	BPH mean	PCa mean
Val-CAC	3'-tRF	tRF-23-7SB1RHODV	GTTCGAAACCGGGCGGAAACACC	23	0,000792127	203,96134	99,4629147	238,794148
		tRF-24-7SB1RHODE2	GTTCGAAACCGGGCGGAAACACCA	24	0,050422045	2727,79986	2075,70218	2945,16576
		tRF-24-R9ODMJ6BFV	GGTTCGAAACCGGGCGGAAACACC	24	0,262558657	121,882224	91,8497458	131,89305
		tRF-25-MNLB3KMB01	CGGTTCGAAACCGGGCGGAAACACC	25	0,020927429	31,7581458	10,5827095	38,8166245
		tRF-25-R9ODMJ6B26	GGTTCGAAACCGGGCGGAAACACCA	25	0,001810681	462,268389	264,720287	528,117756
		tRF-26-33WB08Q2B5D	CCGGTTCGAAACCGGGCGGAAACACC	26	0,80569456	55,4326209	52,3595483	56,4569785
		tRF-26-MNLB3KMB01B	CGGTTCGAAACCGGGCGGAAACACCA	26	0,123387945	717,326589	544,63784	774,889505
		tRF-27-33WB08Q2B52	CCGGTTCGAAACCGGGCGGAAACACCA	27	0,415211519	307,534062	272,946657	319,063197
		tRF-27-K8YUBS68BFJ	CCCGGTTCGAAACCGGGCGGAAACACC	27	0,033528571	9,81900045	2,96370759	12,1040981
		tRF-27-Q3WB08Q2B52	GCGGTTCGAAACCGGGCGGAAACACCA	27	ND	ND	ND	ND
		tRF-28-K8YUBS68BFD2	CCCGGTTCGAAACCGGGCGGAAACACCA	28	0,474703334	8,86471654	12,4326418	7,67540811
		tRF-28-KS7SB1RHODV	CCCCGGTTCGAAACCGGGCGGAAACACC	28	0,234754495	3554,61859	4260,80943	3319,22165
		tRF-29-3H7SB1RHODE2	CCGCGGTTCGAAACCGGGCGGAAACACCA	29	ND	ND	ND	ND
		tRF-29-8RR9ODMJ6BFV	TCCCCGGTTCGAAACCGGGCGGAAACACC	29	0,510647867	5092,78127	5805,73324	4855,13061
		tRF-29-KS7SB1RHODE2	CCCCGGTTCGAAACCGGGCGGAAACACCA	29	0,394735405	11344,5536	13233,4384	10714,9253
		tRF-30-8RR9ODMJ6B26	TCCCCGGTTCGAAACCGGGCGGAAACACCA	30	0,706747778	8706,10914	9736,06966	8362,78897
		tRF-30-8VR9ODMJ6B26	TCCGCGGTTCGAAACCGGGCGGAAACACCA	30	ND	ND	ND	ND
		tRF-30-SRMNLB3KMB01	GTCCCCGGTTCGAAACCGGGCGGAAACACC	30	0,021319938	26,2491255	14,8643134	30,0440629
		tRF-30-SSMNLB3KMB01	GTCCGCGGTTCGAAACCGGGCGGAAACACC	30	ND	ND	ND	ND
		tRF-31-RR33WB08Q2B5D	GGTCCCCGGTTCGAAACCGGGCGGAAACACC	31	0,856067002	48,9664781	48,7407081	49,0417347
		tRF-31-SRMNLB3KMB01B	GTCCCCGGTTCGAAACCGGGCGGAAACACCA	31	0,057070135	162,064796	111,749629	178,836518
		tRF-32-1MK8YUBS68BFJ	AGGTCCCCGGTTCGAAACCGGGCGGAAACACC	32	0,474141786	68,7132361	59,6257075	71,7424123
		tRF-32-RR33WB08Q2B52	GGTCCCCGGTTCGAAACCGGGCGGAAACACCA	32	0,089993426	204,942603	151,792153	222,65942
		tRF-33-1MK8YUBS68BFD2	AGGTCCCCGGTTCGAAACCGGGCGGAAACACCA	33	0,305847305	199,734594	166,898205	210,680057
		tRF-33-D3KS7SB1RHODV	AAGGTCCCCGGTTCGAAACCGGGCGGAAACACC	33	0,104443581	121,587242	87,9838872	132,78836
		tRF-34-BK8RR9ODMJ6BFV	AAAGGTCCCCGGTTCGAAACCGGGCGGAAACACC	34	0,038307017	158,559448	107,609697	175,542698
		tRF-34-D3KS7SB1RHODE2	AAGGTCCCCGGTTCGAAACCGGGCGGAAACACCA	34	0,000120658	222,247416	127,819628	253,723346
		tRF-35-BK8RR9ODMJ6B26	AAAGGTCCCCGGTTCGAAACCGGGCGGAAACACCA	35	0,163480374	303,887677	237,594915	325,985264
		tRF-35-O0SRMNLB3KMB01	GAAAGGTCCCCGGTTCGAAACCGGGCGGAAACACC	35	0,130199676	88,0266097	60,9374222	97,0563389
		tRF-35-U0SRMNLB3KMB01	TAAAGGTCCCCGGTTCGAAACCGGGCGGAAACACC	35	ND	ND	ND	ND
		tRF-36-LBRR33WB08Q2B5D	CGAAAGGTCCCCGGTTCGAAACCGGGCGGAAACACC	36	0,065826719	130,364833	92,0480128	143,137106
		tRF-36-O0SRMNLB3KMB01B	GAAAGGTCCCCGGTTCGAAACCGGGCGGAAACACCA	36	0,009413158	129,020567	74,4947248	147,195847
		tRF-36-U0SRMNLB3KMB01B	TAAAGGTCCCCGGTTCGAAACCGGGCGGAAACACCA	36	ND	ND	ND	ND

		tRF-37-LBRR33WB08Q2B52	CGAAAGGTCCCCGGTTCGAAACCGGGCGGAAACACCA	37	0,100939667	183,248764	123,91482	203,026746
		tRF-37-QB1MK8YUBS68BFJ	GCGAAAGGTCCCCGGTTCGAAACCGGGCGGAAACACC	37	0,59809267	114,809018	104,14632	118,363251
		tRF-38-LUD3KS7SB1RHODV	CGCGAAAGGTCCCCGGTTCGAAACCGGGCGGAAACACC	38	0,505724322	118,28158	98,4065747	124,906582
		tRF-38-QB1MK8YUBS68BFD2	GCGAAAGGTCCCCGGTTCGAAACCGGGCGGAAACACCA	38	0,847495823	247,465431	265,009148	241,617525
		tRF-39-EHBK8RR9ODMJ6BFV	ACGCGAAAGGTCCCCGGTTCGAAACCGGGCGGAAACACC	39	0,546779152	143,085175	121,122528	150,406057
		tRF-39-LUD3KS7SB1RHODE2	CGCGAAAGGTCCCCGGTTCGAAACCGGGCGGAAACACCA	39	0,838395385	210,846039	196,24112	215,714346
		tRF-40-2VO0SRMNLB3KMB01	CACGCGAAAGGTCCCCGGTTCGAAACCGGGCGGAAACACC	40	0,145974283	125,834015	106,677652	132,219469
		tRF-40-EHBK8RR9ODMJ6B26	ACGCGAAAGGTCCCCGGTTCGAAACCGGGCGGAAACACCA	40	0,960820847	242,938901	231,610603	246,715001
		tRF-41-0HLBRR33WB08Q2B5D	ACACGCGAAAGGTCCCCGGTTCGAAACCGGGCGGAAACACC	41	0,14941681	174,325423	127,271822	190,009956
		tRF-41-2VO0SRMNLB3KMB01B	CACGCGAAAGGTCCCCGGTTCGAAACCGGGCGGAAACACCA	41	0,238968201	297,164547	244,238681	314,806503
		tRF-42-0HLBRR33WB08Q2B52	ACACGCGAAAGGTCCCCGGTTCGAAACCGGGCGGAAACACCA	42	0,863272442	388,94059	347,42373	402,779544
		tRF-42-25QB1MK8YUBS68BFJ	CACACGCGAAAGGTCCCCGGTTCGAAACCGGGCGGAAACACC	42	0,352794568	51,4388468	42,4143919	54,4469984
		tRF-42-B5QB1MK8YUBS68BFJ	AACACGCGAAAGGTCCCCGGTTCGAAACCGGGCGGAAACACC	42	ND	ND	ND	ND
		tRF-43-25QB1MK8YUBS68BFD2	CACACGCGAAAGGTCCCCGGTTCGAAACCGGGCGGAAACACCA	43	0,615187128	101,661375	107,922912	99,5741962
		tRF-43-8FLUD3KS7SB1RHODV	TCACACGCGAAAGGTCCCCGGTTCGAAACCGGGCGGAAACACC	43	0,110726905	29,2460278	17,8977345	33,0287923
		tRF-43-B5QB1MK8YUBS68BFD2	AACACGCGAAAGGTCCCCGGTTCGAAACCGGGCGGAAACACCA	43	ND	ND	ND	ND
		tRF-44-45EHBK8RR9ODMJ6BFV	CTCACACGCGAAAGGTCCCCGGTTCGAAACCGGGCGGAAACACC	44	0,825111638	41,0174736	32,5050968	43,8549326
		tRF-44-8FLUD3KS7SB1RHODE2	TCACACGCGAAAGGTCCCCGGTTCGAAACCGGGCGGAAACACCA	44	0,680961177	63,7555257	66,857541	62,7215206
		tRF-45-362VO0SRMNLB3KMB01	CCTCACACGCGAAAGGTCCCCGGTTCGAAACCGGGCGGAAACACC	45	0,918646037	126,583378	131,695034	124,879493
		tRF-45-45EHBK8RR9ODMJ6B26	CTCACACGCGAAAGGTCCCCGGTTCGAAACCGGGCGGAAACACCA	45	0,671962835	100,614012	92,0922037	103,454614
		tRF-45-4DEHBK8RR9ODMJ6B26	CTAACACGCGAAAGGTCCCCGGTTCGAAACCGGGCGGAAACACCA	45	ND	ND	ND	ND
		tRF-46-362VO0SRMNLB3KMB01B	CCTCACACGCGAAAGGTCCCCGGTTCGAAACCGGGCGGAAACACCA	46	0,164870231	299,011014	372,487803	274,518751
		tRF-46-3O2VO0SRMNLB3KMB01B	CCTAACACGCGAAAGGTCCCCGGTTCGAAACCGGGCGGAAACACCA	46	ND	ND	ND	ND
		tRF-46-P90HLBRR33WB08Q2B5D	GCCTCACACGCGAAAGGTCCCCGGTTCGAAACCGGGCGGAAACACC	46	0,86743601	53,0035715	53,2067623	52,9358412
		tRF-47-L725QB1MK8YUBS68BFJ	CGCCTCACACGCGAAAGGTCCCCGGTTCGAAACCGGGCGGAAACACC	47	0,300617658	41,3028374	51,4538217	37,9191759
		tRF-47-P90HLBRR33WB08Q2B52	GCCTCACACGCGAAAGGTCCCCGGTTCGAAACCGGGCGGAAACACCA	47	0,900840754	166,787234	169,963502	165,728478
		tRF-48-L725QB1MK8YUBS68BFD2	CGCCTCACACGCGAAAGGTCCCCGGTTCGAAACCGGGCGGAAACACCA	48	0,982621627	139,537164	139,08775	139,686968
		tRF-48-W18FLUD3KS7SB1RHODV	TCGCCTCACACGCGAAAGGTCCCCGGTTCGAAACCGGGCGGAAACACC	48	0,966966636	17,1452717	16,8392015	17,247295
		tRF-49-W18FLUD3KS7SB1RHODE2	TCGCCTCACACGCGAAAGGTCCCCGGTTCGAAACCGGGCGGAAACACCA	49	0,951385867	63,1345493	62,8267171	63,23716
		tRF-49-YV45EHBK8RR9ODMJ6BFV	TTCGCCTCACACGCGAAAGGTCCCCGGTTCGAAACCGGGCGGAAACACC	49	ND	ND	ND	ND
		tRF-50-7S362VO0SRMNLB3KMB01	GTTCGCCTCACACGCGAAAGGTCCCCGGTTCGAAACCGGGCGGAAACACC	50	ND	ND	ND	ND
		tRF-50-YV45EHBK8RR9ODMJ6B26	TTCGCCTCACACGCGAAAGGTCCCCGGTTCGAAACCGGGCGGAAACACCA	50	0,963820905	20,5081088	20,9589361	20,357833

tRNA	Variant	tsRNA ID (MINTbase)	Sequence	Seq length	PCa_vs_BPH_pvalue	baseMean	BPH mean	PCa mean
Val-CAC	3'-tRF	tRF-25-R9ODMJ6B26	GGTTCGAAACCGGGCGGAAACACCA	25	0,001810681	462,268389	264,720287	528,117756
		tRF-26-MNLB3KMB01B	CGGTTCGAAACCGGGCGGAAACACCA	26	0,123387945	717,326589	544,63784	774,889505
		tRF-27-33WB08Q2B52	CCGGTTCGAAACCGGGCGGAAACACCA	27	0,415211519	307,534062	272,946657	319,063197
		tRF-27-Q3WB08Q2B52	GCGGTTCGAAACCGGGCGGAAACACCA	27	ND	ND	ND	ND
		tRF-28-K8YUBS68BFD2	CCCGGTTCGAAACCGGGCGGAAACACCA	28	0,474703334	8,86471654	12,4326418	7,67540811
		tRF-29-3H7SB1RHODE2	CCGCGGTTCGAAACCGGGCGGAAACACCA	29	ND	ND	ND	ND
		tRF-29-KS7SB1RHODE2	CCCCGGTTCGAAACCGGGCGGAAACACCA	29	0,394735405	11344,5536	13233,4384	10714,9253
		tRF-30-8RR9ODMJ6B26	TCCCCGGTTCGAAACCGGGCGGAAACACCA	30	0,706747778	8706,10914	9736,06966	8362,78897
		tRF-30-8VR9ODMJ6B26	TCCGCGGTTCGAAACCGGGCGGAAACACCA	30	ND	ND	ND	ND
		tRF-31-SRMNLB3KMB01B	GTCCCCGGTTCGAAACCGGGCGGAAACACCA	31	0,057070135	162,064796	111,749629	178,836518
		tRF-32-RR33WB08Q2B52	GGTCCCCGGTTCGAAACCGGGCGGAAACACCA	32	0,089993426	204,942603	151,792153	222,65942
		tRF-33-1MK8YUBS68BFD2	AGGTCCCCGGTTCGAAACCGGGCGGAAACACCA	33	0,305847305	199,734594	166,898205	210,680057

		tRF-34-D3KS7SB1RHODE2	AAGGTCCCCGGTTCGAAACCGGGCGGAAACACCA	34	0,000120658	222,247416	127,819628	253,723346
		tRF-35-BK8RR9ODMJ6B26	AAAGGTCCCCGGTTCGAAACCGGGCGGAAACACCA	35	0,163480374	303,887677	237,594915	325,985264
		tRF-36-O0SRMNLB3KMB01B	GAAAGGTCCCCGGTTCGAAACCGGGCGGAAACACCA	36	0,009413158	129,020567	74,4947248	147,195847
		tRF-36-U0SRMNLB3KMB01B	TAAAGGTCCCCGGTTCGAAACCGGGCGGAAACACCA	36	ND	ND	ND	ND
		tRF-37-LBRR33WB08Q2B52	CGAAAGGTCCCCGGTTCGAAACCGGGCGGAAACACCA	37	0,100939667	183,248764	123,91482	203,026746
		tRF-38-QB1MK8YUBS68BFD2	GCGAAAGGTCCCCGGTTCGAAACCGGGCGGAAACACCA	38	0,847495823	247,465431	265,009148	241,617525
		tRF-39-LUD3KS7SB1RHODE2	CGCGAAAGGTCCCCGGTTCGAAACCGGGCGGAAACACCA	39	0,838395385	210,846039	196,24112	215,714346
		tRF-40-EHBK8RR9ODMJ6B26	ACGCGAAAGGTCCCCGGTTCGAAACCGGGCGGAAACACCA	40	0,960820847	242,938901	231,610603	246,715001
		tRF-41-2VO0SRMNLB3KMB01B	CACGCGAAAGGTCCCCGGTTCGAAACCGGGCGGAAACACCA	41	0,238968201	297,164547	244,238681	314,806503
		tRF-42-0HLBRR33WB08Q2B52	ACACGCGAAAGGTCCCCGGTTCGAAACCGGGCGGAAACACCA	42	0,863272442	388,94059	347,42373	402,779544
		tRF-43-25QB1MK8YUBS68BFD2	CACACGCGAAAGGTCCCCGGTTCGAAACCGGGCGGAAACACCA	43	0,615187128	101,661375	107,922912	99,5741962
		tRF-43-B5QB1MK8YUBS68BFD2	AACACGCGAAAGGTCCCCGGTTCGAAACCGGGCGGAAACACCA	43	ND	ND	ND	ND
		tRF-44-8FLUD3KS7SB1RHODE2	TCACACGCGAAAGGTCCCCGGTTCGAAACCGGGCGGAAACACCA	44	0,680961177	63,7555257	66,857541	62,7215206
		tRF-45-45EHBK8RR9ODMJ6B26	CTCACACGCGAAAGGTCCCCGGTTCGAAACCGGGCGGAAACACCA	45	0,671962835	100,614012	92,0922037	103,454614
		tRF-45-4DEHBK8RR9ODMJ6B26	CTAACACGCGAAAGGTCCCCGGTTCGAAACCGGGCGGAAACACCA	45	ND	ND	ND	ND
		tRF-46-362VO0SRMNLB3KMB01B	CCTCACACGCGAAAGGTCCCCGGTTCGAAACCGGGCGGAAACACCA	46	0,164870231	299,011014	372,487803	274,518751
		tRF-46-3O2VO0SRMNLB3KMB01B	CCTAACACGCGAAAGGTCCCCGGTTCGAAACCGGGCGGAAACACCA	46	ND	ND	ND	ND
		tRF-47-P90HLBRR33WB08Q2B52	GCCTCACACGCGAAAGGTCCCCGGTTCGAAACCGGGCGGAAACACCA	47	0,900840754	166,787234	169,963502	165,728478
		tRF-48-L725QB1MK8YUBS68BFD2	CGCCTCACACGCGAAAGGTCCCCGGTTCGAAACCGGGCGGAAACACCA	48	0,982621627	139,537164	139,08775	139,686968
		tRF-49-W18FLUD3KS7SB1RHODE2	TCGCCTCACACGCGAAAGGTCCCCGGTTCGAAACCGGGCGGAAACACCA	49	0,951385867	63,1345493	62,8267171	63,23716
		tRF-50-YV45EHBK8RR9ODMJ6B26	TTCGCCTCACACGCGAAAGGTCCCCGGTTCGAAACCGGGCGGAAACACCA	50	0,963820905	20,5081088	20,9589361	20,357833
**tRNA**	**Variant**	**tsRNA ID (MINTbase)**	**Sequence**	**Seq length**	**PCa_vs_BPH_pvalue**	**baseMean**	**BPH mean**	**PCa mean**
Val-CAC	3'-tRF	tRF-34-D3KS7SB1RHODE2	AAGGTCCCCGGTTCGAAACCGGGCGGAAACACCA	34	0,000120658	222,247416	127,819628	253,723346
		tRF-35-BK8RR9ODMJ6B26	AAAGGTCCCCGGTTCGAAACCGGGCGGAAACACCA	35	0,163480374	303,887677	237,594915	325,985264
		tRF-36-O0SRMNLB3KMB01B	GAAAGGTCCCCGGTTCGAAACCGGGCGGAAACACCA	36	0,009413158	129,020567	74,4947248	147,195847
		tRF-36-U0SRMNLB3KMB01B	TAAAGGTCCCCGGTTCGAAACCGGGCGGAAACACCA	36	ND	ND	ND	ND
		tRF-37-LBRR33WB08Q2B52	CGAAAGGTCCCCGGTTCGAAACCGGGCGGAAACACCA	37	0,100939667	183,248764	123,91482	203,026746
		tRF-38-QB1MK8YUBS68BFD2	GCGAAAGGTCCCCGGTTCGAAACCGGGCGGAAACACCA	38	0,847495823	247,465431	265,009148	241,617525
		tRF-39-LUD3KS7SB1RHODE2	CGCGAAAGGTCCCCGGTTCGAAACCGGGCGGAAACACCA	39	0,838395385	210,846039	196,24112	215,714346
		tRF-40-EHBK8RR9ODMJ6B26	ACGCGAAAGGTCCCCGGTTCGAAACCGGGCGGAAACACCA	40	0,960820847	242,938901	231,610603	246,715001
		tRF-41-2VO0SRMNLB3KMB01B	CACGCGAAAGGTCCCCGGTTCGAAACCGGGCGGAAACACCA	41	0,238968201	297,164547	244,238681	314,806503
		tRF-42-0HLBRR33WB08Q2B52	ACACGCGAAAGGTCCCCGGTTCGAAACCGGGCGGAAACACCA	42	0,863272442	388,94059	347,42373	402,779544
		tRF-43-25QB1MK8YUBS68BFD2	CACACGCGAAAGGTCCCCGGTTCGAAACCGGGCGGAAACACCA	43	0,615187128	101,661375	107,922912	99,5741962
		tRF-43-B5QB1MK8YUBS68BFD2	AACACGCGAAAGGTCCCCGGTTCGAAACCGGGCGGAAACACCA	43	ND	ND	ND	ND
		tRF-44-8FLUD3KS7SB1RHODE2	TCACACGCGAAAGGTCCCCGGTTCGAAACCGGGCGGAAACACCA	44	0,680961177	63,7555257	66,857541	62,7215206
		tRF-45-45EHBK8RR9ODMJ6B26	CTCACACGCGAAAGGTCCCCGGTTCGAAACCGGGCGGAAACACCA	45	0,671962835	100,614012	92,0922037	103,454614
		tRF-45-4DEHBK8RR9ODMJ6B26	CTAACACGCGAAAGGTCCCCGGTTCGAAACCGGGCGGAAACACCA	45	ND	ND	ND	ND
		tRF-46-362VO0SRMNLB3KMB01B	CCTCACACGCGAAAGGTCCCCGGTTCGAAACCGGGCGGAAACACCA	46	0,164870231	299,011014	372,487803	274,518751
		tRF-46-3O2VO0SRMNLB3KMB01B	CCTAACACGCGAAAGGTCCCCGGTTCGAAACCGGGCGGAAACACCA	46	ND	ND	ND	ND
		tRF-47-P90HLBRR33WB08Q2B52	GCCTCACACGCGAAAGGTCCCCGGTTCGAAACCGGGCGGAAACACCA	47	0,900840754	166,787234	169,963502	165,728478
		tRF-48-L725QB1MK8YUBS68BFD2	CGCCTCACACGCGAAAGGTCCCCGGTTCGAAACCGGGCGGAAACACCA	48	0,982621627	139,537164	139,08775	139,686968
		tRF-49-W18FLUD3KS7SB1RHODE2	TCGCCTCACACGCGAAAGGTCCCCGGTTCGAAACCGGGCGGAAACACCA	49	0,951385867	63,1345493	62,8267171	63,23716
		tRF-50-YV45EHBK8RR9ODMJ6B26	TTCGCCTCACACGCGAAAGGTCCCCGGTTCGAAACCGGGCGGAAACACCA	50	0,963820905	20,5081088	20,9589361	20,357833
			**B. PB.PC vs BPH.PA**					
**tRNA**	**Variant**	**tsRNA ID (MINTbase)**	**Sequence**	**Seq length**	**PB.PC_vs_BPH.PA_pvalue**	**baseMean**	**BPH.PA mean**	**PB.PC mean**

Asn-GTT	3'-half	tRF-41-YDLBRY73WEK5KKOVD	TTAACCGAAAGGTTGGTGGTTCGATCCCACCCAGGGACGCC	41	0,002502845	140,484564	182,474218	98,494911
		tRF-42-7O3B1NR8YU86KP6HJ	GTTAACCGAAAGGTTGGTGGTTCGATCCCACCCAGGGACGCC	42	ND	ND	ND	ND
		tRF-42-YDLBRY73WEK5KKOV2	TTAACCGAAAGGTTGGTGGTTCGATCCCACCCAGGGACGCCA	42	0,36822474	43,211138	47,4887781	38,9334979
		tRF-43-7O3B1NR8YU86KP6HD2	GTTAACCGAAAGGTTGGTGGTTCGATCCCACCCAGGGACGCCA	43	ND	ND	ND	ND
		tRF-43-9X0UD3947SHR06RDDV	TGTTAACCGAAAGGTTGGTGGTTCGATCCCACCCAGGGACGCC	43	ND	ND	ND	ND
		tRF-44-9X0UD3947SHR06RDI2	TGTTAACCGAAAGGTTGGTGGTTCGATCCCACCCAGGGACGCCA	44	ND	ND	ND	ND
		tRF-44-NNBSBKZ3R95R2R121V	CTGTTAACCGAAAGGTTGGTGGTTCGATCCCACCCAGGGACGCC	44	0,003671405	5,33323365	2,99601137	7,67045592
		tRF-45-NNBSBKZ3R95R2R12L6	CTGTTAACCGAAAGGTTGGTGGTTCGATCCCACCCAGGGACGCCA	45	ND	ND	ND	ND
		tRF-45-QWU1O0789NLMK1JKE1	GCTGTTAACCGAAAGGTTGGTGGTTCGATCCCACCCAGGGACGCC	45	ND	ND	ND	ND
		tRF-46-6YYDLBRY73WEK5KKOVD	GGCTGTTAACCGAAAGGTTGGTGGTTCGATCCCACCCAGGGACGCC	46	ND	ND	ND	ND
		tRF-46-QWU1O0789NLMK1JKE1B	GCTGTTAACCGAAAGGTTGGTGGTTCGATCCCACCCAGGGACGCCA	46	ND	ND	ND	ND
		tRF-47-MI7O3B1NR8YU86KP6HJ	CGGCTGTTAACCGAAAGGTTGGTGGTTCGATCCCACCCAGGGACGCC	47	ND	ND	ND	ND
		tRF-48-MI7O3B1NR8YU86KP6HD2	CGGCTGTTAACCGAAAGGTTGGTGGTTCGATCCCACCCAGGGACGCCA	48	ND	ND	ND	ND
		tRF-48-WJ9X0UD3947SHR06RDDV	TCGGCTGTTAACCGAAAGGTTGGTGGTTCGATCCCACCCAGGGACGCC	48	ND	ND	ND	ND
		tRF-49-WJ9X0UD3947SHR06RDI2	TCGGCTGTTAACCGAAAGGTTGGTGGTTCGATCCCACCCAGGGACGCCA	49	ND	ND	ND	ND
		tRF-49-Y8NNBSBKZ3R95R2R121V	TTCGGCTGTTAACCGAAAGGTTGGTGGTTCGATCCCACCCAGGGACGCC	49	ND	ND	ND	ND
		tRF-50-Y8NNBSBKZ3R95R2R12L6	TTCGGCTGTTAACCGAAAGGTTGGTGGTTCGATCCCACCCAGGGACGCCA	50	ND	ND	ND	ND

tRNA	Variant	tsRNA ID (MINTbase)	Sequence	Seq length	PB.PC_vs_BPH.PA_pvalue	baseMean	BPH.PA mean	PB.PC mean
Asp-GTC	3'-half	tRF-41-8HM2OSRN2NKSEK51B	TCACGCGGGAGACCGGGGTTCAATTCCCCGACGGGGAGCCA	41	0,00085765	7786,16879	10492,7399	5079,59765
		tRF-45-3W2VR008R9D9KUMKF6	CCTGTCACGCGGGAGACCGGGGTTCAATTCCCCGACGGGGAGCCA	45	0,004487325	829,353411	1029,58639	629,120434
		tRF-46-PY8HM2OSRN2NKSEK51B	GCCTGTCACGCGGGAGACCGGGGTTCAATTCCCCGACGGGGAGCCA	46	0,062464015	954,905858	1132,66181	777,149902
		tRF-44-NMEH623K76IR3DR2I2	CTGTCACGCGGGAGACCGGGGTTCAATTCCCCGACGGGGAGCCA	44	0,003564752	949,708407	1168,61011	730,806708
		tRF-47-L7S5QKF1R38E8RO86J2	CGCCTGTCACGCGGGAGACCGGGGTTCAATTCCCCGACGGGGAGCCA	47	0,075227328	634,817229	749,479586	520,154871
		tRF-48-3196L85DMKYOYRLHR0D2	CCGCCTGTCACGCGGGAGACCGGGGTTCAATTCCCCGACGGGGAGCCA	48	0,050862864	535,934567	654,742121	417,127012
		tRF-43-96L85DMKYOYRLHR0D2	TGTCACGCGGGAGACCGGGGTTCAATTCCCCGACGGGGAGCCA	43	0,013961836	374,207029	454,063032	294,351026
		tRF-42-S5QKF1R38E8RO86J2	GTCACGCGGGAGACCGGGGTTCAATTCCCCGACGGGGAGCCA	42	0,090580993	416,055461	492,362094	339,748829
		tRF-50-KS3W2VR008R9D9KUMKF6	CCCCGCCTGTCACGCGGGAGACCGGGGTTCAATTCCCCGACGGGGAGCCA	50	0,254770961	174,753841	193,139565	156,368117
		tRF-49-KVNMEH623K76IR3DR2I2	CCCGCCTGTCACGCGGGAGACCGGGGTTCAATTCCCCGACGGGGAGCCA	49	0,251812807	122,601382	138,368074	106,834691

tRNA	Variant	tsRNA ID (MINTbase)	Sequence	Seq length	PB.PC_vs_BPH.PA_pvalue	baseMean	BPH.PA mean	PB.PC mean
Asp-GTC	3'-half	tRF-41-8HM2OSRNLNKSEK51B	TCACGCGGGAGACCGGGGTTCGATTCCCCGACGGGGAGCCA	41	0,001829777	40599,0536	54534,2183	26663,8889
		tRF-45-3W2VR008R959KUMKF6	CCTGTCACGCGGGAGACCGGGGTTCGATTCCCCGACGGGGAGCCA	45	0,000944981	8024,84073	10895,5554	5154,12608
		tRF-44-NMEH623K7SIR3DR2I2	CTGTCACGCGGGAGACCGGGGTTCGATTCCCCGACGGGGAGCCA	44	0,011711852	6531,36398	8127,38107	4935,3469
		tRF-46-PY8HM2OSRNLNKSEK51B	GCCTGTCACGCGGGAGACCGGGGTTCGATTCCCCGACGGGGAGCCA	46	0,061956168	6296,95805	7604,22406	4989,69204
		tRF-47-L7S5QKF1R3WE8RO86J2	CGCCTGTCACGCGGGAGACCGGGGTTCGATTCCCCGACGGGGAGCCA	47	0,051044226	4600,0464	5579,4291	3620,6637
		tRF-48-3196L85DMKYUYRLHR0D2	CCGCCTGTCACGCGGGAGACCGGGGTTCGATTCCCCGACGGGGAGCCA	48	0,028207132	3689,18503	4658,53095	2719,83911
		tRF-42-S5QKF1R3WE8RO86J2	GTCACGCGGGAGACCGGGGTTCGATTCCCCGACGGGGAGCCA	42	0,070677729	2501,30568	2959,30041	2043,31095
		tRF-43-96L85DMKYUYRLHR0D2	TGTCACGCGGGAGACCGGGGTTCGATTCCCCGACGGGGAGCCA	43	0,024782691	2336,42834	2851,5638	1821,29288
		tRF-50-KS3W2VR008R959KUMKF6	CCCCGCCTGTCACGCGGGAGACCGGGGTTCGATTCCCCGACGGGGAGCCA	50	0,19502682	1803,82573	2026,39951	1581,25196
		tRF-49-KVNMEH623K7SIR3DR2I2	CCCGCCTGTCACGCGGGAGACCGGGGTTCGATTCCCCGACGGGGAGCCA	49	0,035923384	1139,29472	1364,24603	914,343406
		tRF-45-WW2VR008R959KUMKF6	TCTGTCACGCGGGAGACCGGGGTTCGATTCCCCGACGGGGAGCCA	45	0,000755877	30,7995647	42,3323481	19,2667813
		tRF-48-3M96L85DMKYUYRLHR0D2	CCGTCTGTCACGCGGGAGACCGGGGTTCGATTCCCCGACGGGGAGCCA	48	ND	ND	ND	ND
		tRF-46-SY8HM2OSRNLNKSEK51B	GTCTGTCACGCGGGAGACCGGGGTTCGATTCCCCGACGGGGAGCCA	46	ND	ND	ND	ND
		tRF-47-M7S5QKF1R3WE8RO86J2	CGTCTGTCACGCGGGAGACCGGGGTTCGATTCCCCGACGGGGAGCCA	47	ND	ND	ND	ND

tRNA	Variant	tsRNA ID (MINTbase)	Sequence	Seq length	PB.PC_vs_BPH.PA_pvalue	baseMean	BPH.PA mean	PB.PC mean
Asp-GTC	3'-tRF	tRF-43-3W2VR008R959KUMK9	CCTGTCACGCGGGAGACCGGGGTTCGATTCCCCGACGGGGAGC	43	0,003933066	321,348526	429,722102	212,97495
		tRF-44-3W2VR008R959KUMKEV	CCTGTCACGCGGGAGACCGGGGTTCGATTCCCCGACGGGGAGCC	44	0,024142983	855,244196	1074,61193	635,876459
		tRF-44-PY8HM2OSRNLNKSEKH9	GCCTGTCACGCGGGAGACCGGGGTTCGATTCCCCGACGGGGAGC	44	0,274972164	541,781666	633,915947	449,647386
		tRF-45-3W2VR008R959KUMKF6	CCTGTCACGCGGGAGACCGGGGTTCGATTCCCCGACGGGGAGCCA	45	0,000944981	8024,84073	10895,5554	5154,12608
		tRF-45-L7S5QKF1R3WE8RO86J	CGCCTGTCACGCGGGAGACCGGGGTTCGATTCCCCGACGGGGAGC	45	0,130694926	182,44855	221,86769	143,029411
		tRF-45-PY8HM2OSRNLNKSEK51	GCCTGTCACGCGGGAGACCGGGGTTCGATTCCCCGACGGGGAGCC	45	0,120904761	1533,83682	1862,3239	1205,34973
		tRF-46-3196L85DMKYUYRLHR0D	CCGCCTGTCACGCGGGAGACCGGGGTTCGATTCCCCGACGGGGAGC	46	0,072971	135,170553	169,537328	100,803777
		tRF-46-L7S5QKF1R3WE8RO86JD	CGCCTGTCACGCGGGAGACCGGGGTTCGATTCCCCGACGGGGAGCC	46	0,265463041	271,877387	318,721826	225,032948
		tRF-46-PY8HM2OSRNLNKSEK51B	GCCTGTCACGCGGGAGACCGGGGTTCGATTCCCCGACGGGGAGCCA	46	0,061956168	6296,95805	7604,22406	4989,69204
		tRF-47-3196L85DMKYUYRLHR0J	CCGCCTGTCACGCGGGAGACCGGGGTTCGATTCCCCGACGGGGAGCC	47	0,057778657	404,887912	513,647705	296,128118
		tRF-47-KVNMEH623K7SIR3DR2M	CCCGCCTGTCACGCGGGAGACCGGGGTTCGATTCCCCGACGGGGAGC	47	0,397188519	48,3394819	54,061462	42,6175017
		tRF-47-L7S5QKF1R3WE8RO86J2	CGCCTGTCACGCGGGAGACCGGGGTTCGATTCCCCGACGGGGAGCCA	47	0,051044226	4600,0464	5579,4291	3620,6637
		tRF-48-3196L85DMKYUYRLHR0D2	CCGCCTGTCACGCGGGAGACCGGGGTTCGATTCCCCGACGGGGAGCCA	48	0,028207132	3689,18503	4658,53095	2719,83911
		tRF-48-KS3W2VR008R959KUMK9	CCCCGCCTGTCACGCGGGAGACCGGGGTTCGATTCCCCGACGGGGAGC	48	0,580008058	73,3450077	81,266366	65,4236494
		tRF-48-KVNMEH623K7SIR3DR2DV	CCCGCCTGTCACGCGGGAGACCGGGGTTCGATTCCCCGACGGGGAGCC	48	0,08137081	145,235293	178,873327	111,59726
		tRF-49-8RPY8HM2OSRNLNKSEKH9	TCCCCGCCTGTCACGCGGGAGACCGGGGTTCGATTCCCCGACGGGGAGC	49	0,119830758	52,7715643	63,8190335	41,7240951
		tRF-49-KS3W2VR008R959KUMKEV	CCCCGCCTGTCACGCGGGAGACCGGGGTTCGATTCCCCGACGGGGAGCC	49	0,131092116	395,52904	475,443241	315,614838
		tRF-49-KVNMEH623K7SIR3DR2I2	CCCGCCTGTCACGCGGGAGACCGGGGTTCGATTCCCCGACGGGGAGCCA	49	0,035923384	1139,29472	1364,24603	914,343406
		tRF-50-8RPY8HM2OSRNLNKSEK51	TCCCCGCCTGTCACGCGGGAGACCGGGGTTCGATTCCCCGACGGGGAGCC	50	0,098480484	131,457434	157,904015	105,010854
		tRF-50-HRL7S5QKF1R3WE8RO86J	ATCCCCGCCTGTCACGCGGGAGACCGGGGTTCGATTCCCCGACGGGGAGC	50	0,772336059	53,4530864	55,6951799	51,2109928
		tRF-50-KS3W2VR008R959KUMKF6	CCCCGCCTGTCACGCGGGAGACCGGGGTTCGATTCCCCGACGGGGAGCCA	50	0,19502682	1803,82573	2026,39951	1581,25196

tRNA	Variant	tsRNA ID (MINTbase)	Sequence	Seq length	PB.PC_vs_BPH.PA_pvalue	baseMean	BPH.PA mean	PB.PC mean
Asp-GTC	3'-tRF	tRF-44-NMEH623K76IR3DR2I2	CTGTCACGCGGGAGACCGGGGTTCAATTCCCCGACGGGGAGCCA	44	0,003564752	949,708407	1168,61011	730,806708
		tRF-45-3W2VR008R9D9KUMKF6	CCTGTCACGCGGGAGACCGGGGTTCAATTCCCCGACGGGGAGCCA	45	0,004487325	829,353411	1029,58639	629,120434
		tRF-46-PY8HM2OSRN2NKSEK51B	GCCTGTCACGCGGGAGACCGGGGTTCAATTCCCCGACGGGGAGCCA	46	0,062464015	954,905858	1132,66181	777,149902
		tRF-47-L7S5QKF1R38E8RO86J2	CGCCTGTCACGCGGGAGACCGGGGTTCAATTCCCCGACGGGGAGCCA	47	0,075227328	634,817229	749,479586	520,154871
		tRF-48-3196L85DMKYOYRLHR0D2	CCGCCTGTCACGCGGGAGACCGGGGTTCAATTCCCCGACGGGGAGCCA	48	0,050862864	535,934567	654,742121	417,127012
		tRF-49-KVNMEH623K76IR3DR2I2	CCCGCCTGTCACGCGGGAGACCGGGGTTCAATTCCCCGACGGGGAGCCA	49	0,251812807	122,601382	138,368074	106,834691
		tRF-50-KS3W2VR008R9D9KUMKF6	CCCCGCCTGTCACGCGGGAGACCGGGGTTCAATTCCCCGACGGGGAGCCA	50	0,254770961	174,753841	193,139565	156,368117

tRNA	Variant	tsRNA ID (MINTbase)	Sequence	Seq length	PB.PC_vs_BPH.PA_pvalue	baseMean	BPH.PA mean	PB.PC mean
Asp-GTC	3'-tRF	tRF-45-3W2VR008R959KUMKF6	CCTGTCACGCGGGAGACCGGGGTTCGATTCCCCGACGGGGAGCCA	45	0,000944981	8024,84073	10895,5554	5154,12608
		tRF-46-PY8HM2OSRNLNKSEK51B	GCCTGTCACGCGGGAGACCGGGGTTCGATTCCCCGACGGGGAGCCA	46	0,061956168	6296,95805	7604,22406	4989,69204
		tRF-47-L7S5QKF1R3WE8RO86J2	CGCCTGTCACGCGGGAGACCGGGGTTCGATTCCCCGACGGGGAGCCA	47	0,051044226	4600,0464	5579,4291	3620,6637
		tRF-48-3196L85DMKYUYRLHR0D2	CCGCCTGTCACGCGGGAGACCGGGGTTCGATTCCCCGACGGGGAGCCA	48	0,028207132	3689,18503	4658,53095	2719,83911
		tRF-49-KVNMEH623K7SIR3DR2I2	CCCGCCTGTCACGCGGGAGACCGGGGTTCGATTCCCCGACGGGGAGCCA	49	0,035923384	1139,29472	1364,24603	914,343406
		tRF-50-KS3W2VR008R959KUMKF6	CCCCGCCTGTCACGCGGGAGACCGGGGTTCGATTCCCCGACGGGGAGCCA	50	0,19502682	1803,82573	2026,39951	1581,25196

tRNA	Variant	tsRNA ID (MINTbase)	Sequence	Seq length	PB.PC_vs_BPH.PA_pvalue	baseMean	BPH.PA mean	PB.PC mean
Asp-GTC	3'-tRF	tRF-45-3W2VR008R9D9KUMKF6	CCTGTCACGCGGGAGACCGGGGTTCAATTCCCCGACGGGGAGCCA	45	0,004487325	829,353411	1029,58639	629,120434
		tRF-46-PY8HM2OSRN2NKSEK51B	GCCTGTCACGCGGGAGACCGGGGTTCAATTCCCCGACGGGGAGCCA	46	0,062464015	954,905858	1132,66181	777,149902
		tRF-47-L7S5QKF1R38E8RO86J2	CGCCTGTCACGCGGGAGACCGGGGTTCAATTCCCCGACGGGGAGCCA	47	0,075227328	634,817229	749,479586	520,154871
		tRF-48-3196L85DMKYOYRLHR0D2	CCGCCTGTCACGCGGGAGACCGGGGTTCAATTCCCCGACGGGGAGCCA	48	0,050862864	535,934567	654,742121	417,127012
		tRF-49-KVNMEH623K76IR3DR2I2	CCCGCCTGTCACGCGGGAGACCGGGGTTCAATTCCCCGACGGGGAGCCA	49	0,251812807	122,601382	138,368074	106,834691
		tRF-50-KS3W2VR008R9D9KUMKF6	CCCCGCCTGTCACGCGGGAGACCGGGGTTCAATTCCCCGACGGGGAGCCA	50	0,254770961	174,753841	193,139565	156,368117

tRNA	Variant	tsRNA ID (MINTbase)	Sequence	Seq length	PB.PC_vs_BPH.PA_pvalue	baseMean	BPH.PA mean	PB.PC mean
Glu-TTC	3'-tRF	tRF-43-7Z2R1HPSR9O9337KR	GTTTTCACCCAGGCGGCCCGGGTTCGACTCCCGGTGTGGGAAC	43	0,00399357	168,963978	225,46604	112,461916
		tRF-44-7Z2R1HPSR9O9337K0V	GTTTTCACCCAGGCGGCCCGGGTTCGACTCCCGGTGTGGGAACC	44	0,002310351	520,449064	706,971129	333,926999
		tRF-44-RZ81JJ6RRNLIK898HR	GGTTTTCACCCAGGCGGCCCGGGTTCGACTCCCGGTGTGGGAAC	44	0,167924218	165,115939	205,991725	124,240153
		tRF-45-7Z2R1HPSR9O9337KB6	GTTTTCACCCAGGCGGCCCGGGTTCGACTCCCGGTGTGGGAACCA	45	0,000196811	1452,14787	2060,74104	843,554701
		tRF-45-9NY5KKM1M3WD8S746D	TGGTTTTCACCCAGGCGGCCCGGGTTCGACTCCCGGTGTGGGAAC	45	0,605191879	48,3645127	55,7620694	40,9669561
		tRF-45-RZ81JJ6RRNLIK898O1	GGTTTTCACCCAGGCGGCCCGGGTTCGACTCCCGGTGTGGGAACC	45	0,036819415	117,565817	149,444073	85,6875605
		tRF-46-9NY5KKM1M3WD8S746DD	TGGTTTTCACCCAGGCGGCCCGGGTTCGACTCCCGGTGTGGGAACC	46	0,156841273	30,5739693	37,4584844	23,6894542
		tRF-46-N3Z6KPQJ3KYU4RRWRBD	CTGGTTTTCACCCAGGCGGCCCGGGTTCGACTCCCGGTGTGGGAAC	46	0,443219592	60,4159984	69,5113275	51,3206693
		tRF-46-RZ81JJ6RRNLIK898O1B	GGTTTTCACCCAGGCGGCCCGGGTTCGACTCCCGGTGTGGGAACCA	46	0,002876386	98,6442445	126,925236	70,3632524
		tRF-47-38Z90668K87SERM4921	CCTGGTTTTCACCCAGGCGGCCCGGGTTCGACTCCCGGTGTGGGAAC	47	0,351780737	49,9798592	56,2286649	43,7310535
		tRF-47-9NY5KKM1M3WD8S746D2	TGGTTTTCACCCAGGCGGCCCGGGTTCGACTCCCGGTGTGGGAACCA	47	0,020877246	62,6220236	78,9968977	46,2471496
		tRF-47-N3Z6KPQJ3KYU4RRWRBJ	CTGGTTTTCACCCAGGCGGCCCGGGTTCGACTCCCGGTGTGGGAACC	47	0,012613651	239,169272	304,122632	174,215912
		tRF-48-38Z90668K87SERM492V	CCTGGTTTTCACCCAGGCGGCCCGGGTTCGACTCCCGGTGTGGGAACC	48	0,137732476	242,104545	291,212519	192,99657
		tRF-48-8Y7Z2R1HPSR9O9337KR	TCCTGGTTTTCACCCAGGCGGCCCGGGTTCGACTCCCGGTGTGGGAAC	48	0,284499085	144,452125	170,795345	118,108905
		tRF-48-N3Z6KPQJ3KYU4RRWRBD2	CTGGTTTTCACCCAGGCGGCCCGGGTTCGACTCCCGGTGTGGGAACCA	48	0,021498259	569,742643	723,225122	416,260164
		tRF-49-38Z90668K87SERM492E2	CCTGGTTTTCACCCAGGCGGCCCGGGTTCGACTCCCGGTGTGGGAACCA	49	0,014130898	781,422503	955,96439	606,880615
		tRF-49-8Y7Z2R1HPSR9O9337K0V	TCCTGGTTTTCACCCAGGCGGCCCGGGTTCGACTCCCGGTGTGGGAACC	49	0,058912646	409,118749	519,399846	298,837651
		tRF-49-Y7RZ81JJ6RRNLIK898HR	TTCCTGGTTTTCACCCAGGCGGCCCGGGTTCGACTCCCGGTGTGGGAAC	49	0,231258825	154,174028	180,492198	127,855859
		tRF-50-8Y7Z2R1HPSR9O9337KB6	TCCTGGTTTTCACCCAGGCGGCCCGGGTTCGACTCCCGGTGTGGGAACCA	50	0,044840294	1982,86481	2460,84015	1504,88946
		tRF-50-IR9NY5KKM1M3WD8S746D	ATTCCTGGTTTTCACCCAGGCGGCCCGGGTTCGACTCCCGGTGTGGGAAC	50	0,468196463	179,041874	205,629373	152,454375
		tRF-50-Y7RZ81JJ6RRNLIK898O1	TTCCTGGTTTTCACCCAGGCGGCCCGGGTTCGACTCCCGGTGTGGGAACC	50	0,24945557	491,584737	587,958237	395,211237

tRNA	Variant	tsRNA ID (MINTbase)	Sequence	Seq length	PB.PC_vs_BPH.PA_pvalue	baseMean	BPH.PA mean	PB.PC mean
Glu-TTC	3'-tRF	tRF-44-7Z2R1HPSR9O9337K0V	GTTTTCACCCAGGCGGCCCGGGTTCGACTCCCGGTGTGGGAACC	44	0,002310351	520,449064	706,971129	333,926999
		tRF-45-7Z2R1HPSR9O9337KB6	GTTTTCACCCAGGCGGCCCGGGTTCGACTCCCGGTGTGGGAACCA	45	0,000196811	1452,14787	2060,74104	843,554701
		tRF-45-RZ81JJ6RRNLIK898O1	GGTTTTCACCCAGGCGGCCCGGGTTCGACTCCCGGTGTGGGAACC	45	0,036819415	117,565817	149,444073	85,6875605
		tRF-46-9NY5KKM1M3WD8S746DD	TGGTTTTCACCCAGGCGGCCCGGGTTCGACTCCCGGTGTGGGAACC	46	0,156841273	30,5739693	37,4584844	23,6894542
		tRF-46-RZ81JJ6RRNLIK898O1B	GGTTTTCACCCAGGCGGCCCGGGTTCGACTCCCGGTGTGGGAACCA	46	0,002876386	98,6442445	126,925236	70,3632524
		tRF-47-9NY5KKM1M3WD8S746D2	TGGTTTTCACCCAGGCGGCCCGGGTTCGACTCCCGGTGTGGGAACCA	47	0,020877246	62,6220236	78,9968977	46,2471496
		tRF-47-N3Z6KPQJ3KYU4RRWRBJ	CTGGTTTTCACCCAGGCGGCCCGGGTTCGACTCCCGGTGTGGGAACC	47	0,012613651	239,169272	304,122632	174,215912
		tRF-48-38Z90668K87SERM492V	CCTGGTTTTCACCCAGGCGGCCCGGGTTCGACTCCCGGTGTGGGAACC	48	0,137732476	242,104545	291,212519	192,99657
		tRF-48-N3Z6KPQJ3KYU4RRWRBD2	CTGGTTTTCACCCAGGCGGCCCGGGTTCGACTCCCGGTGTGGGAACCA	48	0,021498259	569,742643	723,225122	416,260164
		tRF-49-38Z90668K87SERM492E2	CCTGGTTTTCACCCAGGCGGCCCGGGTTCGACTCCCGGTGTGGGAACCA	49	0,014130898	781,422503	955,96439	606,880615
		tRF-49-8Y7Z2R1HPSR9O9337K0V	TCCTGGTTTTCACCCAGGCGGCCCGGGTTCGACTCCCGGTGTGGGAACC	49	0,058912646	409,118749	519,399846	298,837651
		tRF-50-8Y7Z2R1HPSR9O9337KB6	TCCTGGTTTTCACCCAGGCGGCCCGGGTTCGACTCCCGGTGTGGGAACCA	50	0,044840294	1982,86481	2460,84015	1504,88946
		tRF-50-Y7RZ81JJ6RRNLIK898O1	TTCCTGGTTTTCACCCAGGCGGCCCGGGTTCGACTCCCGGTGTGGGAACC	50	0,24945557	491,584737	587,958237	395,211237

tRNA	Variant	tsRNA ID (MINTbase)	Sequence	Seq length	PB.PC_vs_BPH.PA_pvalue	baseMean	BPH.PA mean	PB.PC mean
Glu-TTC	3'-tRF	tRF-45-7Z2R1HPSR9O9337KB6	GTTTTCACCCAGGCGGCCCGGGTTCGACTCCCGGTGTGGGAACCA	45	0,000196811	1452,14787	2060,74104	843,554701
		tRF-46-RZ81JJ6RRNLIK898O1B	GGTTTTCACCCAGGCGGCCCGGGTTCGACTCCCGGTGTGGGAACCA	46	0,002876386	98,6442445	126,925236	70,3632524
		tRF-47-9NY5KKM1M3WD8S746D2	TGGTTTTCACCCAGGCGGCCCGGGTTCGACTCCCGGTGTGGGAACCA	47	0,020877246	62,6220236	78,9968977	46,2471496
		tRF-48-N3Z6KPQJ3KYU4RRWRBD2	CTGGTTTTCACCCAGGCGGCCCGGGTTCGACTCCCGGTGTGGGAACCA	48	0,021498259	569,742643	723,225122	416,260164
		tRF-49-38Z90668K87SERM492E2	CCTGGTTTTCACCCAGGCGGCCCGGGTTCGACTCCCGGTGTGGGAACCA	49	0,014130898	781,422503	955,96439	606,880615
		tRF-50-8Y7Z2R1HPSR9O9337KB6	TCCTGGTTTTCACCCAGGCGGCCCGGGTTCGACTCCCGGTGTGGGAACCA	50	0,044840294	1982,86481	2460,84015	1504,88946

tsRNAs selected for RT-qPCR primer designing are depicted in blue. Those tsRNA variants presenting more NGS counts that the preselected isoform are depicted in yellow.

Supplementary Table S4. Target genes of semen sEV differentially expressed isomiRs related to prostate cancer signalling (KEGG)

A. PCa vs BPH							
**Target Gene**	**miRNA**	**miRNet**	**TargetScan**	**miRDB**	**Ensembl ID (Human Gene)**	**Description**	**Molecular function**
MTOR	hsa-miR-100-5p	Yes	Yes	Yes	ENSG00000198793	mechanistic target of rapamycin kinase [KO:K07203] [EC:2.7.11.1]	Mediates cellular responses to stress such as DNA damage and nutrient deprivation
	hsa-miR-193a-5p	Yes					
	hsa-miR-181a-2-3p		Yes				
IGF1R	hsa-miR-100-5p	Yes			ENSG00000140443	insulin like growth factor 1 receptor [KO:K05087] [EC:2.7.10.1]	This receptor binds insulin-like growth factor with a high affinity. It has tyrosine kinase activity. The IGF1R plays a critical role in transformation events
	hsa-miR-181a-2-3p		Yes	Yes			
MDM2	hsa-miR-221-3p	Yes			ENSG00000135679	MDM2 proto-oncogene [KO:K26116]	Nuclear E3 ubiquitin ligase that targets p53 tumor suppressor for proteasomal degradation, promoting tumor formation
	hsa-miR-181a-2-3p		Yes				
RB1	hsa-miR-100-5p	Yes			ENSG00000139687	RB transcriptional corepressor 1	Tumor suppressor protein that negatively regulates cell cycle progression by binding E2F1 and stabilizing heterochromatin
	hsa-miR-221-3p	Yes					
CREBBP	hsa-miR-100-5p	Yes			ENSG00000005339	CREB binding protein [KO:K04498] [EC:2.3.1.48]	Involved in the transcriptional coactivation of many different transcription factors
AKT1	hsa-miR-100-5p	Yes			ENSG00000142208	AKT serine/threonine kinase 1 [KO:K04456] [EC:2.7.11.1]	Serine/threonine kinase activated by PI3K that regulates cell proliferation, survival, metabolism, and angiogenesis; key component of oncogenic PI3K/AKT/mTOR signaling pathway
GRB2	hsa-miR-100-5p	Yes			ENSG00000177885	growth factor receptor bound protein 2 [KO:K04364]	Binds the epidermal growth factor receptor and contains one SH2 domain and two SH3 domains that direct complex formation with proline-rich regions of other proteins, linking EGFR to signal transduction
KRAS	hsa-miR-181a-2-3p		Yes	Yes	ENSG00000133703	KRAS proto-oncogene, GTPase [KO:K07827]	Member of the small GTPase superfamily. Proto-oncogene
NRAS	hsa-miR-181a-2-3p		Yes	Yes	ENSG00000213281	NRAS proto-oncogene, GTPase [KO:K07828]	N-ras oncogene encoding a membrane protein that shuttles between the Golgi apparatus and the plasma membrane
SLC46A1	hsa-miR-181a-2-3p		Yes		ENSG00000076351	solute carrier family 46 member 1 [KO:K14613]	Proton-coupled folate transporter protein that facilitates the movement of folate and antifolate substrates across cell membranes, optimally in acidic pH environments
LEF1	hsa-miR-181a-2-3p		Yes		ENSG00000138795	lymphoid enhancer binding factor 1 [KO:K04492]	Transcription factor involved in Wnt signaling pathway
BCL2	hsa-miR-181a-2-3p		Yes		ENSG00000171791	BCL2 apoptosis regulator [KO:K02161]	Involved in the inhibition of apoptosis
TCF7L1	hsa-miR-181a-2-3p		Yes		ENSG00000152284	transcription factor 7 like 1 [KO:K04490]	Transcription factor activated by beta-catenin to mediate Wnt signaling pathway and regulate cell cycle genes and cellular senescence
CASP9	hsa-miR-181a-2-3p		Yes		ENSG00000132906	caspase 9 [KO:K04399] [EC:3.4.22.62]	Plays a central role in apoptosis. Tumor suppressor
PIK3R3	hsa-miR-193a-5p	Yes			ENSG00000117461	phosphoinositide-3-kinase regulatory subunit 3 [KO:K02649]	Regulatory subunit of PI3K containing SH2 domains that bind activated protein tyrosine kinases to regulate growth signaling pathways
CREB3L3	hsa-miR-193a-5p			Yes	ENSG00000060566	cyclic AMP-responsive element-binding protein 3 [KO:K09048]	Binds the cyclic AMP response element (CRE) and the box-B element; transcription factor activity
CDKN1A	hsa-miR-193a-5p	Yes			ENSG00000124762	cyclin dependent kinase inhibitor 1A [KO:K06625]	Potent cyclin-dependent kinase inhibitor that binds and inhibits cyclin-CDK2 and cyclin-CDK4 complexes, regulating G1 cell cycle progression and mediating p53-dependent G1 arrest in response to stress stimuli
CREB3L2	hsa-miR-193a-5p			Yes	ENSG00000182158	cAMP responsive element binding protein 3 like 2 [KO:K09048]	Trancriptional activator
ERBB2	hsa-miR-193a-5p	Yes		Yes	ENSG00000141736	erb-b2 receptor tyrosine kinase 2 [KO:K05083] [EC:2.7.10.1]	Binds tightly to other ligand-bound EGF receptor family members to form a heterodimer, and enhancing kinase-mediated activation of downstream signalling pathways
PTEN	hsa-miR-221-3p	Yes			ENSG00000171862	phosphatase and tensin homolog [KO:K01110] [EC:3.1.3.16 3.1.3.48 3.1.3.67]	Negatively regulates AKT/PKB signaling by dephosphorylating phosphoinositide substrates, acting as a tumor suppressor

PIK3R1	hsa-miR-221-3p	Yes	Yes	Yes	ENSG00000145675	phosphoinositide-3-kinase regulatory subunit 1 [KO:K02649]	Regulatory subunit of phosphatidylinositol 3-kinase involved in phosphatidylinositol signaling and insulin-mediated metabolic actions
PDGFA	hsa-miR-221-3p	Yes	Yes		ENSG00000197461	platelet derived growth factor subunit A [KO:K04359]	Encodes platelet-derived growth factor subunit A that homodimerizes or heterodimerizes with subunit B to activate PDGF receptor tyrosine kinases, regulating developmental processes
CDKN1B	hsa-miR-221-3p	Yes		Yes	ENSG00000111276	cyclin dependent kinase inhibitor 1B [KO:K06624]	Cyclin-dependent kinase inhibitor that blocks activation of cyclin E-CDK2 and cyclin D-CDK4 complexes, controlling G1 cell cycle progression
CREB1	hsa-miR-221-3p	Yes			ENSG00000118260	cAMP responsive element binding protein 1 [KO:K05870]	Trancription factor that induces transcription of genes in response to hormonal stimulation of the cAMP pathway
TP53	hsa-miR-221-3p	Yes			ENSG00000141510	tumor protein p53 [KO:K16823]	Tumor suppressor protein that responds to cellular stress and regulates genes involved in cell cycle arrest, apoptosis, senescence, DNA repair, and metabolic changes
TCF7L2	hsa-miR-221-3p			Yes	ENSG00000148737	transcription factor 7 like 2 [KO:K04491]	Transcription factor involved in the Wnt signalling pathway
AKT3	hsa-miR-221-3p	Yes			ENSG00000117020	AKT serine/threonine kinase 3 [KO:K04456] [EC:2.7.11.1]	Serine/threonine kinase that regulates cell signaling in response to insulin and growth factors
CTNNB1	hsa-miR-221-3p	Yes			ENSG00000168036	catenin beta 1 [KO:K02105]	Component of adherens junctions that regulates epithelial cell growth and adhesion, anchors actin cytoskeleton and transmits contact inhibition signals
E2F3	hsa-miR-221-3p	Yes			ENSG00000112242	E2F transcription factor 3 [KO:K06620]	Transcription factor that binds DP proteins and interacts with retinoblastoma protein (pRB) to regulate cell cycle gene expression
**B. PB.PC vs BPH.PA**							
PIK3R1	hsa-miR-107			Yes	ENSG00000145675	phosphoinositide-3-kinase regulatory subunit 1 [KO:K02649]	Regulatory subunit of phosphatidylinositol 3-kinase involved in phosphatidylinositol signaling and insulin-mediated metabolic actions
AKT2	hsa-miR-107			Yes	ENSG00000105221	AKT serine/threonine kinase 2 [KO:K04456] [EC:2.7.11.1]	Protein kinase involved in signalling pathways as oncogene

Predicted miRNA–target interactions were identified using miRNet, TargetScan and miRDB; “Yes” indicates support by the corresponding prediction database. Ensembl IDs refer to human genes. KEGG Orthology (KO) and Enzyme Nomenclature (EC) numbers are reported when available to indicate pathway assignment and enzymatic activity.

Supplementary Table S5. Target genes of semen sEV differentially expressed tsRNAs related to prostate cancer signalling (KEGG).

**A. PCa vs BPH**							
**Target Gene**	**tsRNA ID (MINTBase)**	**tRFtar**	**RNAHybrid**	**miRanda**	**Ensembl ID (Human Gene)**	**Description**	**Molecular function**
MAPK3	tRF-21-LMKPJ7K1B	Yes			ENSG00000102882	mitogen-activated protein kinase 3 [KO:K04371] [EC:2.7.11.24]	MAP kinase involved in signaling cascades that regulate proliferation, differentiation, and cell cycle progression in response to extracellular signals
	tRF-27-M2WEPSJR852		Yes				
	tRF-19-F8DHXYIV		Yes				
FOLR2	tRF-21-LMKPJ7K1B		Yes		ENSG00000165457	folate receptor beta [KO:K13649]	High-affinity folate receptor that mediates delivery of 5-methyltetrahydrofolate into cells and transports methotrexate in synovial macrophages
	tRF-19-F8DHXYIV		Yes				
PDPK1	tRF-19-F8DHXYIV		Yes		ENSG00000140992	3-phosphoinositide dependent protein kinase 1 [KO:K06276] [EC:2.7.11.1]	3-phosphoinositide-dependent protein kinase, phospholipase activator activity and phospholipase binding activity. acts upstream of intracellular signal transduction, regulating cell surface receptor signaling pathways and implicated in prostate cancer progression
	tRF-25-R9ODMJ6B26		Yes				
IKBKB	tRF-40-8HM2OSRN2NKSEK51			Yes	ENSG00000104365	inhibitor of nuclear factor kappa B kinase subunit beta [KO:K07209] [EC:2.7.11.10]	Phosphorylates the inhibitor in the inhibitor/NF-kappa-B complex, causing dissociation of the inhibitor and activation of NF-kappa-B
	tRF-41-8HM2OSRN2NKSEK51B			Yes			
	tRF-38-8HM2OSRN2NKSEKDS			Yes			
CDK2	tRF-19-F8DHXYIV	Yes			ENSG00000123374	cyclin dependent kinase 2 [KO:K02206] [EC:2.7.11.22]	Catalytic subunit of cyclin-dependent kinase complex that regulates progression through the cell cycle
	tRF-34-D3KS7SB1RHODE2			Yes			
TCF7	tRF-19-F8DHXYIV		Yes		ENSG00000081059	transcription factor 7 [KO:K02620]	HMG-box transcription factor expressed in T-cells; activates Wnt/β-catenin signaling via β-catenin complex; critical for NK/ILC development
SLC19A1	tRF-19-F8DHXYIV		Yes		ENSG00000173638	solute carrier family 19 member 1 [KO:K14609]	Membrane transporter that regulates intracellular folate concentrations by facilitating folate uptake
IKBKG	tRF-19-F8DHXYIV		Yes		ENSG00000269335	inhibitor of nuclear factor kappa B kinase regulatory subunit gamma [KO:K07210]	Regulatory subunit of IKK complex; activates NF-κB for inflammation/immunity/cell survival gene expression
AKT1	tRF-19-F8DHXYIV	Yes			ENSG00000142208	AKT serine/threonine kinase 1 [KO:K04456] [EC:2.7.11.1]	Serine/threonine kinase activated by PI3K that regulates cell proliferation, survival, metabolism, and angiogenesis; key component of oncogenic PI3K/AKT/mTOR signaling pathway
CCND1	tRF-19-F8DHXYIV	Yes			ENSG00000110092	cyclin D1 [KO:K04503]	Required for cell cycle G1/S transition. Interact with tumor suppressor protein Rb
CTNNB1	tRF-19-F8DHXYIV	Yes			ENSG00000168036	catenin beta 1 [KO:K02105]	Component of adherens junctions that regulates epithelial cell growth and adhesion, anchors actin cytoskeleton and transmits contact inhibition signals
ERBB2	tRF-19-F8DHXYIV	Yes			ENSG00000141736	erb-b2 receptor tyrosine kinase 2 [KO:K05083] [EC:2.7.10.1]	Binds tightly to other ligand-bound EGF receptor family members to form a heterodimer, and enhancing kinase-mediated activation of downstream signalling pathways
GSTP1	tRF-19-F8DHXYIV	Yes			ENSG00000084207	glutathione S-transferase pi 1 [KO:K23790] [EC:2.5.1.18]	Catalyzes conjugation of hydrophobic/electrophilic compounds with reduced glutathione for detoxification. Polymorphic variants affect xenobiotic metabolism and cancer susceptibility
MAP2K1	tRF-19-F8DHXYIV	Yes			ENSG00000169032	mitogen-activated protein kinase kinase 1 [KO:K04368] [EC:2.7.12.2]	Dual-specificity protein kinase upstream of ERKs in MAPK/ERK cascade, involved in many cellular processes such as proliferation, differentiation, transcription regulation and development
RAF1	tRF-19-F8DHXYIV	Yes			ENSG00000132155	Raf-1 proto-oncogene, serine/threonine kinase [KO:K04366] [EC:2.7.11.1]	Cellular homolog of viral raf gene (v-raf). The encoded protein is a MAP kinase kinase kinase (MAP3K), which functions downstream of the Ras family of membrane associated GTPases to which it binds directly
FOLR3	tRF-17-D7LYR15		Yes		ENSG00000110203	folate receptor gamma [KO:K13649]	Member of folate receptor family; high-affinity binding of folic acid and reduced derivatives; mediates 5-methyltetrahydrofolate uptake into cells

PIK3CD	tRF-23-7SB1RHODV		Yes	ENSG00000171608	phosphatidylinositol-4,5-bisphosphate 3-kinase catalytic subunit delta [KO:K00922] [EC:2.7.1.153]	Class I PI3K (p110δ) found primarily in leukocytes; phosphorylates inositol lipids for immune response
BAD	tRF-25-R9ODMJ6B26	Yes		ENSG00000002330	BCL2 associated agonist of cell death [KO:K02158]	BCL-2 family member that positively regulates apoptosis by forming heterodimers with BCL-xL and BCL-2, reversing their death repressor activity. Proapoptotic activity of this protein is regulated through its phosphorylation
MMP9	tRF-27-M2WEPSJR852	Yes		ENSG00000100985	matrix metallopeptidase 9 [KO:K01403] [EC:3.4.24.35]	Proteins of MMP family are involved in the breakdown of extracellular matrix in normal physiological processes. This enzyme degrades type IV and V collagens
GRM1	tRF-27-M2WEPSJR852	Yes		ENSG00000152822	glutamate metabotropic receptor 1 [KO: K04603]	Activates phospholipase C
HRAS	tRF-27-M2WEPSJR852	Yes		ENSG00000174775	HRas proto-oncogene, GTPase [KO:K02833]	This gene belongs to the Ras oncogene family, whose members are related to the transforming genes of mammalian sarcoma retroviruses. The products encoded by these genes function in signal transduction pathways
PDGFRB	tRF-34-D3KS7SB1RHODE2		Yes	ENSG00000113721	platelet derived growth factor receptor beta [KO:K05089] [EC:2.7.10.1]	Cell surface tyrosine kinase receptor for members of the platelet-derived growth factor family; essential for cardiovascular development and actin cytoskeleton rearrangement
CDKN1A	tRF-34-D3KS7SB1RHODE2		Yes	ENSG00000124762	cyclin dependent kinase inhibitor 1A [KO:K06625]	Potent cyclin-dependent kinase inhibitor that binds and inhibits cyclin-CDK2 and cyclin-CDK4 complexes, regulating G1 cell cycle progression and mediating p53-dependent G1 arrest in response to stress stimuli
**B. PB.PC vs BPH.PA**						
IKBKB	tRF-41-8HM2OSRN2NKSEK51B		Yes	ENSG00000104365	inhibitor of nuclear factor kappa B kinase subunit beta [KO:K07209] [EC:2.7.11.10]	Phosphorylates the inhibitor in the inhibitor/NF-kappa-B complex, causing dissociation of the inhibitor and activation of NF-kappa-B
	tRF-43-3W2VR008R959KUMK9		Yes			
	tRF-45-3W2VR008R959KUMKF6		Yes			
	tRF-45-3W2VR008R9D9KUMKF6		Yes			
PLAU	tRF-43-3W2VR008R959KUMK9		Yes	ENSG00000122861	plasminogen activator, urokinase [KO:K01348] [EC:3.4.21.73]	Secreted serine protease that converts plasminogen to plasmin; involved in fibrinolysis, ECM remodeling, and cell migration.
	tRF-44-NMEH623K76IR3DR2I2		Yes			
	tRF-45-3W2VR008R959KUMKF6		Yes			
	tRF-45-3W2VR008R9D9KUMKF6		Yes			
GRB2	tRF-43-3W2VR008R959KUMK9		Yes	ENSG00000177885	growth factor receptor bound protein 2 [KO:K04364]	Binds the epidermal growth factor receptor and contains one SH2 domain and two SH3 domains that direct complex formation with proline-rich regions of other proteins, linking EGFR to signal transduction
	tRF-45-3W2VR008R959KUMKF6		Yes			
	tRF-45-3W2VR008R9D9KUMKF6		Yes			
CASP9	tRF-43-3W2VR008R959KUMK9		Yes	ENSG00000132906	caspase 9 [KO:K04399] [EC:3.4.22.62]	Plays a central role in apoptosis. Tumor suppressor
	tRF-45-3W2VR008R959KUMKF6		Yes			
	tRF-45-3W2VR008R9D9KUMKF6		Yes			
E2F3	tRF-43-3W2VR008R959KUMK9		Yes	ENSG00000112242	E2F transcription factor 3 [KO:K06620]	Transcription factor that binds DP proteins and interacts with retinoblastoma protein (pRB) to regulate cell cycle gene expression

	tRF-45-3W2VR008R959KUMKF6	Yes			
	tRF-45-3W2VR008R9D9KUMKF6	Yes			
PDGFRB	tRF-43-3W2VR008R959KUMK9	Yes	ENSG00000113721	platelet derived growth factor receptor beta [KO:K05089] [EC:2.7.10.1]	Cell surface tyrosine kinase receptor for members of the platelet-derived growth factor family; essential for cardiovascular development and actin cytoskeleton rearrangement
	tRF-45-3W2VR008R959KUMKF6	Yes			
	tRF-45-3W2VR008R9D9KUMKF6	Yes			
IGF1R	tRF-43-3W2VR008R959KUMK9	Yes	ENSG00000140443	insulin like growth factor 1 receptor [KO:K05087] [EC:2.7.10.1]	This receptor binds insulin-like growth factor with a high affinity. It has tyrosine kinase activity. The IGF1R plays a critical role in transformation events
	tRF-45-3W2VR008R959KUMKF6	Yes			
	tRF-45-3W2VR008R9D9KUMKF6	Yes			
	tRF-43-7Z2R1HPSR9O9337KR	Yes			
	tRF-44-7Z2R1HPSR9O9337K0V	Yes			
	tRF-45-7Z2R1HPSR9O9337KB6	Yes			
RELA	tRF-43-3W2VR008R959KUMK9	Yes	ENSG00000173039	RELA proto-oncogene, NF-kB subunit [KO:K04735]	NF-κB subunit that forms most abundant complex with NFKB1; translocates to nucleus upon inhibitor degradation to activate target gene transcription
	tRF-45-3W2VR008R959KUMKF6	Yes			
	tRF-45-3W2VR008R9D9KUMKF6	Yes			
BCL2	tRF-45-3W2VR008R959KUMKF6	Yes	ENSG00000171791	BCL2 apoptosis regulator [KO:K02161]	Involved in the inhibition of apoptosis
	tRF-44-7Z2R1HPSR9O9337K0V	Yes			
	tRF-45-7Z2R1HPSR9O9337KB6	Yes			
EGFR	tRF-43-7Z2R1HPSR9O9337KR	Yes	ENSG00000146648	epidermal growth factor receptor [KO:K05098] [EC:2.7.10.1]	Cell surface receptor tyrosine kinase that binds epidermal growth factor, inducing receptor dimerization and autophosphorylation, which activates signaling pathways controlling cell proliferation and survival
	tRF-44-7Z2R1HPSR9O9337K0V	Yes			
	tRF-45-7Z2R1HPSR9O9337KB6	Yes			
KRAS	tRF-43-7Z2R1HPSR9O9337KR	Yes	ENSG00000133703	KRAS proto-oncogene, GTPase [KO:K07827]	Member of the small GTPase superfamily. Proto-oncogene
	tRF-44-7Z2R1HPSR9O9337K0V	Yes			
	tRF-45-7Z2R1HPSR9O9337KB6	Yes			
PDGFA	tRF-43-7Z2R1HPSR9O9337KR	Yes	ENSG00000197461	platelet derived growth factor subunit A [KO:K04359]	Encodes platelet-derived growth factor subunit A that homodimerizes or heterodimerizes with subunit B to activate PDGF receptor tyrosine kinases, regulating developmental processes
	tRF-44-7Z2R1HPSR9O9337K0V	Yes			
	tRF-45-7Z2R1HPSR9O9337KB6	Yes			

SOS1	tRF-43-7Z2R1HPSR9O9337KR	Yes	ENSG00000115904	SOS Ras/Rac guanine nucleotide exchange factor 1 [KO:K03099]	Guanine nucleotide exchange factor for RAS proteins, membrane proteins that bind guanine nucleotides and participate in signal transduction pathways
	tRF-44-7Z2R1HPSR9O9337K0V	Yes			
	tRF-45-7Z2R1HPSR9O9337KB6	Yes			
E2F2	tRF-43-7Z2R1HPSR9O9337KR	Yes	ENSG00000007968	E2F transcription factor 2 [KO:K09389]	Member of the E2F family of transcription factors involved in cell cycle control, tumor suppressor protein interaction, and DNA binding through conserved domains
	tRF-44-7Z2R1HPSR9O9337K0V	Yes			
	tRF-45-7Z2R1HPSR9O9337KB6	Yes			
NFKBIA	tRF-43-7Z2R1HPSR9O9337KR	Yes	ENSG00000100906	NFKB inhibitor alpha [KO:K04734]	NF-κB inhibitor with ankyrin repeats; interacts with REL dimers to inhibit NF-κB/REL complexes involved in inflammatory responses. Shuttles between cytoplasm and nucleus
	tRF-44-7Z2R1HPSR9O9337K0V	Yes			
	tRF-45-7Z2R1HPSR9O9337KB6	Yes			
CDK2	tRF-43-7Z2R1HPSR9O9337KR	Yes	ENSG00000123374	cyclin dependent kinase 2 [KO:K02206] [EC:2.7.11.22]	Catalytic subunit of cyclin-dependent kinase complex that regulates progression through the cell cycle
	tRF-44-7Z2R1HPSR9O9337K0V	Yes			
	tRF-45-7Z2R1HPSR9O9337KB6	Yes			
AKT1	tRF-45-3W2VR008R9D9KUMKF6	Yes	ENSG00000142208	AKT serine/threonine kinase 1 [KO:K04456] [EC:2.7.11.1]	Serine/threonine kinase activated by PI3K that regulates cell proliferation, survival, metabolism, and angiogenesis; key component of oncogenic PI3K/AKT/mTOR signaling pathway

Predicted tsRNA–target interactions were identified using tRFtar, RNAHybrid and miRanda; "Yes" indicates support by the corresponding prediction database. Ensembl IDs refer to human genes. KEGG Orthology (KO) and Enzyme Nomenclature (EC) numbers are reported when available to indicate pathway assignment and enzymatic activity.
